# Large-scale pancreatic cancer detection via non-contrast CT and deep learning

**DOI:** 10.1038/s41591-023-02640-w

**Published:** 2023-11-20

**Authors:** Kai Cao, Yingda Xia, Jiawen Yao, Xu Han, Lukas Lambert, Tingting Zhang, Wei Tang, Gang Jin, Hui Jiang, Xu Fang, Isabella Nogues, Xuezhou Li, Wenchao Guo, Yu Wang, Wei Fang, Mingyan Qiu, Yang Hou, Tomas Kovarnik, Michal Vocka, Yimei Lu, Yingli Chen, Xin Chen, Zaiyi Liu, Jian Zhou, Chuanmiao Xie, Rong Zhang, Hong Lu, Gregory D. Hager, Alan L. Yuille, Le Lu, Chengwei Shao, Yu Shi, Qi Zhang, Tingbo Liang, Ling Zhang, Jianping Lu

**Affiliations:** 1 Department of Radiology, Shanghai Institution of Pancreatic Disease, Shanghai, China; 2 DAMO Academy, Alibaba Group, New York, NY USA; 3 Hupan Laboratory, Hangzhou, China; 4 Damo Academy, Alibaba Group, Hangzhou, China; 5 Department of Hepatobiliary and Pancreatic Surgery, First Affiliated Hospital of Zhejiang University, Hangzhou, China; 6 Department of Radiology, First Faculty of Medicine, Charles University and General University Hospital in Prague, Prague, Czech Republic; 7 Department of Radiology, Xinhua Hospital, Shanghai Jiao Tong University School of Medicine, Shanghai, China; 8 Department of Radiology, Fudan University Shanghai Cancer Center, Shanghai, China; 9 Department of Surgery, Shanghai Institution of Pancreatic Disease, Shanghai, China; 10 Department of Pathology, Shanghai Institution of Pancreatic Disease, Shanghai, China; 11 Department of Biostatistics, Harvard University T.H. Chan School of Public Health, Cambridge, MA USA; 12 Department of Radiology, Shengjing Hospital of China Medical University, Shenyang, China; 13 Department of Invasive Cardiology, First Faculty of Medicine, Charles University and General University Hospital in Prague, Prague, Czech Republic; 14 Department of Oncology, First Faculty of Medicine, Charles University and General University Hospital in Prague, Prague, Czech Republic; 15 Department of Radiology, Guangdong Provincial People’s Hospital, Guangzhou, China; 16 Department of Radiology, Sun Yat-Sen University Cancer Center, Guangzhou, China; 17 Department of Radiology, Tianjin Medical University Cancer Institute and Hospital, Tianjin, China; 18 Department of Computer Science, Johns Hopkins University, Baltimore, MD USA

**Keywords:** Computed tomography, Pancreatic cancer, Cancer screening, Machine learning

## Abstract

Pancreatic ductal adenocarcinoma (PDAC), the most deadly solid malignancy, is typically detected late and at an inoperable stage. Early or incidental detection is associated with prolonged survival, but screening asymptomatic individuals for PDAC using a single test remains unfeasible due to the low prevalence and potential harms of false positives. Non-contrast computed tomography (CT), routinely performed for clinical indications, offers the potential for large-scale screening, however, identification of PDAC using non-contrast CT has long been considered impossible. Here, we develop a deep learning approach, pancreatic cancer detection with artificial intelligence (PANDA), that can detect and classify pancreatic lesions with high accuracy via non-contrast CT. PANDA is trained on a dataset of 3,208 patients from a single center. PANDA achieves an area under the receiver operating characteristic curve (AUC) of 0.986–0.996 for lesion detection in a multicenter validation involving 6,239 patients across 10 centers, outperforms the mean radiologist performance by 34.1% in sensitivity and 6.3% in specificity for PDAC identification, and achieves a sensitivity of 92.9% and specificity of 99.9% for lesion detection in a real-world multi-scenario validation consisting of 20,530 consecutive patients. Notably, PANDA utilized with non-contrast CT shows non-inferiority to radiology reports (using contrast-enhanced CT) in the differentiation of common pancreatic lesion subtypes. PANDA could potentially serve as a new tool for large-scale pancreatic cancer screening.

## Abstract (web summary)

A deep learning model provides high accuracy in detecting pancreatic lesions in multicenter data, outperforming radiology specialists.

## Main

Pancreatic ductal adenocarcinoma (PDAC) is the deadliest solid malignancy worldwide, and causes approximately 466,000 deaths per year^1^. Despite the poor prognosis of PDAC, its early or incidental detection has been shown to substantially improve patient survival^2–7^. Recent studies indicate that high-risk individuals with screen-detected PDAC have a median overall survival of 9.8 years, substantially longer than the 1.5 years for those diagnosed outside of surveillance (for example, via standard clinical diagnostic techniques)^6^. As such, screening of PDAC holds the greatest promise to reduce PDAC-related mortality^8^. However, due to the relatively low prevalence of PDAC, effective screening in the general population requires high sensitivity and exceptionally high specificity to mitigate the risk of over-diagnosis. Current screening techniques are limited in this regard, and thus cannot be implemented in the general population as urgently needed^9,10^.

Non-contrast computed tomography (CT) is widely used in physical examination centers and hospitals in low-resource regions. Compared with contrast-enhanced CT (the primary imaging modality for diagnosing PDAC^9^), non-contrast CT exposes patients to lower radiation doses and eliminates the risk of adverse reactions to the contrast agents. In addition to abdominal non-contrast CT routinely used in emergency departments and community hospitals, chest non-contrast CT also can fully or partially scan the pancreas region and is the most frequently performed CT exam (that is, it accounts for nearly 40% of all performed CT exams)^11^ in multiple clinical scenarios, such as for lung cancer screening. Although identifying PDAC from non-contrast CT is challenging even for experienced radiologists, recent studies have shown that artificial intelligence (AI) can match or surpass human experts on various medical image analysis tasks^12–17^; moreover, AI is capable of synthesizing contrast-enhanced medical images from regular images^18–21^. AI-based opportunistic screening^22^ via non-contrast CT has the potential to advance early detection of PDAC in the vast population of asymptomatic patients under several clinical domains, with minimal additional cost and exposure to radiation.

**Fig. 1:**
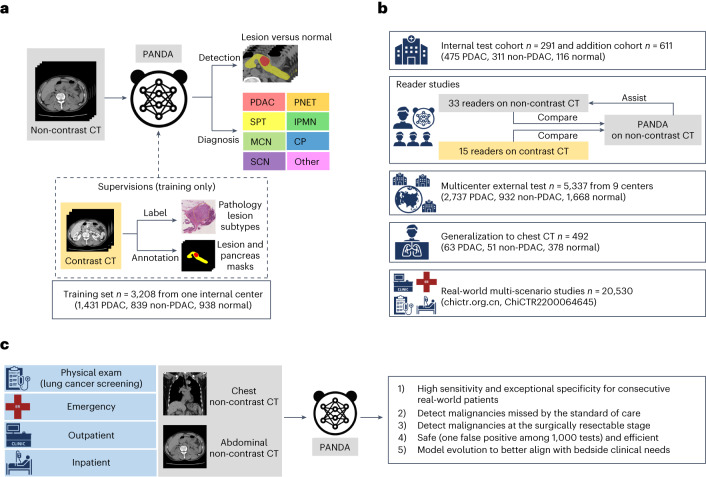
Overview of PANDA’s development, evaluation and clinical translation. **a**, Model development. PANDA takes non-contrast CT as input and outputs the probability and the segmentation mask of possible pancreatic lesions, including PDAC and seven non-PDAC subtypes; PANDA was trained with pathology-confirmed patient-level labels and lesion masks annotated on contrast CT images. CP, chronic pancreatitis. **b**, Model evaluation. We evaluate the performance of PANDA on the internal test cohort, two reader studies (on non-contrast and contrast CT, respectively), external test cohorts consisting of nine centers, a chest CT cohort, and real-world multi-scenario studies (the clinical trial includes two real-world studies; chictr.org.cn, ChiCTR2200064645). **c**, Model clinical translation. The real-world clinical evaluation answers five critical questions to close the clinical translational gap for PANDA.

In this study we show that our proposed AI approach, PANDA (pancreatic cancer detection with AI, Fig. 1), is capable of detecting and diagnosing PDAC and non-PDAC lesions on non-contrast CT with high accuracy and can be readily utilized for opportunistic screening in large-scale asymptomatic patient populations. This will result in safe and effective detection of early-stage malignancies missed by standard of care diagnostic techniques, and in some cases will enable timely treatment with intent to cure. Our study first evaluates PANDA internally on abdominal non-contrast CT scans and compares its performance with results from two reader studies involving 48 radiologists on non-contrast and contrast CT imaging, respectively. We then validate PANDA on a large external multicenter test cohort (*n* = 5,337) to assess its generalizability to various settings. To increase the range of applicable patient populations, we study the feasibility of applying PANDA on chest CT. Finally, to validate the critical issues related to realistic clinical translation, we explore the integration of PANDA into large-scale real-world multi-scenarios of routine clinical processes, involving 20,530 consecutive patients from four settings (that is, physical exam, emergency, outpatient, and inpatient) with available abdominal or chest non-contrast CT scans.

## Results

### The PANDA network

We present a deep learning model, PANDA, to detect and diagnose PDAC and seven subtypes of non-PDAC lesions (Methods), that is, pancreatic neuroendocrine tumor (PNET), solid pseudopapillary tumor (SPT), intraductal papillary mucinous neoplasm (IPMN), mucinous cystic neoplasm (MCN), serous cystic neoplasm (SCN), chronic pancreatitis, and ‘other’ (cf. Supplementary Table 1), from abdominal and chest non-contrast CT scans. Our model can detect the presence or absence of a pancreatic lesion, segment the lesion, and classify the lesion subtypes (Fig. 1a).

PANDA was trained on a training set of abdominal non-contrast CT scans of 3,208 patients from a high-volume pancreatic cancer institution, Shanghai Institution of Pancreatic Diseases (SIPD), directly affiliated with a tertiary hospital (a major comprehensive academic medical center in Shanghai, China). The patient characteristics are listed in Extended Data Table 1. The ground truth labels were confirmed either by surgical pathology for lesions or by a 2 year follow-up for normal controls. PANDA was also supervised by pixel-wise annotations, including both the pancreas and the lesion, transferred by image registration from annotations on paired contrast-enhanced CT scans in which tumors were more visible. Dataset and annotation details are given in the Methods section.

**Extended Data Fig. 1:**
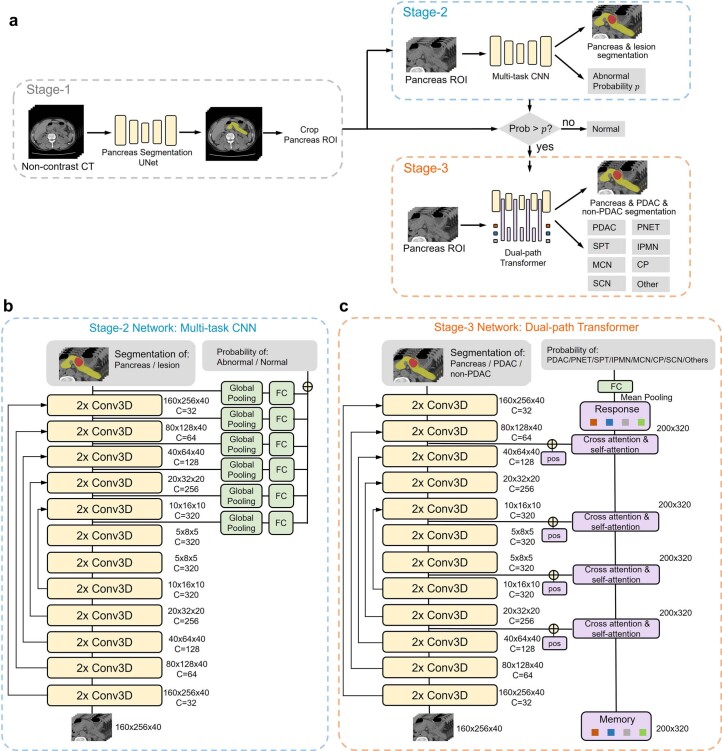
Network architecture. a, Overview. Our deep learning framework consists of three stages: pancreas localization using a segmentation UNet, abnormality detection using a multi-task CNN, and lesion subtype classification using a dual-path transformer. b, Architecture of the multi-task CNN for Stage-2. We extract multi-level features from the segmentation UNet, and concatenate the features after global pooling for abnormal and normal classification. c, Architecture of the dual-path transformer for Stage-3. Lesion-related features are encoded into the learnable memory vectors from the UNet features and the learnable positional embeddings using cross-attention and self-attention. The response vectors of this procedure are then used for the classification of PDAC and seven non-PDAC subtypes.

PANDA consists of a cascade of three network stages that increase in model complexity and the difficulty level of the tasks performed (Extended Data Fig. 1; Methods). The first stage (Stage 1) involves pancreas localization, using an nnU-Net model^23^. The second stage (Stage 2) carries out lesion detection, and we build convolutional neural networks (CNNs) together with a classification head to distinguish the subtle texture change of lesions in non-contrast CT. We tune the Stage 2 model to achieve a specificity of 99% for lesion detection on cross-validation of the training set to reduce false-positive predictions. The third stage (Stage 3) involves the differential diagnosis of pancreatic lesions if any abnormalities are detected in the second stage, integrated with an auxiliary memory transformer branch^24,25^ to automatically encode the feature prototypes of the pancreatic lesions, such as local texture, position and pancreas shape, for more accurate fine-grained classification.

We mainly evaluate the performance of PANDA on three tasks (Methods). The first task is lesion detection: that is, lesion versus normal, which also includes detection rates stratified by lesion type and by cancer stage. The second task is primary diagnosis: PDAC versus non-PDAC versus normal, which also includes evaluation of one versus others, for example, PDAC identification (PDAC versus non-PDAC + normal). The third task is differential diagnosis: that is, classification of PDAC and seven non-PDAC lesion subtypes.

### Internal evaluation

**Fig. 2:**
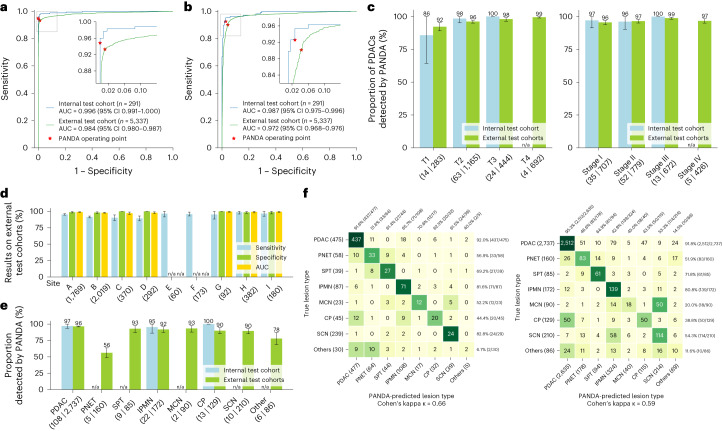
Internal and external validation. **a**,**b** Receiver operating characteristic curves of lesion detection (**a**) and PDAC identification (**b**) for the internal and external test cohorts. **c**, Proportion of PDACs detected by PANDA in terms of American Joint Committee on Cancer (AJCC) T stage (left) and TNM (tumor, nodes, metastasis) stage (right) in the internal test cohort (*n* = 105) and external test cohort (*n* = 2,584). **d**, Sensitivity, specificity and AUC of lesion detection in the external center cohorts (sites A–I, *n* = 5,337). **e**, Proportion of different lesion subtypes detected by PANDA in the internal test cohort (*n* = 175) and external test cohort (*n* = 3,669). **f**, Confusion matrices of differential diagnosis in the internal differential diagnosis cohort (left) and external test cohorts (right). **c**–**e**, Error bars indicate 95% CI. The center shows the computed mean of the metric specified by its respective axis labels. The results of subgroups with too few samples to be studied reliably (≤10) are omitted and marked as not applicable (n/a).

Our independent internal test cohort consisted of 291 patients (108 patients with PDAC, 67 patients with non-PDAC, and 116 normal controls) from the SIPD (Extended Data Table 1; Methods). These patient labels were confirmed on surgical pathology or a 2 year follow-up. For lesion detection, PANDA achieved an area under the receiver operating characteristic curve (AUC) of 0.996 (95% confidence interval (CI) 0.991–1.00, Fig. 2a), a sensitivity of 94.9% (95% CI 91.4–97.8%) and a specificity of 100% (95% CI 100–100%); for the PDAC subgroup the sensitivity for detection was 97.2% (95% CI 93.5–100%) overall, 97.1% (95% CI 91.4–100%; *n* = 35; Fig. 2c) for stage I, and 96.2% (95% CI 90.4–100%; *n* = 52; Fig. 2c) for stage II. For small PDACs (diameter <2 cm, T1 stage), the sensitivity for detection was 85.7% (95% CI 64.3–100%; *n* = 14; Fig. 2c). For PDAC identification, the AUC was 0.987 (95% CI 0.975–0.996, Fig. 2b), the sensitivity was 92.6% (95% CI 87.3–97.0%) and the specificity was 97.3% (95% CI 94.6–99.5%, Fig. 2b).

For the internal differential diagnosis cohort (*n* = 786; Extended Data Table 1; Methods), PANDA achieved an accuracy of 79.6% (95% CI 76.8–82.6%) and a balanced accuracy (averaged class-level accuracy) of 60.7% (95% CI 55.7–65.4%). The accuracy is non-inferior (*P* = 0.0018 at a pre-specified 5% margin) to the second-reader radiology reports (Fig. 2f, Supplementary Fig. 1 and Supplementary Table 4), which is a secondary analysis of a primary standard of care clinical radiology report that includes access to the contrast-enhanced CT, clinical information and patient history, and represents the standard of care of pancreatic lesion management practice in the internal center. The results for IPMN subtype classification (main or mixed-duct versus branch-duct IPMN) and the full pipeline (detection + diagnosis) in the internal cohorts are given in Supplementary Table 13 and Supplementary Fig. 7a, respectively.

**Extended Data Fig. 2:**
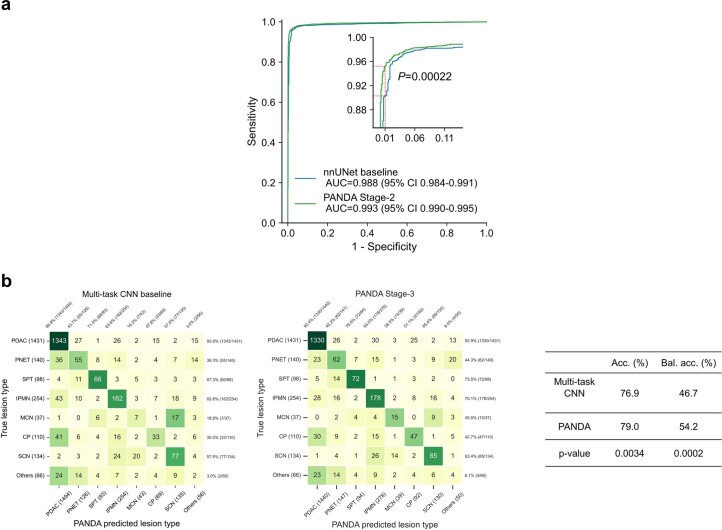
Ablation studies of the 5-fold cross-validation on the training set (n = 3,208). a, nnUNet vs. PANDA Stage-2 network (multi-task CNN) for lesion detection, where PANDA achieved significant improvement in AUC score (P = 0.00022). At the same (desired) specificity level of 99.0%, PANDA Stage-2 outperformed nnUNet in sensitivity by 4.9% (95.2% vs. 90.3%) (marked in red dotted line). b, Multi-task CNN baseline (same as PANDA Stage-2 network with nnUNet backbone and classification head) vs. PANDA Stage-3 (dual-path transformer) for differential diagnosis, where PANDA achieved significant improvement in both accuracy (Acc.) and balanced accuracy (Bal. acc.). The significance test comparing the AUCs of the AI model and nnUNet is conducted using the Delong test. Two-sided permutation tests were used to compute the statistical differences of accuracy and balanced accuracy.

**Extended Data Fig. 3:**
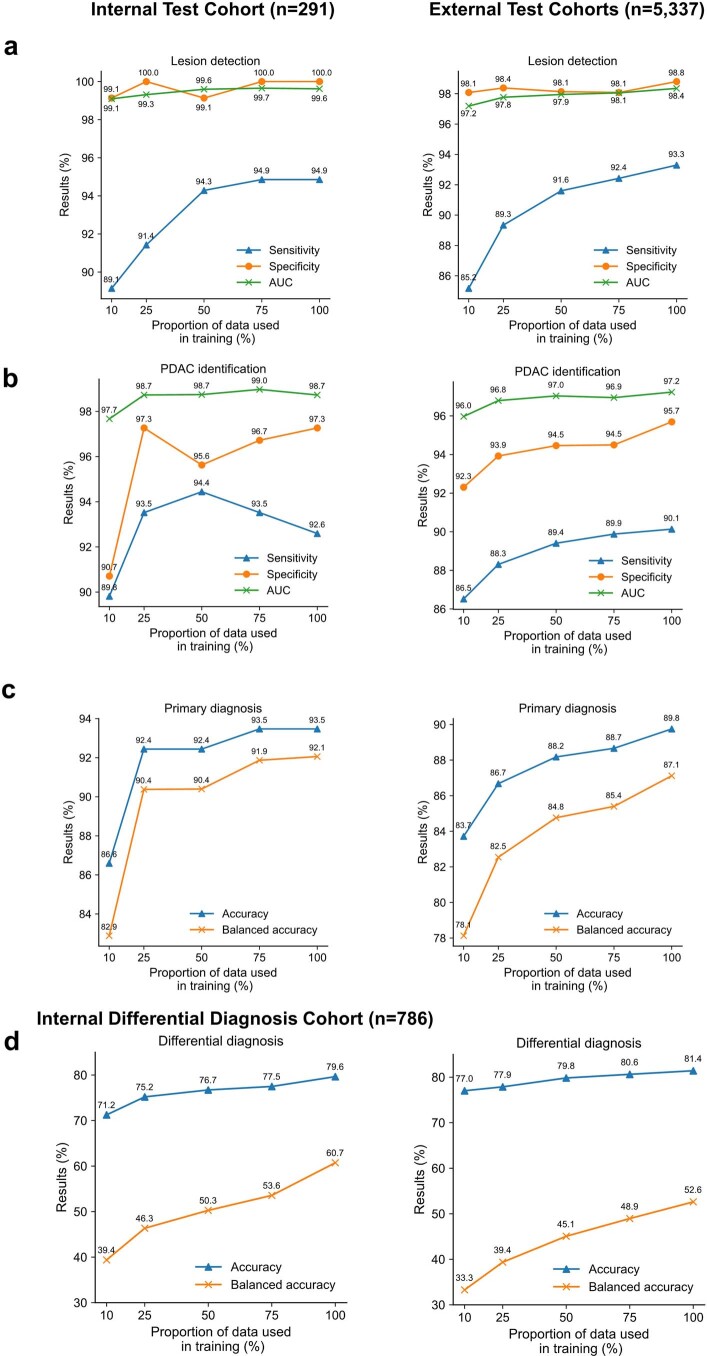
Influence of the proportion of training data. Influence of the proportion of training data tested on the internal test cohort (left) and the external test cohorts (right) on the task of a, lesion detection b, PDAC identification c, primary diagnosis d, differential diagnosis.

**Extended Data Fig. 4:**
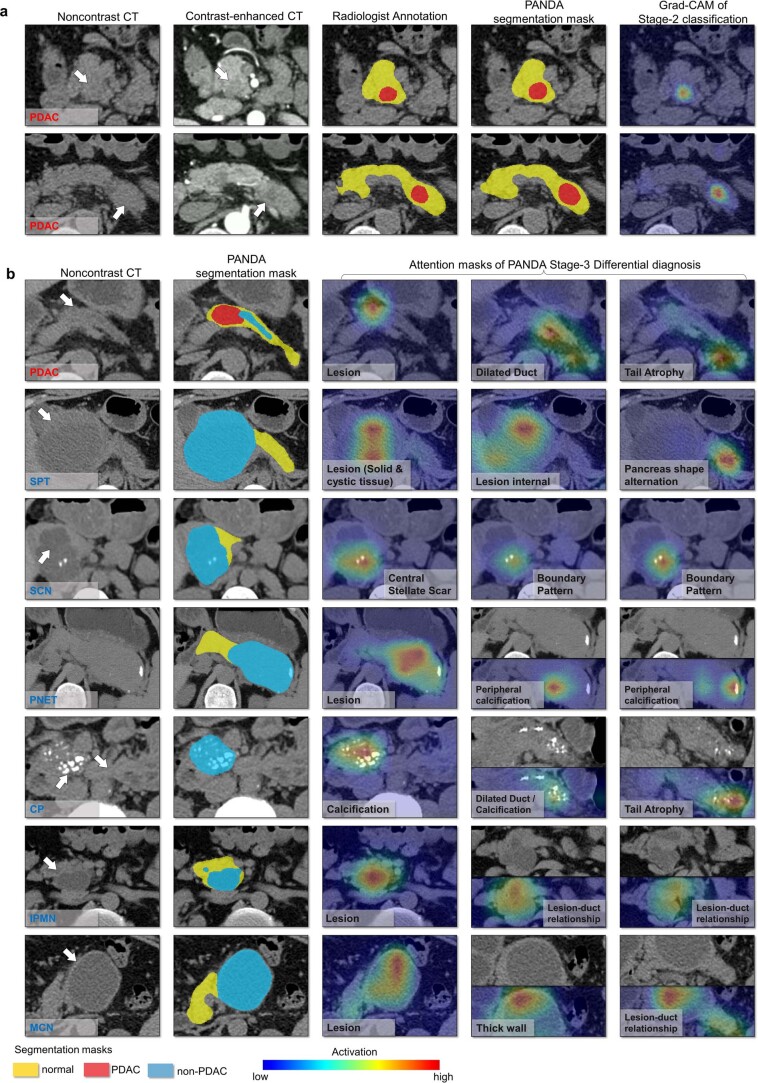
Analysis of interpretability. a, we visualize the noncontrast CT, contrast-enhanced CT, and the radiologist’s annotated mask and compare them with the PANDA segmentation map and the Grad-CAM heatmap of PANDA Stage-2 classification for lesion detection. PANDA correctly predicted the position of the PDAC (PANDA segmentation map) and made positive classification based on the local features of the PDAC (Grad-CAM heatmap). b, we visualize the top activated attention maps of the Transformer branch of PANDA Stage-3 to interpret how PANDA classified the lesions. The memory tokens of the Transformer not only attended to the lesion locations but also considered the secondary signs for lesion diagnosis as utilized by the radiologists. E.g. A PDAC caused pancreatic duct dilation and pancreatic atrophy; A SPT was circumscribed with the heterogeneity of both solid and cystic regions; A SCN had a pattern of central stellate scar and so-called honeycomb pattern; A PNET had isoattenuating mass and peripheral calcification; A CP was associated with calcification, dilated duct, and pancreatic atrophy; An IPMN lesion was connected to the pancreatic duct; A MCN had the thick cystic wall and no visual connection with the pancreatic duct. The heatmaps of multiple slices were displayed for the CP, IPMN, and MCN.

Ablation studies were conducted to analyze the performance of PANDA’s Stage 2 and Stage 3 modules on the internal training cohort (*n* = 3,208) (Extended Data Fig. 2; Methods). Stage 2 and Stage 3 had significantly better performance than their related baseline methods (*P* = 0.00022 and *P* = 0.0002, respectively). We also analyzed the effect of training data size on the performance of PANDA. More training data led to better performance for all tasks, and the margins of improvement increased as the tasks became more challenging (Extended Data Fig. 3). PANDA is an interpretable AI model that directly outputs the segmentation mask of the pancreas and the detected lesion (see Supplementary Table 5 for segmentation accuracy). Additional analyses of interpretability via the visualization of the Stage 2 activation maps and Stage 3 attention maps are provided in Extended Data Fig. 4 and the Methods section.

### Reader studies

We conducted two reader studies (Methods and Extended Data Table 2). The aim of the first study was to compare PANDA with non-contrast CT readers consisting of pancreatic imaging specialists, general radiologists and radiology residents, and validate whether PANDA could assist them in making more accurate decisions. The second reader study was designed to compare PANDA, using only non-contrast CT, with a clinical expert upper-bound set-up, that is, a pancreatic imaging specialist reading a contrast-enhanced CT.

**Fig. 3:**
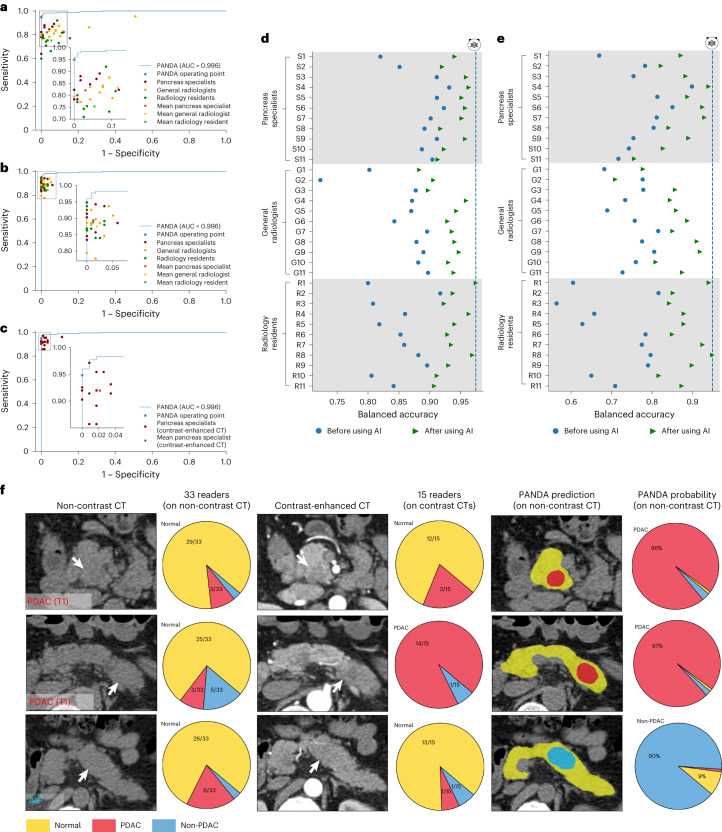
Reader studies. **a**, Comparison between PANDA and 33 readers with different levels of expertise on non-contrast CT for lesion detection. **b**, Lesion detection performance of the same set of readers with the assistance of PANDA on non-contrast CT. **c**, Comparison between PANDA using non-contrast CT and 15 pancreas specialists using contrast-enhanced CT for lesion detection. **d**,**e**, Balanced accuracy improvement in radiologists with different levels of expertise for lesion detection (**d**) and PDAC identification (**e**). **f**, Examples of early-stage PDACs and a case of autoimmune pancreatitis (AIP) missed by readers on non-contrast CT and on contrast CT but detected by PANDA.

In the first reader study, 33 readers from 12 institutions interpreted 291 non-contrast CT scans in the internal test cohort. Alongside the CT images, readers were provided with each patient’s age and sex, and rated each case as PDAC, non-PDAC or normal (Supplementary Fig. 2). For lesion detection, the performance values of all 33 readers fell below PANDA’s receiver operating characteristic (ROC) curve (Fig. 3a). PANDA significantly outperformed the average reader performance by 14.7% (95% CI 10.8–18.8%, *P* = 0.0002) in sensitivity and 6.8% (95% CI 5.6–8.1%, *P* = 0.0002) in specificity for lesion detection (Supplementary Table 6a), and by a significant margin of 34.1% (95% CI 29.3–38.9%, *P* = 0.0002) in sensitivity and 6.3% (95% CI 4.1–8.4%, *P* = 0.0002) in specificity for PDAC identification (Supplementary Table 6b). Notably, for PDAC identification the sensitivity was as low as 16.7–35.2% for some radiology residents who were not specialized in pancreatic imaging.

After at least a 1 month washout period, readers were additionally provided with the AI lesion segmentation and primary diagnosis probabilities (Supplementary Fig. 3) and re-rated each patient. With AI assistance, for lesion detection the mean reader performance was significantly improved by 8.5% in sensitivity (95% CI 6.5–10.3%, *P* = 0.0002) and 5.3% in specificity (95% CI 4.3–6.3%, *P* = 0.0002; Supplementary Table 7a). For PDAC identification, the mean reader performance was significantly improved by 20.5% (95% CI 17.8–23.4%, *P* = 0.0002) in sensitivity and by 3.1% (95% CI 2.1−4.1%, *P* = 0.0002) in specificity (Supplementary Table 7b). Overall, the largest improvement was observed in readers not specialized in pancreatic imaging. The residents’ performance with AI could even approach that of pancreatic radiology specialists (evaluated using balanced accuracy in Fig. 3d,e and Supplementary Tables 7 and 9). Detailed confusion matrices are given in Supplementary Figs. 2 and 4.

In the second reader study, another 15 pancreatic imaging specialists from the internal center (SIPD) interpreted multi-phase contrast-enhanced CT scans of the same 291 patients. Each reader was provided with the non-contrast, arterial, and venous phase of CT images along with the age and sex information and carried out the same rating (Supplementary Fig. 5). PANDA (on non-contrast CT imaging) did better than the mean performance of the specialists (using contrast-enhanced CT scans) by 2.9% (95% CI 0.1–5.8%, *P* = 0.0002 for non-inferiority) in sensitivity and by 2.1% (95% CI 1.4–3.0%, *P* = 0.0002 for difference) in specificity, for lesion detection (Supplementary Tables 10a and 11a); and by a margin of 13.0% (95% CI 8.5–17.8%, *P* = 0.0002 for difference) in sensitivity and 0.5% (95% CI −0.7 to 1.9%, *P* = 0.0002 for non-inferiority) in specificity, for PDAC identification (Supplementary Tables 10b and 11b).

### Generalization to external multicenter test cohorts

To assess the generalizability of PANDA to different patient populations and imaging protocols we validated our model on external multicenter (*n* = 9) test cohorts, which consisted of preoperative non-contrast abdominal CT scans of 5,337 patients (2,737 with PDAC, 932 with non-PDAC and 1,668 normal controls) from China, Taiwan ROC and the Czech Republic (Extended Data Table 1; Methods). The patient labels were confirmed by surgical or biopsy pathology diagnosis reports or a 2 year follow-up visit diagnosis. PANDA achieved an AUC of 0.984 (95% CI 0.980–0.987, Fig. 2a), sensitivity of 93.3% (95% CI 92.5–94.1%) and specificity of 98.8% (95% CI 98.3–99.4%) for lesion detection. For the PDAC patient subgroup, the detection rate was 96.5% (95% CI 95.8–97.2%) overall, 95.6% (95% CI 93.9–97.0%; Fig. 2c) for stage I, and 96.5% (95% CI 95.3–97.8%; Fig. 2c) for stage II. For small PDAC lesions (diameter <2 cm, T1 stage), the sensitivity for detection was 92.2% (95% CI 89.0–95.4%; *n* = 283; Fig. 2c). The lesion detection results for each center are shown in Fig. 2d and the performance stratified by lesion subtype is given in Fig. 2e. For PDAC identification, the sensitivity was 90.1% (95% CI 89.0–91.2%) and the specificity was 95.7% (95% CI 94.9–96.5%; Fig. 2b).

For differential diagnosis (Fig. 2f, *n* = 3,669) our model achieves an accuracy of 81.4% (95% CI 80.2–82.6%) and a balanced accuracy of 52.6% (95% CI 50.0–55.1%). The confusion matrices, accuracy and balanced accuracy of each external center with pathology-confirmed lesion types are shown in Supplementary Fig. 6 and Supplementary Table 12. The results for IPMN subtype classification and the full pipeline are given in Supplementary Table 13 and Supplementary Fig. 7b, respectively.

### Feasibility study of lesion detection on chest computed tomography

**Fig. 4:**
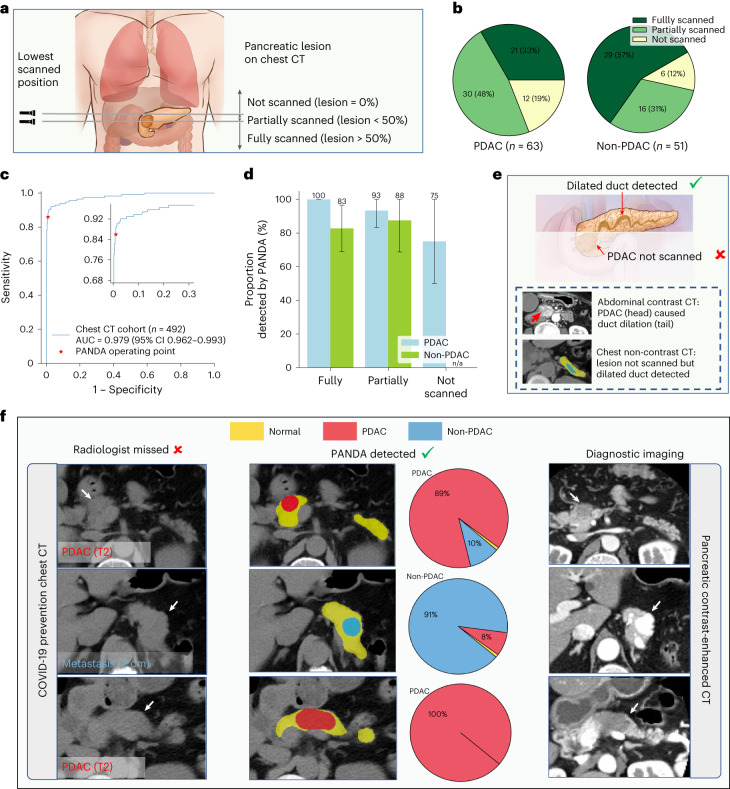
Validation on chest non-contrast CT. **a**, Schematic diagram of the proportion of the pancreatic lesion scanned in chest non-contrast CT. We categorize all cases into three categories, that is, lesion not scanned, lesion partially scanned, and lesion fully scanned, based on the relative position of the lowest scanned slice and the lesion. **b**, The proportion of the three categories in PDAC and non-PDAC cases. **c**, ROC curve for lesion detection on non-contrast chest CT. **d**, Proportion of lesions detected by PANDA in the PDAC (*n* = 63) and non-PDAC cases (*n* = 51). Error bars indicate 95% CI. The center shows the computed mean of the metric specified by the respective axis labels. The results of subgroups with too few samples to be studied reliably (≤10) are omitted and marked as ‘n/a’. **e**, Illustration of how PANDA can detect lesions that are not scanned in chest CT. Two scans of the same patient showing that PANDA can detect dilated pancreatic duct (usually caused by PDAC) even when the PDAC is not scanned. **f**, PANDA can detect early-stage PDACs and metastatic cancer that was initially misdetected by the radiologists on chest non-contrast CT (COVID-19 prevention CT).

PANDA’s ability can be coupled with established clinical indications such as chest CT for lung cancer screening. We validated the feasibility of pancreatic lesion detection using PANDA on chest CT (Fig. 4). From SIPD we collected non-contrast chest CT scans of 492 patients, consisting of 63 with PDAC, 51 with non-PDAC, and 378 normal controls, as a test cohort independent of the training data. The patient labels were confirmed by surgical pathology or a 2 year follow-up visit diagnosis (Methods).

Without tuning on any chest CT scans, PANDA achieved an AUC of 0.979 (95% CI 0.962–0.993), a sensitivity of 86.0% (95% CI 79.4–91.9%) and a specificity of 98.9% (95% CI 97.8–100%) for lesion detection (Fig. 4c), and a sensitivity of 92.1% (95% CI 85.7–98.4%) for the PDAC subgroup. Depending on detailed chest CT protocols, certain pancreatic lesions could not be completely scanned. We analyzed the lesion scanning completeness in chest CT by referring to the lesion location in contrast-enhanced abdominal CT scans (Fig. 4a), and found that 67% of patients with PDAC and 43% of patients with non-PDAC were not fully scanned (Fig. 4b). For those patients whose pancreatic lesions were not captured in the CT scan’s field of view (and thus were not directly observable), 75% of PDAC cases in these patients were detected by PANDA, that is, the patients were classified as having a lesion (Fig. 4d) via secondary signs of the disease such as dilation of the pancreatic duct (Fig. 4e).

### Real-world clinical evaluation

The above experiments validate the clinical utility of PANDA, but they are limited to pathology-confirmed pancreatic lesions (thus with higher risk) and a moderate number of normal cases. It is unclear whether PANDA could be generalized well to the real-world population, including patients with lesions of lower risk (for example, chronic pancreatitis and branch-duct IPMN) and the large, diverse set of subjects with normal pancreas. To close the clinical translation gap, evaluation in real-world application settings is required to answer the following critical questions: first, what is the true performance of PANDA when used for consecutive real-world patient populations, possibly containing unseen lesion subtypes, collected from varying CT imaging protocols and clinical scenarios (that is, physical examination, emergency, outpatient, and inpatient); second, can the tool detect malignancies that were not previously detected by the standard of care clinical diagnosis; third, can patients benefit from such detection (for example, if the malignancy was detected at its surgically resectable stage^26^); fourth, can the tool be clinically safe and efficient, without a large number of false-positive findings that require unnecessary follow-up tests and extra time for being ruled out; and last, can the benchtop-derived AI be further improved according to bedside clinical requirements^27^.

**Fig. 5:**
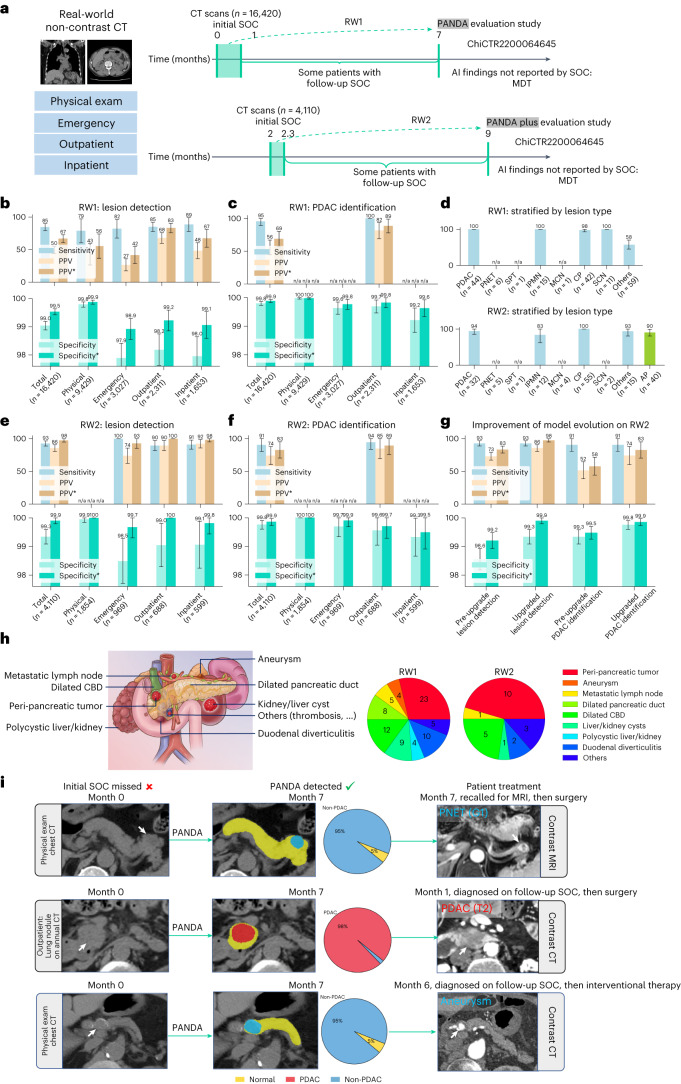
Real-world clinical evaluation. **a**, The data collection process of two real-world datasets, that is, RW1 and RW2, for the original PANDA model and the upgraded PANDA Plus model, respectively. SOC, standard of care. **b**,**c**,**e**,**f**, The sensitivity, specificity and PPV on RW1 (*n* = 16,420) and RW2 (*n* = 4,110). The superscript * represents adjusted results if we exclude cases of (peri-)pancreatic findings. **d**, Proportion of different lesion types detected in RW1 (*n* = 179) and RW2 (*n* = 166). **g**, The comparison between PANDA and PANDA Plus on RW2 (*n* = 4,110). Error bars indicate 95% CI. The center shows the computed mean of the metric specified by the respective axis labels. The results of subgroups with too few samples to be studied reliably (≤10) are omitted and marked as ‘n/a’. **h**, Examples of (peri-)pancreatic findings (left) and the number detected by PANDA (right). CBD, common bile duct. **i**, Examples of cases in which the lesion was missed by the initial SOC but was detected by PANDA.

**Extended Data Fig. 5:**
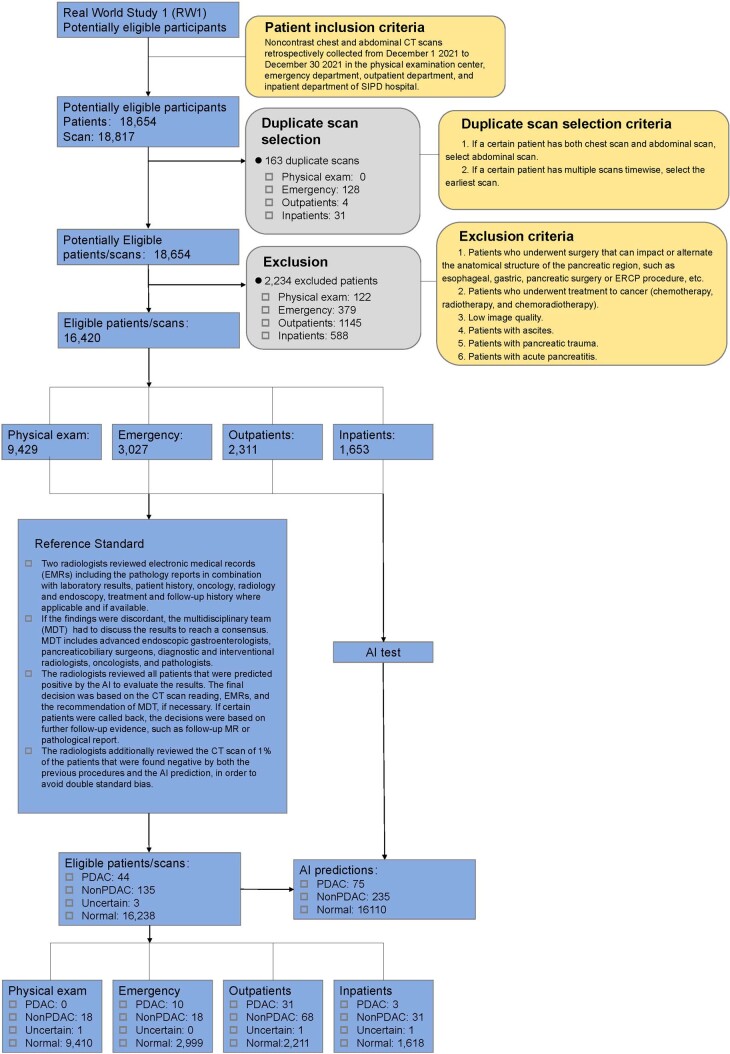
**Overview of the workflow of the first real-world study (RW1).**

**Extended Data Fig. 6:**
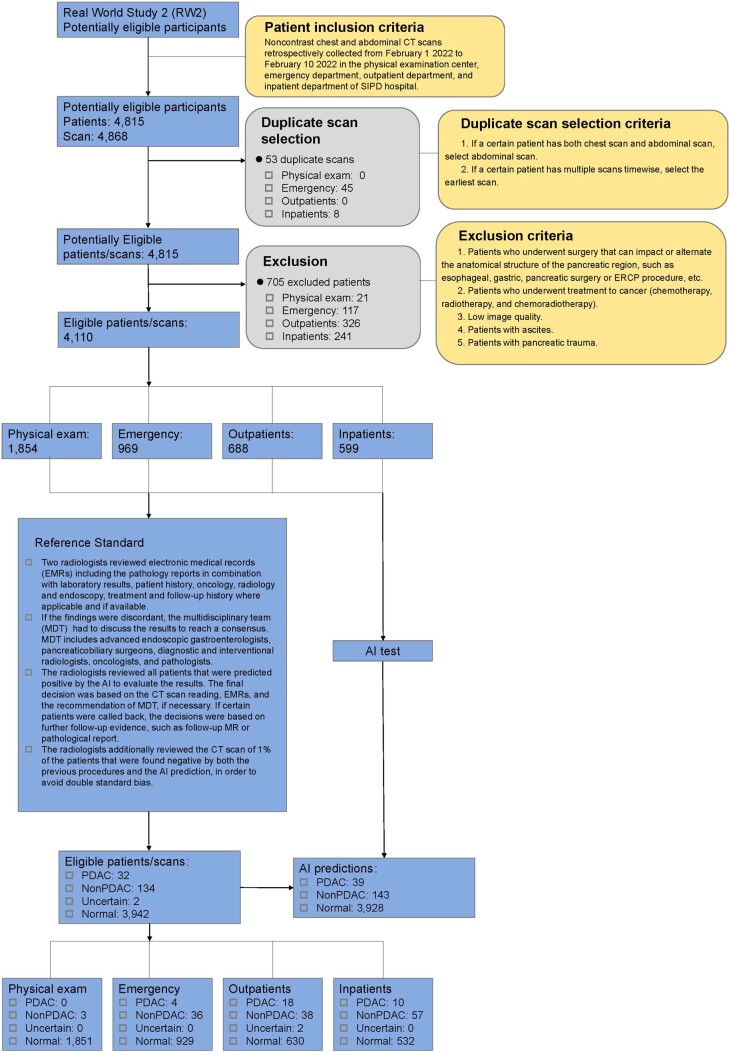
**Overview of the workflow of the second real-world study (RW2).**

We deployed PANDA at the SIPD by seamlessly integrating it into the existing clinical infrastructure and workflow (Supplementary Fig. 9 and Methods ‘Real-world deployment’), and performed two rounds of large-scale, real-world, retrospective studies enrolling consecutive patients (clinical trial ChiCTR2200064645, chictr.org.cn; includes both studies) (Fig. 5a, Extended Data Fig. 5 and Extended Data Fig. 6; Methods). Due to the retrospective nature of the study we used two timeframes for the standard of truth for each patient (Fig. 5a): the initial standard of care, that is, the clinical diagnosis at the initial visit when the non-contrast CT was acquired; and the follow-up standard of care, that is, the clinical diagnosis obtained at follow-up (after the initial visit and before the PANDA evaluation study).

#### First real-world evaluation cohort

For the first real-world evaluation cohort (RW1, *n* = 16,420), the first four questions were assessed: performance; change in standard of care diagnosis; patient benefit; and safety and efficiency.

##### Performance

RW1 included 44 PDACs and 135 non-PDACs. For lesion detection, PANDA achieved an overall sensitivity of 84.6% (95% CI 79.4%–89.9%) and specificity of 99.0% (95% CI 98.9%–99.2%) (Fig. 5b), and for PDAC identification PANDA achieved an overall sensitivity of 95.5% (95% CI 89.3%–100%), specificity of 99.8% (95% CI 99.7%–99.9%) and positive predictive value (PPV) of 56.0% (95% CI 44.8%–67.2%) (Fig. 5c). Of the four scenarios (that is, physical examination, emergency, outpatient, and inpatient), inpatient had the highest sensitivity of 88.6% (95% CI 78.0%–99.1%) and physical examination had the highest specificity of 99.8% (95% CI 99.7%–99.9%), for lesion detection. The multi-disciplinary team found that 51% (80 of 156) of the false positives by AI were actually (peri-)pancreatic diseases (Fig. 5h and Supplementary Fig. 8) requiring attention from radiologists^28^. Considering that these findings might be signs of pathology, they were excluded from the results, which were adjusted as below: for lesion detection the overall adjusted specificity increased to 99.5% (Fig. 5b), and for PDAC identification the adjusted specificity increased to 99.9% and the adjusted PPV, to 68.9% (Fig. 5c). More detailed results are shown in Supplementary Figs. 10–14.

##### Change in standard of care diagnosis

PANDA detected 26 pancreatic lesions that were not detected by the initial standard of care (Fig. 5i and Extended Data Table 3), consisting of 1 PDAC, 1 PNET, 3 IPMNs, 1 metastatic cancer, 6 cases of pancreatitis, 1 peri-pancreatic tumor and 13 SCN/cysts (10–33 mm). The opportunistic screening with PANDA could advance the early detection of (peri-)pancreatic malignancies and high-risk lesions.

##### Patient benefit

**Extended Data Fig. 7:**
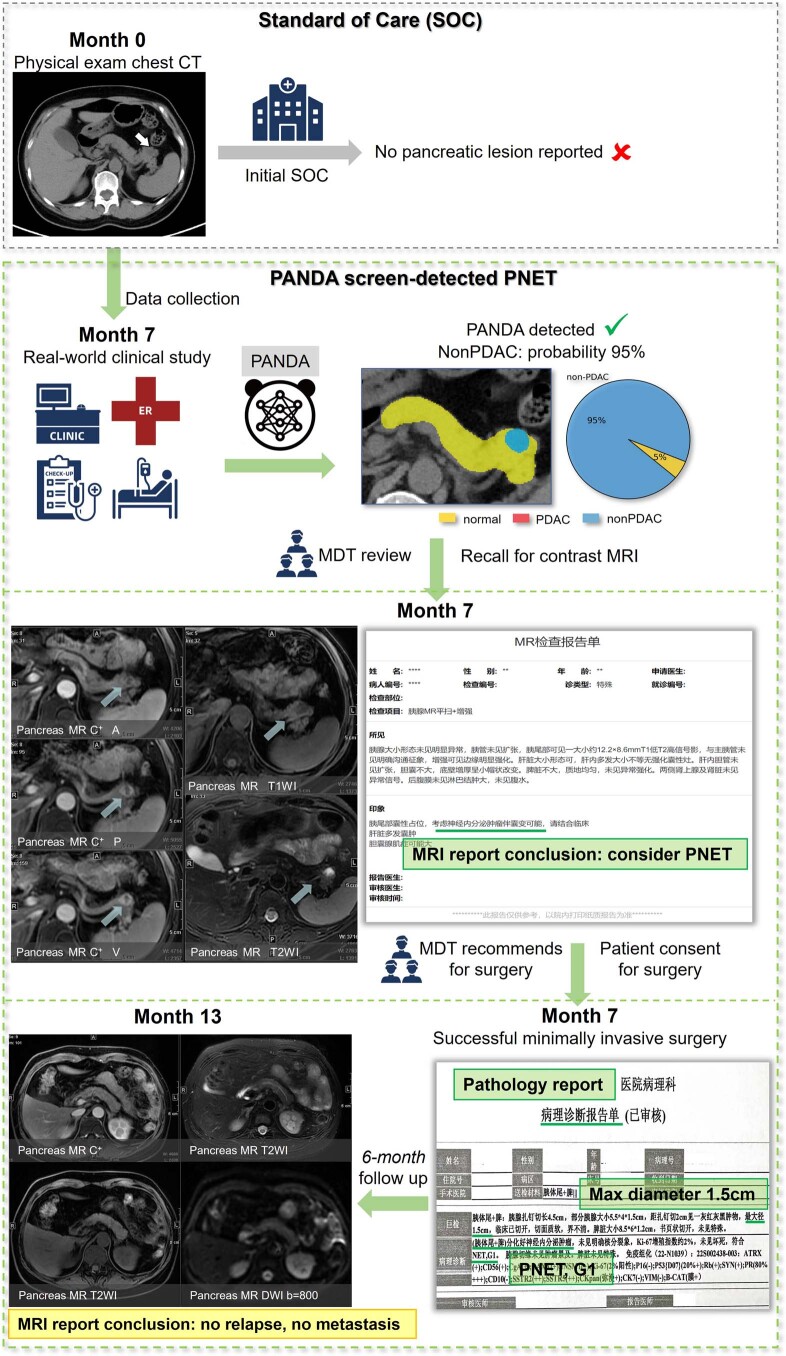
Flowchart describing the successful discovery and intervention of a patient with pancreatic neuroendocrine tumors (PNET) in the real-world clinical evaluation. Noncontrast chest CT was performed on this patient in the physical examination center in Month 0, where the standard of care did not report any pancreatic findings. This patient was included in the real-world study in Month 7 and was reported as non-PDAC (95% probability) by PANDA. After the case was reviewed by MDT, the patient was recalled for contrast-enhanced MRI and was considered as PNET in the radiology report. The patient consented to surgery, which was later successfully performed in Month 7. The post-surgical pathology report confirmed an early-stage PNET (G1, 1.5cm). The 6 month follow-up (Month 13) showed no relapse or metastasis. The English translation of the MRI and pathology reports’ key results are provided in green boxes.

Of the aforementioned 26 lesions first detected by PANDA, eight were detected by follow-up standard of care before this retrospective study, including one T2 stage PDAC and one aneurysm (Fig. 5i and Extended Data Table 3). Nevertheless, earlier detection of some of these lesions by PANDA might benefit the patients’ management and treatment. The remaining patients were invited to undergo magnetic resonance imaging (MRI) but only one (a 57-year-old) complied (due to the COVID-19 pandemic), undergoing contrast-enhanced MRI followed by minimally invasive surgery with curative intent (Extended Data Fig. 7). The surgical pathology confirmed the lesion as a G1 PNET with a size of 1.5 cm.

##### Safety and efficiency

Only 0.5% of patients (*n* = 76) had false-positive AI findings (Supplementary Table 14), of which 92% (70 of 76) were easy to rule out by the radiologists. Of the false negatives (*n* = 28), 89% were benign cysts (*n* = 25), most of which (*n* = 19) were < 10 mm in diameter; the remaining three consisted of a PNET, a case of chronic pancreatitis, and a lesion of undetermined type.

#### Second real-world evaluation cohort

To further optimize PANDA for real-world usage, that is, reduce false positives and enable the detection of previously unseen disease types (for example, acute pancreatitis in the emergency scenario), we utilized hard example mining and incremental learning to upgrade PANDA (the resulting model is named PANDA Plus; see Methods). PANDA Plus was evaluated on the second real-world evaluation cohort (RW2, *n* = 4,110) to assess the fifth question regarding improvement of the model.

##### Model evolution

RW2 included 32 PDACs and 134 non-PDACs. PANDA Plus retained the same sensitivity as PANDA but significantly reduced the false positives by more than 80%, reaching an adjusted specificity of 99.9% for both lesion detection (95% CI 99.8%–100%) and PDAC identification (95% CI 99.7%–100%) (Fig. 5e–g and Supplementary Figs. 15–19). In addition, for the newly learned disease type (that is, acute pancreatitis), the sensitivity for detection was 90.0% (95% CI 80.7%–99.3%) for the 40 patients with acute pancreatitis (Fig. 5d). PANDA Plus detected five pancreatic lesions that were missed by the initial standard of care, consisting of 1 PDAC (T2 stage), 1 PNET and 3 cysts (10–32 mm) (Extended Data Table 3). In addition, the real-world evaluation showed that PANDA maintained robust performance in low-risk lesions despite being originally trained on surgical pathology-confirmed lesions. Specifically, our model had a sensitivity of 92.6% for detecting IPMN and 99.0% for chronic pancreatitis in RW1 and RW2 combined (Fig. 5d), although 22 (81%) of the 27 IPMNs and 94 (97%) of the 97 cases of chronic pancreatitis were not biopsied or resected.

## Discussion

We present PANDA, an AI model that detects the seven most common pancreatic lesions and ‘other’, and diagnoses the lesion subtypes in routine non-contrast CT scans. This task has long been considered impossible for radiologists and, as such, contrast-enhanced CT and/or MRI and endoscopic ultrasound (EUS) have been used as the recognized and recommended diagnostic imaging modalities. We show that by curating a large dataset covering common pancreatic lesion types confirmed by pathology, transferring lesion annotations from contrast-enhanced to non-contrast CT, designing a deep learning approach that incorporates a cascade network architecture for lesion detection and a memory transformer for pancreas lesion diagnostic information modeling, and learning from the real-world feedback, PANDA, which uses only non-contrast CT as input, achieves high sensitivity and exceptionally high specificity in the detection of pancreatic lesions, with a significantly higher accuracy than radiologists in the primary diagnosis between PDAC and non-PDAC, and non-inferior accuracy to radiology reports in the differential diagnosis of the eight aforementioned pancreatic lesion subtypes.

PANDA exhibits effective generalizability to external centers, varying imaging protocols (Extended Data Table 1) and real-world populations. The favorable generalizability of PANDA can be attributed to the following factors. First, the training data are from a high-volume tertiary hospital, encompassing a diverse representation of the Chinese population. Second, non-contrast CT is likely to be a more generalizable modality for AI models than contrast-enhanced CT. Third, our approach combines segmentation (capturing the local pathological basis) and classification, reducing the overfitting risk of pure classification-based AI models. Fourth, the model has been tuned to yield a 99% specificity during cross-validation on the large training set (*n* = 3,208), to achieve reliable control of false positives. Fifth, the AI model’s continual learning^27^ enhances specificity to 99.9% by fine-tuning with false positives from external centers and the real world. And last, regarding training data, the cases and controls have similar CT imaging protocols (for example, slice thickness, CT dose index, oral water), thereby forcing the model to focus on the primary learning objectives rather than fitting to shortcuts or confounders.

PANDA exceeds the performance upper bound of human expert radiologists when reading only in non-contrast CT. This can be attributed to two main reasons. First, during its learning, PANDA is equipped with two informative supervisions that do not exist in non-contrast CT, however, radiologists have not been systematically trained for lesion detection and diagnosis in non-contrast CT. Specifically, one supervision consists of our curated expert lesion annotations transferred from contrast-enhanced CT; the other is the pathology-confirmed lesion types. Second, deep learning algorithms are more sensitive to subtle imaging grayscale intensity changes than human eyes, which are better at using color rather than intensity changes to interpret images^29^. Unlike generative deep learning methods to synthesize contrast or color^18–21^, we train supervised learning models, which effectively capture subtle image details and directly learn downstream lesion detection and diagnosis tasks based on these detailed characteristics. Therefore, PANDA outperforms or matches radiologists on contrast-enhanced CT, the performance of which is in concordance with recent studies^30–32^.

PANDA is an interpretable deep model that outputs the lesion boundaries and lesion subtype probabilities. Although radiologists usually do not diagnose pancreatic lesions from non-contrast CT alone, when assisted by PANDA their performance could be drastically increased regardless of experience, especially for the task of PDAC identification. Radiology residents with less experience benefit the most from PANDA’s assistance, and can reach a level comparable with pancreas specialists. Although general radiologists might still doubt the AI results, their performance could be improved to a level close to that of pancreas specialists. Note that non-contrast CT is widely performed in non-tertiary hospitals and physical examination centers, where radiologists are usually less experienced or not specialized in pancreas imaging diagnosis. In tertiary hospitals, non-contrast CT is commonly performed as well, such as chest CT for lung nodule detection and abdominal CT in the emergency room. Taken together, PANDA could be widely used to increase the level of pancreas cancer diagnosis expertise in medical centers, especially by detecting more pancreatic malignancies at an earlier stage.

To assess the added value of PANDA for real-world clinical misdetection, we used the stricter standard of care clinical diagnosis as the standard of truth, which accounted for the entire patient management scenario, beyond the radiology report alone. Even so, of the 20,530 consecutive patients evaluated retrospectively, PANDA detected five cancers and 26 other pancreatic lesions that were missed by the initial standard of care, and enabled curative treatment of one patient with PNET.

Despite its high mortality rate, PDAC is relatively uncommon. Screening for PDAC in the asymptomatic population was not recommended because existing diagnostic methods would lead to a large number of false positives, resulting in considerable ramifications and costs. Although AI advancement in the areas of pancreatic lesion detection and diagnosis has occurred with the use of contrast-enhanced CT and EUS^30,33,34^, the level of specificity is insufficient, and applying these imaging techniques to the general population is impractical due to their invasiveness, cost, and the need for iodine contrast. Liquid biopsy for cancer detection^26,35,36^ has shown specificities of more than 99% but the sensitivity for early-stage pancreatic cancer detection is only satisfactory (approx. 50–60%, refs. ^35,36^). PANDA Plus (hereinafter referred to as PANDA) was highly sensitive (>96%) for early-stage PDAC and yielded an exceptional specificity of 99.9% in the large-scale real-world evaluation, which equates to approximately one false positive out of 1,000 tests. On the one hand, such a performance enables opportunistic screening in asymptomatic populations. Considering the prevalence of PDAC (13 cases per 100,000 adults), the PPV for PDAC identification will be approximately 10% (11 true positives and 100 false positives in 100,000 tests). This is even higher than the PPVs of some other cancer screening tests currently recommended by the Preventive Services Task Force (USPSTF), for example, mammography for breast cancer, with a PPV of 4.4% (ref. ^37^), stool DNA for colorectal cancer, with a PPV of 3.7% (ref. ^38^), and low-dose CT for lung cancer, with a PPV of 3.8% (ref. ^39^). Our experiments also show that when PANDA was applied in routine multi-scenario CT examinations, PDAC detection in asymptomatic adults could potentially be considered at no additional cost, with no extra examination or radiation exposure. Ideally, even if 10 AI-identified patients with PDAC underwent follow-up exams to confirm one PDAC at a 10% PPV, the overall cost per PDAC found remains manageable. For example, the price ranges from US$1,264 to US$1,685 in Shanghai, China, for 10 exams, depending on the specific type of follow-up exam, that is, contrast-enhanced CT, MRI or EUS, although the price could be higher in Western countries. Nevertheless, further prospective studies are needed to assess the risk–benefit ratio and cost-effectiveness in the future. On the other hand, PANDA could also be used in designed screening in high-risk populations^40^ (Supplementary Methods 1.4). In such a scenario, the sensitivity of (particularly early-stage) PDAC identification can be further improved by adjusting the model threshold at the cost of a slight decrease in specificity. In both opportunistic and designed screening scenarios, PANDA is meant to be used in screening, a pre-step before diagnosis, and not to replace existing diagnostic imaging modalities. Nevertheless, PANDA’s reliable initial diagnosis can better assist physicians in triaging and managing patients with pancreatic lesions, a frequent dilemma in clinical practice^41^.

PANDA is trained on a continual learning approach using multicenter data, but includes only limited data outside the East Asian population and hospitals. The model should be further validated in external real-world centers, more international cohorts, and prospective studies. PANDA exhibited relatively low accuracy for PNET. PNET tumors are rare and highly diverse in appearance, and the model may primarily miss some cases with very low image contrast in non-contrast CT.

PANDA has already demonstrated its potential for accurate detection of other cancers, especially cancer types (esophagus^42^, liver^43^, stomach^44^) for which no guideline-recommended screening tests are available for average-risk individuals. This opens up an exciting possibility of universal cancer detection at both high sensitivity and high specificity levels, while requiring only a non-invasive, low-cost, widely adopted non-contrast CT scanning procedure. We hope that PANDA and its variations will help transform the current cancer-detection paradigm from late-stage diagnosis, when symptoms first present, to early-stage screening in which cancers can be detected before symptoms appear.

## Methods

### Ethics approval

The retrospective collection of the patient datasets in each cohort was approved by the institutional review board (IRB) at each institution with a waiver for informed consent: the Shanghai Institution of Pancreatic Diseases (SIPD) IRB, Shengjing Hospital of China Medical University (SHCMU) IRB, First Affiliated Hospital of Zhejiang University (FAHZU) IRB, Xinhua Hospital (XH) of Shanghai Jiao Tong University School of Medicine IRB, Fudan University Shanghai Cancer Center (FUSCC) IRB, Tianjin Medical University Cancer Institute and Hospital (TMUCIH) IRB, Sun Yat-Sen University Cancer Center (SYUCC) IRB, Guangdong Provincial People’s Hospital (GPPH) IRB, Linkou Chang Gung Memorial Hospital (CGMH) IRB, and General University Hospital in Prague (GUHP) IRB. All data in this study were de-identified prior to model training, testing and reader studies.

### Dataset description

This multicenter retrospective study involved five patient cohorts: an internal training cohort, on which the AI models were built; an internal test cohort, on which the model performance and reader study were assessed (together with an additional internal differential diagnosis cohort to increase statistical power for the evaluation of the model’s performance on differential diagnosis); an external multicenter (*n* = 9) test cohort, on which the generalization across multiple centers was assessed; a chest non-contrast CT test cohort, on which the generalization to chest CT scans was assessed; and a real-world clinical evaluation cohort, on which critical questions about the clinical translation were assessed.

PDAC and seven non-PDAC lesion subtypes (PNET, SPT, IPMN, MCN, SCN, chronic pancreatitis and ‘other’)^33,41,45^ were targeted in this study. In the first four cohorts, PDAC and non-PDAC lesions were confirmed by surgical or biopsy histopathology. The patient-level label of the surgical pathology was determined based on the 2019 *World Health Organization Classification of Tumors - 5th edition, Digestive System Tumors*. For biopsy pathology, definitive evidence was required for diagnosis. Patients with mixed neoplasms were not included. The normal controls were confirmed as being free of pancreatic or peri-pancreatic disease at 2 year follow-up (details of the collection process are given in Supplementary Methods 1.1.1). Patients with acute pancreatitis and a history of abdominal treatment were excluded. In the real-world cohort, pathology or the standard of care clinical diagnosis was used as the ground truth. All of the patients in the five cohorts were staged according to the eighth edition of the AJCC (American Joint Committee on Cancer) cancer staging system. The characteristics of the study participants are listed in Extended Data Table 1 (patient and CT characteristics), Supplementary Table 2 (reference standard of lesion types) and Supplementary Table 3 (lesion size stratified by lesion type). More details of the datasets included in this study are given below and in Supplementary Methods 1.1.2–1.1.7.

#### Internal training cohort

The internal training cohort consisted of 3,208 patients (1,431 with PDAC, 140 with PNET, 98 with SPT, 254 with IPMN (163 with main/mixed-duct IPMN and 91 with branch-duct IPMN), 37 with MCN, 110 with chronic pancreatitis, 134 with SCN, 66 with ‘other’ (Supplementary Table 1) and 938 normal controls) who had been treated between January 2015 and October 2020 at the SIPD, China. Consecutive patients (except for those who had chest CT before surgery, refer to the ‘Chest computed tomography test cohort’ section) with pancreatic lesions confirmed on surgical pathology were included.

#### Lesion and pancreas annotation

Besides the patient-level label, we also annotated pixel-level segmentation masks of the lesion and pancreas. We required only manual annotation of the lesion masks. Due to the difficulty of, and issues with reliability regarding, direct lesion annotation by radiologists using only non-contrast images, we additionally collected paired contrast-enhanced CT scans for annotation purposes. Pancreatic lesion annotations on non-contrast CT images were obtained by image registration from an experienced radiologist’s manual annotations on the contrast-enhanced CT phase images, where tumors were more visible. The pancreas annotations were obtained via an improved version of our annotation-efficient semi-supervised learning approach^46^, which uses only publicly available pancreas annotations (Supplementary Methods 1.1.3).

#### Internal test and differential diagnosis cohorts

We used the testing set of our prior work^47^ as the source of the internal test cohort of the current study, given that interpretations on this set by 11 readers had been collected. Furthermore, we excluded ampullary and common bile duct cancer cases because they were usually not categorized as pancreatic lesions in the literature^41,45^. In addition, one normal participant was re-categorized as having chronic pancreatitis (actually autoimmune pancreatitis, but treated as chronic pancreatitis in our study) after carefully checking the patient records; and one normal participant was excluded due to a severe pancreatic duct dilation. As a result, the internal test cohort contained CT scans of 291 patients randomly collected between December 2015 and June 2018 at the SIPD, China, consisting of 108 with PDAC, 9 with SPT, 5 with PNET, 22 with IPMN (11 with main or mixed-duct IPMN and 11 with branch-duct IPMN), 2 with MCN, 10 with SCN, 13 with chronic pancreatitis, 6 with ‘other’, and 116 normal controls.

To enhance the statistical power of the differential diagnosis evaluation, we also collected an internal addition cohort consisting of 611 consecutive patients who underwent surgery between November 2020 and October 2021 at SIPD (367 with PDAC, 53 with PNET, 30 with SPT, 65 with IPMN (40 with main or mixed-duct IPMN and 25 with branch-duct IPMN), 21 with MCN, 32 with chronic pancreatitis, 19 with SCN, and 24 with ‘other’). These 611 patients, and the 175 patients with pancreatic lesions in the internal test cohort, constitute the internal differential diagnosis cohort (*n* = 786). All patients underwent multi-phase CT, including non-contrast, arterial, venous, and delay. We used only the non-contrast phase for PANDA testing and the first reader study. The multi-phase CT scans of the internal test cohort were used for the second reader study.

#### External multicenter test cohorts

The external test cohorts were collected from nine centers, of which seven were located in China, one in Taiwan ROC (CGMH, Site H), and one in the Czech Republic (GUHP, Site I). The seven centers from China are distributed widely in geographical area: one in the northeast (SHCMU, Site A), four in the east (FAHZU, Site B; XH, Site C; FUSCC, Site D; TMUCIH, Site E), and two in the south (SYUCC, Site F; GPPH, Site G). Inclusion criteria were as follows: non-contrast abdominal CT fully covering the pancreas region before treatment; ground truth lesion type confirmed on either surgical or biopsy pathology; and normal control confirmed on at least 2 years of follow-up. Normal controls in most centers were randomly selected from the same time period as that of lesion collection. Patients with low image quality due to artifacts caused by metal in stents or drastic motion during imaging were excluded. The multicenter test cohort, consisting of non-contrast CT scans of 5,337 patients (2,737 with PDAC, 932 with non-PDAC, and 1,668 normal), was used for independent validation when no model parameters were tuned or adjusted.

#### Chest computed tomography test cohort

To evaluate the model’s generalizability to chest CT, we collected a non-contrast chest CT test cohort with pathology-confirmed PDAC and non-PDAC and normal controls confirmed on 2 year follow-up, from SIPD, which is affiliated with a major tertiary hospital. Specifically, for patients with PDAC or non-PDAC confirmed by surgical pathology, we searched for their nearest chest CT images for up to 1 year before surgery. For patients with chest CT reports of normal pancreas, we searched for their follow-up records of normal pancreas for at least 2 years. By doing so, we collected a cohort of 63 patients with PDAC, 51 with non-PDAC, and 378 normal controls spanning from November 2015 to May 2022 at SIPD. These non-contrast chest CT scans of PDAC and non-PDAC were acquired 4 days (range, −20 to 191 days) before the contrast-enhanced abdominal CT diagnosis, and most of them were acquired during the COVID-19 pandemic for prevention purposes in this tertiary hospital. We ensured that all patients were independent of the patients in the training cohort.

#### Real-world evaluation cohorts

The real-world, retrospective studies consisted of two rounds (RW1 and RW2) of evaluations between July 2022 and October 2022 at the SIPD. The clinical trial was complete and registered with http://www.chictr.org.cn, ChiCTR2200064645, and included both RW1 and RW2. PANDA was evaluated on RW1, and PANDA Plus (that is, the upgrade of PANDA by learning from the internal, external and RW1 feedback) was evaluated on RW2. The inclusion criterion was the availability of a non-contrast CT scan covering the pancreas region, for example, lung, esophagus, liver or kidney CT. Patients with acute pancreatitis (in RW1), abdominal cancer treatment, severe ascites, abdominal trauma, and low imaging quality were excluded. The process of the standard of truth determination is described in Extended Data Figs. 5 and 6.

Our real-world data were collected from four scenarios, that is, physical examination, emergency, inpatient, and outpatient department (Supplementary Methods 1.1.7). Because the patient indications, the CT image background complexity, the pancreatic lesion prevalence, and the experience of the (first-line) radiologists varied widely between these four scenarios, we conducted separate evaluations to determine the feasibility of opportunistic screening using PANDA. These results can serve as a valuable reference when applied to different countries or institutions based on the sources of patients.

The original RW1 consisted of 18,654 consecutive individuals whose non-contrast CT scans were examined between 1 and 31 December 2021, from four different clinical scenarios at the SIPD. After exclusion (*n* = 2,234, 12%), 16,420 individuals remained (that is, 9,429, 3,027, 2,311 and 1,653 from the physical examination, emergency, outpatient and inpatient scenarios, respectively). RW1 included 44 PDACs, 6 PNETs, 1 SPT, 15 IPMNs, 1 MCN, 42 cases of chronic pancreatitis, 11 SCNs and 59 cases of ‘other’ (mostly benign cysts).

The original RW2 consisted of 4,815 consecutive individuals between 1 and 10 February 2022, from the four clinical scenarios at the SIPD. The exclusion criteria were the same as for RW1, except that we included acute pancreatitis for RW2. After exclusion (*n* = 705, 15%), 4,110 individuals remained (1,854, 969, 688 and 599 from the physical examination, emergency, outpatient, and inpatient scenarios, respectively). RW2 included 32 PDACs, 5 PNETs, 1 SPT, 12 IPMNs, 4 MCNs, 55 cases of chronic pancreatitis, 2 SCNs, 15 cases of ‘other’, and 40 cases of acute pancreatitis.

### AI model: PANDA

PANDA consists of three stages (Extended Data Fig. 1) and was trained by supervised machine learning. Given the input of a non-contrast CT scan, we first localize the pancreas, then detect possible lesions (PDAC or non-PDAC), and finally classify the subtype of the detected lesion if any. The output of PANDA consists of two components, that is, the segmentation mask of the pancreas and the potential lesion, and the classification of the potential lesion associated with probabilities of each class.

#### Pancreas localization

The aim of the first stage (Stage 1) is to localize the pancreas. Because the pancreatic lesion is usually a small region in the CT scan, the localization of the pancreas can accelerate the lesion finding process and prune out unrelated information for the specialized training of the pancreatic region. In this stage we train an nnU-Net^23^ to segment the whole pancreas (the union mask of healthy pancreas tissue and the potential lesions) from the input non-contrast CT scan. Specifically, the three-dimensional (3D) low-resolution nnU-Net, which trains UNet on downsampled images, is used as the architecture because of its efficiency in inference. The model training is supervised by the voxel-wise annotated masks of the pancreas and lesion. More details on the training and inference for PANDA Stage 1 are given in Supplementary Methods 1.2.1.

#### Lesion detection

The aim of the second stage (Stage 2) is to detect the lesion (PDAC or non-PDAC). We trained a joint segmentation and classification network to simultaneously segment the pancreas and potential lesion, as well as classify the patient-level abnormality label, that is, abnormal or normal. The benefit of the classification branch is to enforce global-level supervision and produce a patient-level probability score, which is absent in semantic segmentation models. Similar designs had been used in previous studies, such as for cancer detection^47,48^ and outcome prediction^49^. The network architecture is shown in Extended Data Fig. 1b. This is a joint segmentation and classification network with a full-resolution nnU-Net^23^ backbone (left part in Extended Data Fig. 1b). We extract five levels of deep network features, apply global max-pooling, and concatenate the features before carrying out the final classification. We output both the segmentation mask of the potential lesion and pancreas, and the probabilities of abnormal or normal for enhanced interpretability. This network was supervised by a combination of segmentation loss and classification loss:

1 $${\mathcal L} ={ {\mathcal L} }_{{\rm{seg}}}+\alpha { {\mathcal L} }_{{\rm{cls}}}$$

where the segmentation loss ${ {\mathcal L} }_{{\mathrm{seg}}}$ was an even mixture of Dice loss and voxel-wise cross-entropy loss, and the classification loss was the cross-entropy loss. *α* was set to 0.3 to balance the contribution of the two loss functions. More details on the training and inference of PANDA Stage 2 are given in Supplementary Methods 1.2.2.

#### Differential diagnosis

The aim of the third stage network (Stage 3) is the differential diagnosis of pancreatic lesion type, which is formulated as the classification of eight sub-classes, that is, PDAC, PNET, SPT, IPMN, MCN, chronic pancreatitis, SCN and ‘other’. Due to the subtle texture change in pancreatic diseases, especially on non-contrast CT scans, we incorporate a separate memory path network that interacts with the UNet path to enhance the ability to model global contextual information, which is usually associated with the diagnosis of pancreatic lesions by radiologists. As shown in Extended Data Fig. 1c, we use a dual-path memory transformer network. This design is inspired by Max-Deeplab^25^. The architecture of the UNet branch is the same as that of Stage 2, implemented as a full-resolution nnU-Net. The UNet branch takes the input of the cropped 3D pancreas bounding box, which is cropped with a fixed input size of (160, 256, 40). The memory branch starts with learnable memories designed to store both positional and texture-related prototypes of the eight types of pancreatic lesion, and is initialized as 200 tokens with 320 channels. The memory path iteratively interacts with multi-level UNet features (plus a shared learnable positional embedding across layers) via cross-attention and self-attention layers. Through this process the memory vectors were automatically updated to encode both the texture-related information from the UNet features and the positional information from the learnable positional embedding, for example, relative positions of the pancreatic lesion inside the pancreas, resulting in distinguishable descriptors for each type of pancreatic lesion.

The mechanism of the cross-attention and self-attention used in the model is formally described in Supplementary Methods 1.2.3, together with more details on model instantiation, training and inference of PANDA Stage 3.

Additionally, we trained an IPMN subtype classifier in a cascaded fashion following PANDA Stage 3, with the aim of binary classification between main or mixed-duct IPMN and branch-duct IPMN (Supplementary Methods 1.2.3).

#### Generalization of PANDA to chest computed tomography

One major difference between chest CT and abdominal CT is that the pancreatic and lesion regions are sometimes partially scanned in chest CT, depending on the different scanning ranges of the protocol and the anatomy of the patient. This difference could induce domain shift issues for machine learning models if our AI model was trained only on abdominal CT scans. To address this issue we propose a data augmentation method that randomly (with a probability) cuts off the pancreas region in the axial plane to simulate the imaging scenario in which the pancreas is not fully scanned in the chest CT. This data augmentation is applied to the training process of Stages 2 and 3. This simple simulation of the chest CT effectively helps our model generalize to chest non-contrast CT without the addition of any chest CT data to the training set, while maintaining high performance on abdominal non-contrast CT.

#### Real-world deployment and model evolution

In the real-world clinical evaluation, PANDA was deployed at SIPD by integrating it into the clinical infrastructure and workflow (Supplementary Fig. 9). The deployment facilitates large-scale retrospective real-world studies in the hospital environment by securing data privacy, efficiently utilizing computational resources, and accelerating the process of large data inference and clinical evaluation. Specifically, we deploy PANDA in a local server located in the hospital (Supplementary Methods 1.2.4), which enables radiologists to visualize each case using our user-friendly DAMO Intelligent Medical Imaging user interface (IMI UI; Supplementary Fig. 9), easily review all results and access necessary information from their daily work environment. After RW1 we again collected non-contrast CT data of false positives and negatives and cases of acute pancreatitis from the internal, external and RW1 cohorts. In the field of machine learning this is known as hard example mining and incremental learning. The evolved model was named PANDA Plus and tested on RW2. The collection and annotation of these new training data and the fine-tuning schedule are described in Supplementary Methods 1.2.5.

### Evaluation metrics

#### Lesion detection metrics

Lesion detection is a binary classification task to distinguish whether the patient has a pancreatic lesion or not. Having a lesion is defined as the ‘positive’ class for calculation of the AUC, sensitivity, specificity, accuracy and balanced accuracy. In addition, we evaluate the lesion detection rates stratified by lesion type. Particularly for the PDAC cases, we assess the sensitivity for detection stratified by cancer stage (stages I–IV) and tumor stage (T1–4).

#### Primary diagnosis metrics

Primary diagnosis is a three-class classification task to distinguish PDAC versus non-PDAC versus normal. We use the top-1 accuracy and three-class balanced accuracy to present the detailed results of the three-class classification. In addition, we define a PDAC identification task because PDAC is a unique lesion type with the most dismal prognosis. Distinguishing it from other types, that is, PDAC versus non-PDAC + normal, is always the primary question to answer for doctors and is the key task for cancer screening. Having a PDAC is defined as the ‘positive’ class for calculation of the AUC, sensitivity, specificity, PPV, accuracy and balanced accuracy.

#### Differential diagnosis metrics

Differential diagnosis is an eight-class classification task for the seven most common pancreatic lesion types and ‘other’, following the pancreatic tumor–cyst classification task^41,45^, without normal patients included and with each patient having a lesion type assigned. The confusion matrices are used to present the detailed classification results. We report the overall top-1 accuracy and multi-class balanced accuracy for the classification of all of the lesion types, to facilitate the comparison of the AI model’s performance with second-reader radiology reports and across external multiple centers. The second-reader radiology report is a secondary analysis of a primary standard of care clinical radiology report, in which radiologists have complete access to the patient’s clinical history (for example, contrast-enhanced CT examination indicated for chronic pancreatitis follow-up), and the results of other clinical examinations (for example, tumor biomarkers). In addition, we also report the performance of the full pipeline (lesion detection + differential diagnosis), that is, nine-class classification consisting of normal and eight lesion types.

### Ablation studies

We perform three ablation studies. For PANDA Stage 2 we compare our multi-task CNN model with a volume-based classifier on the nnU-Net segmentation model (Extended Data Fig. 2a). This baseline model uses the volume of the segmented lesion by an nnU-Net as an indicator for the existence of the lesion. For PANDA Stage 3 we compare our dual-path transformer model with the Stage 2 multi-task CNN model. In addition, we demonstrate the importance of the quantity of training data on different tasks of our problem (Extended Data Fig. 3). We first retrain the PANDA model under four settings, using 10%, 25%, 50% and 75% of the training dataset, respectively, and then test the model in each setting on the internal and external test cohorts.

### Reader studies

Two groups of readers participated in two independent reader studies.

#### Reader study on non-contrast computed tomography

The aim of the first reader study was to assess the readers’ performance in detecting pancreatic lesions and diagnosing whether the lesion was a PDAC on non-contrast CT. The study was conducted in two sessions. The first session compared PANDA’s performance with that of radiologists with varying levels of expertise in pancreatic imaging. The second session investigated whether PANDA would be capable of assisting radiologists. There was a washout period of at least 1 month between the two rounds for each reader.

A total of 33 readers from 12 institutions were recruited in this study, consisting of 11 pancreatic imaging specialists, 11 general radiologists who are not specialized in pancreatic imaging, and 11 radiology residents. These readers had practiced for an average of 8.3 years (range, 2–31 years) in various radiology departments, and had read an average of 510 pancreatic CT scans (range, 100–2,600) in the year before the reader study (Extended Data Table 2).

In the first session each reader was trained to use the ITK-SNAP software^50^ for the visualization of the CT images. Basic functions of this software include but are not limited to HU (Hounsfield unit) windowing, zooming in and out, and axial, sagittal and coronal view simultaneous display. In interpreting the 291 randomly ordered cases from the internal test cohort, non-contrast CT images and information on age and sex were provided. The readers were informed that the study dataset was enriched with more positive patients than the standard prevalence of pancreatic lesions in daily practice. However, they were not informed about the proportions of each class. Each reader interpreted the image without time constraints and classified each case as PDAC, non-PDAC or normal. In addition to the patient-level label, each reader also recorded the location of the detected tumor in the format of pancreatic head/uncinate, neck, and body/tail. The performance of each reader is listed in Supplementary Tables 6 and 8.

In the second session the same group of readers interpreted the 291 cases again using ITK-SNAP. In addition to the non-contrast CT images and the information on age and sex, the readers were provided with PANDA’s case-level prediction probability of PDAC, non-PDAC or normal, as well as the corresponding lesion segmentation masks. Some examples of the provided PANDA predictions (in interactive video format) are shown in Supplementary Fig. 3. The improvement of each reader between the two sessions is measured.

#### Reader study on contrast-enhanced computed tomography

The second reader study compared PANDA’s (non-contrast CT) performance with that of pancreatic imaging specialists' readings on contrast-enhanced CT. A total of 15 additional pancreatic imaging specialists from a high-volume pancreatic cancer institution (SIPD) were recruited in this study. These readers had practiced for an average of 9.5 years (range, 6–19 years) in the radiology department at SIPD, and had read an average of 907 pancreatic CT scans (range, 400–3,000) in the year prior to the reader study (Extended Data Table 2).

Each reader was first trained to use the same software for visualizing multi-phase CT images. Next, they were provided with the non-contrast, arterial and venous phase CT images of the same 291 patients from the internal test cohort, as well as information on age and sex. The interpretation rules were the same as those of the first reader study. We also measured individual differences between non-contrast CT and contrast-enhanced CT (Supplementary Methods 1.3.1).

### Interpretability of the AI model

Our AI model jointly outputs the probability of the abnormality, the prediction of the subtype classification (if any abnormality is detected), and the segmentation mask of the detected abnormality lesion. Unlike other AI-based classification models^51,52^ that require the visualization of the network feature map to acquire the abnormality’s positional cues, our model directly outputs the segmentation mask of the detected mass together with the patient-level probability, which provides straightforward and advanced interpretability. The correspondence between the segmented lesion and the ground truth lesion was evaluated using the Dice coefficient (DSC) and the 95th percentile of Hausdorf distance (HD95). The segmentation performance of the pancreas and each type of pancreatic lesion was evaluated.

In addition, we visualized the heatmap of the convolutional feature map of PANDA Stage 2 classification using Grad-CAM^53^ (Extended Data Fig. 4a), to understand which part of the feature map contributed most to lesion detection. For PANDA Stage 3 lesion differential diagnosis, we plotted the attention map of the memory tokens, which showed the activation of the top activated tokens (Extended Data Fig. 4b) to interpret the model’s attention.

### Statistical analysis

The performance of the binary classification task was evaluated using the AUC, sensitivity, specificity, PPV, accuracy and balanced accuracy metrics. The performance of the multi-class classification task was evaluated using accuracy and balanced accuracy. Cohen’s kappa coefficient *κ* was also computed between the AI prediction and the standard of truth for differential diagnosis. The confidence intervals were calculated based on 1,000 bootstrap replications of the data. The significance comparisons of sensitivity, specificity, accuracy and balanced accuracy were conducted using permutation tests to calculate two-sided *P* values with 10,000 permutations. For non-inferiority comparisons, a 5% absolute margin was pre-specified before the test set was inspected. The significance of the difference between the AUCs of the AI model and nnU-Net was assessed using the Delong test. The threshold to determine statistical significance is *P* < 0.05. Data analysis was conducted in Python using the numpy (v1.20.3), scipy (v1.8.1) and scikit-learn (v0.24.2) packages.

### Reporting summary

Further information on research design is available in the Nature Portfolio Reporting Summary linked to this article.

## Online content

Any methods, additional references, Nature Portfolio reporting summaries, source data, extended data, supplementary information, acknowledgements, peer review information; details of author contributions and competing interests; and statements of data and code availability are available at 10.1038/s41591-023-02640-w.

### Supplementary information

Supplementary Information Supplementary Methods, Supplementary Figs. 1–20 and Supplementary Tables 1–14.

Reporting Summary

## Figures and Tables

**Extended Data Table 1:**
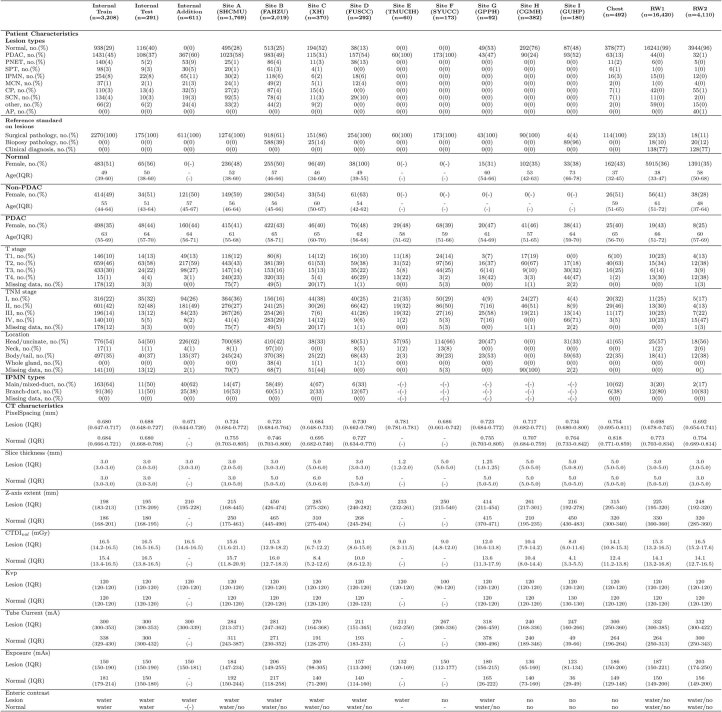
Dataset characteristics

T stage (AJCC eighth edition), TNM stage (AJCC eighth edition), and location are displayed for the PDAC patients. no., number; IQR, interquartile range; Internal Addition, additional cohort to enhance the evaluation of differential diagnosis, together with the 175 patients with pancreatic lesions in the internal test cohort, constitute the internal differential diagnosis cohort (n=786); SHCMU, Shengjing Hospital of China Medical University; FAHZU, First Affiliated Hospital of Zhejiang University; XH, Xinhua Hospital; FUSCC, Fudan University Shanghai Cancer Center; TMUCIH, Tianjin Medical University Cancer Institute and Hospital; SYUCC, Sun Yat-Sen University Cancer Center; GPPH, Guangdong Provincial People’s Hospital; CGMH, Chang Gung Memorial Hospital; GUHP, General University Hospital in Prague; RW, real-world cohort.

**Extended Data Table 2:**
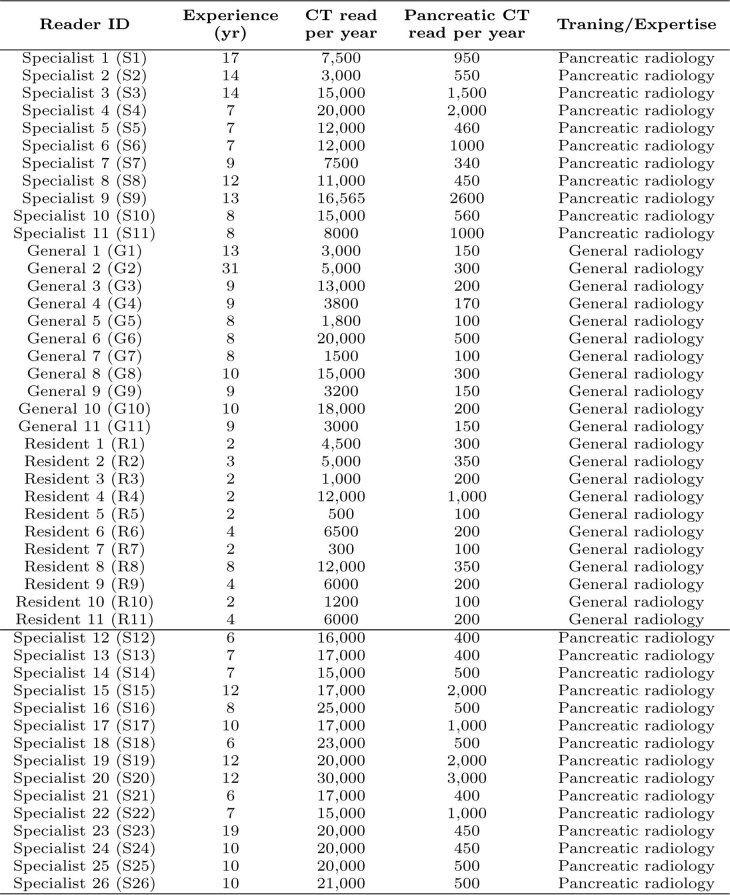
Reader experience

Specialists were radiologists who had ≥5 years of experience in pancreatic imaging. Specialists 4 and 20 were highly regarded for their excellence within a high-volume pancreatic cancer institution. General 1 -General 11 were general radiologists who were practicing at community hospitals or other level hospitals and undergoing a refresher program in pancreatic radiology at the SIPD center at the time of the reader study. Resident 2 was a radiology resident whose research interest was pancreatic imaging.

**Extended Data Table 3:**
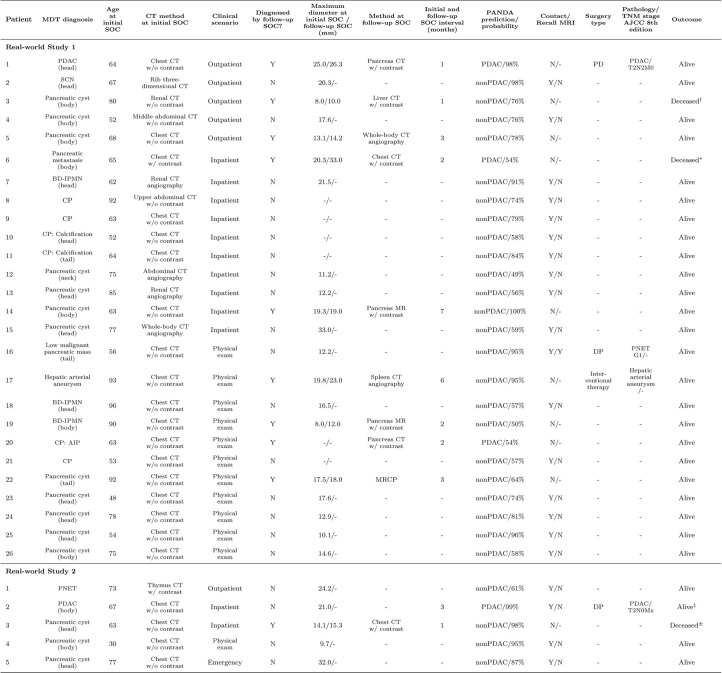
Cases that were misdetected by the initial standard of care (SOC) but were successfully detected by PANDA in the real-world clinical evaluations

The last outcome follow-up is in February 2023. *Died due to metastases originating from lung cancer. †Died due to lung cancer. ‡Underwent surgery in another hospital. ± Died due to lung cancer. MDT, multi-disciplinary team; AJCC, American Joint Committee on Cancer; PDAC, pancreatic ductal adenocarcinoma; BD-IPMN, branch duct intraductal papillary mucinous neoplasm; AIP, autoimmune pancreatitis; CP: Chronic pancreatitis; MCN, mucinous cystic neoplasm; PNET, pancreatic neuroendocrine tumor; MRCP, magnetic resonance cholangiopancreatography; DP, distal pancreatectomy; PD, pancreatoduodenectomy; Y, yes; N, no.

## Data Availability

Sample data and an interactive demonstration are given at http://panda.medofmind.com/. The remaining datasets used in this study are currently not permitted for public release by the respective institutional review boards. Requests for access to aggregate data and supporting clinical documents will be reviewed and approved by an independent review panel on the basis of scientific merit. All data provided are anonymized to protect the privacy of the patients who participated in the studies, in line with applicable laws and regulations. Data requests pertaining to the study may be made to the first author (Kai Cao; mdkaicao163@163.com). Requests will be processed within 6 weeks.

## Extended data

are available for this paper at 10.1038/s41591-023-02640-w.

## Code availability

The code used for the implementation of PANDA has dependencies on internal tooling and infrastructure, is under patent protection (application numbers: CN 202210575258.9, US 18046405), and thus is not able to be publicly released. All experiments and implementation details are described in sufficient detail in the Methods and Supplementary Information (Methods) sections to support replication with non-proprietary libraries. Several major components of our work are available in open-source repositories: PyTorch (https://pytorch.org/) and nnU-Net (https://github.com/MIC-DKFZ/nnUNet).
