# Supplementary material for: Large-scale pancreatic cancer detection via non-contrast CT and deep learning
Source: Nat Med. 2023 Nov 20;29(12):3033–43. doi: 10.1038/s41591-023-02640-w (PMC10719100; doi:10.1038/s41591-023-02640-w)
Supplement: Supplementary file 1 — Supplementary Methods, Supplementary Figs. 1–20 and Supplementary Tables 1–14. [file 41591_2023_2640_MOESM1_ESM.pdf]

nature medicine

Article https://doi.org/10.1038/s41591-023-02640-w

Large-scale pancreatic cancer detection via non-contrast CT and deep learning

In the format provided by the authors and unedited

Supplementary Information: Large-Scale Pancreatic Cancer Detection via Noncontrast CT and Deep Learning

Kai Cao[1†], Yingda Xia[2†], Jiawen Yao[2,3†], Xu Han[4†], Lukas Lambert[5†], Tingting Zhang[6†], Wei Tang[7†], Gang Jin[8], Hui Jiang[9], Xu Fang[1], Isabella Nogues[10], Xuezhou Li[1], Wenchao Guo[2,3], Yu Wang[2,3], Wei Fang[2,3], Mingyan Qiu[2,3], Yang Hou[11], Tomas Kovarnik[12], Michal Vocka[13], Yimei Lu[7], Yingli Chen[8], Xin Chen[14], Zaiyi Liu[14], Jian Zhou[15], Chuanmiao Xie[15], Rong Zhang[15], Hong Lu[16], Gregory D. Hager[17], Alan L. Yuille[17], Le Lu[2], Chengwei Shao[1*], Yu Shi[11*], Qi Zhang[4*], Tingbo Liang[4*], Ling Zhang[2*] and Jianping Lu[1*]

[1]Department of Radiology, Shanghai Institution of Pancreatic Diseases, China.

[2]DAMO Academy, Alibaba Group.

[3]Hupan Lab, Hangzhou, China.

[4]Department of Hepatobiliary and Pancreatic Surgery, the First Affiliated Hospital of Zhejiang University, China.

[5]Department of Radiology, First Faculty of Medicine, Charles University and General University Hospital in Prague, Czech Republic.

[6]Department of Radiology, Xinhua Hospital, Shanghai Jiao Tong University School of Medicine, China.

[7]Department of Radiology, Fudan University Shanghai Cancer Center, China.

[8]Department of Surgery, Shanghai Institution of Pancreatic Diseases, China.

[9]Department of Pathology, Shanghai Institution of Pancreatic Diseases, China.

[10]Departments of Biostatistics, Harvard University T.H. Chan School of Public Health, USA.

[11]Department of Radiology, Shengjing Hospital of China Medical University, China.
[12]Department of Invasive Cardiology, First Faculty of Medicine, Charles University and General University Hospital in Prague, Czech Republic.
[13]Department of Oncology, First Faculty of Medicine, Charles University and General University Hospital in Prague, Czech Republic.
[14]Department of Radiology, Guangdong Provincial People's Hospital, China.
[15]Department of Radiology, Sun Yat-sen University Cancer Center, China.
[16]Department of Radiology, Tianjin Medical University Cancer Institute and Hospital, China.
[17]Department of Computer Science, Johns Hopkins University, USA.

*Corresponding author(s). E-mail(s): cwshao@sina.com; 18940259980@163.com; qi.zhang@zju.edu.cn; liangtingbo@zju.edu.cn; ling.z@alibaba-inc.com; cjr.lujianping@vip.163.com;
†These authors contributed equally to this work.

Contents

1 Supplementary Methods **6**
1.1 Supplementary dataset description 6
1.1.1 Normal control collection 6
1.1.2 Internal training cohort 6
1.1.3 Lesion and pancreas annotation 6
1.1.4 Internal test and differential diagnosis cohorts 7
1.1.5 External multicenter test cohorts 8
1.1.6 Chest CT test cohort 8
1.1.7 Real-world cohorts 9
1.2 PANDA training, inference, deployment, and evolution 10
1.2.1 PANDA Stage-1 10
1.2.2 PANDA Stage-2 10
1.2.3 PANDA Stage-3 10
1.2.4 Real-world deployment 11
1.2.5 PANDA Plus 11
1.3 Reader studies 12
1.3.1 Individual differences between noncontrast and contrast-enhanced CT 12
1.4 Use PANDA for screening in high-risk populations 12

List of Figures

1 Differential diagnosis performance of the second-reader radiology report . . . 14
2 Reader study of 33 readers for primary diagnosis . . . 15
3 Examples of the format of PANDA predictions to the readers . . . 16
4 Reader study of 33 readers with AI assistance for primary diagnosis . . . 17
5 Reader study of 15 readers for primary diagnosis . . . 18
6 Differential diagnosis with PANDA on external test cohorts . . . 19
7 Confusion matrix of the full pipeline . . . 20
8 Examples of PANDA's (peri-)pancreatic disease findings in the real-world clinical evaluation . . . 21
9 Flowchart describing the process of the seamless integration of PANDA into the existing clinical infrastructures . . . 22
10 Flowchart describing the test process of patients of RW1 . . . 23
11 Flowchart describing the test process from the physical exam centers of RW1 . . . 24
12 Flowchart describing the test process from the emergency department of RW1 . . . 25
13 Flowchart describing the test process of outpatients of RW1 . . . 26
14 Flowchart describing the test process of inpatients of RW1 . . . 27
15 Flowchart describing the test process of patients of RW2 . . . 28
16 Flowchart describing the test process from the physical exam centers of RW2 . . . 29
17 Flowchart describing the test process from the emergency department of RW2 . . . 30
18 Flowchart describing the test process of outpatients of RW2 . . . 31
19 Flowchart describing the test process of inpatients of RW2 . . . 32
20 Model evolution . . . 33

List of Tables

1 List of subtypes included in nonPDAC ‘other’ 34
2 Supplementary data characteristics of reference standard of lesion types 35
3 Supplementary data characteristics of lesion size (diameter) stratified by lesion type 36
4 Comparison between PANDA and second-reader radiology report (contrast CT) performance on differential diagnosis . . . 37
5 Segmentation performance of PANDA on the internal test cohort 38
6 Reader (noncontrast CT) vs. PANDA (noncontrast CT) by sensitivity and specificity 39
7 The impact of PANDA assistance on reader performance on noncontrast CT by sensitivity and specificity 40
8 Reader (noncontrast CT) vs. PANDA (noncontrast CT) by accuracy and balanced accuracy 41
9 The impact of PANDA assistance on reader performance on noncontrast CT by accuracy and balanced accuracy 42
10 Reader(contrast-enhanced CT) vs. PANDA (noncontrast CT) by sensitivity and specificity 43
11 Reader (contrast-enhanced CT) vs. PANDA (noncontrast CT) by accuracy and balanced accuracy 44
12 Differential diagnosis results on four external centers with pathologically confirmed PDAC and nonPDAC 44
13 Results for IPMN subtype classification 45
14 Analysis of PANDA’s false positive predictions in real-world evaluation RW1 and RW2 45

1 Supplementary Methods

1.1 Supplementary dataset description

1.1.1 Normal control collection

The patient label of the surgical pathology was determined based on the 2019 WHO classification of tumors (5th edition) of the digestive system. For biopsy pathology, definitive evidence was required for diagnosis. Patients with mixed neoplasms were not included.

We collected normal controls based on the following approach: First, we searched patients whose radiology report of abdominal contrast-enhanced CT had negative pancreatic findings; Then, among these patients, we searched patients who had a record of at least 2-year follow-up and no information in their available clinical diagnosis indicated a pancreatic lesion. Because the centers in our studies are all (or directly affiliated to) tertiary or general academic hospitals, the indication of normal control patients to perform the first CT scan includes various purposes, such as abdominal pain, abnormal blood biomarkers, tumor (other than pancreatic tumor) diagnosis, etc.

1.1.2 Internal training cohort

The pathologist (15 years of specialized experience in pancreatic pathology) and the radiologist (17 years of experience in pancreatic radiology) in our team reviewed the surgical pathology records and contrast-enhanced CT images to determine the ground-truth of IPMN subtypes, i.e., main/mixed-duct or branch-duct IPMN. Among the branch-duct IPMNs, the indication of surgery is one of the following characteristics being observed: growth rate $\geq$5 mm per year; increased levels of serum CA19-9; acute pancreatitis (caused by IPMN); cyst diameter $\geq$50 mm; jaundice (tumor-related). The radiologist in our team reviewed the multi-phase contrast-enhanced CT of the IPMN cases as per revised Fukuoka guidelines for IPMN management [1] and observed 154 cases with high-risk stigmata, 94 cases with worrisome features (if no high-risk stigmata were observed), and 6 cases with neither. Normal controls confirmed by at least 2 years of follow-up were randomly selected from the same time period. All cases had preoperative multi-phase contrast-enhanced CT images acquired by Philips, Siemens, Toshiba/Canon, or Vital scanners.

1.1.3 Lesion and pancreas annotation

Lesion. First, we set the arterial phase as the target phase and registered all the other phases (noncontrast and venous) to the arterial phase using DEEDS [2] image registration algorithm. On the registered images, an experienced radiologist (with 15 years of experience in pancreatic imaging) manually annotated voxel-wise segmentation masks of the pancreatic lesions for all patients on either the arterial or venous phase with better lesion visibility using ITK-SNAP software [3]. During annotation, the radiologist also referred

to all the other CT phases, radiology report, contrast-enhanced MRI, surgical report, and pathology report if necessary. Then, We registered the image (either arterial or venous phase) with radiologist's lesion annotation back to the noncontrast phase using the DEEDS algorithm [2] and applied the registration deformation field to the annotated mask so that we obtained the deformed lesion mask for the original noncontrast image. Our preliminary experiments found that training PANDA on the original noncontrast images with deformed lesion masks had better performance than training on the deformed noncontrast images with original lesion masks. Finally, the deformed lesion masks on the noncontrast images were verified and edited by the radiologist to avoid obvious registration displacements. The radiologists did not directly annotate the tumor on the noncontrast CT because they found it extremely difficult as lesion boundaries are almost invisible and hard to define (especially for PDAC), even when referring to contrast-enhanced CT images. Another benefit of using the registration technique as a starting point is that the size (in volume) of the annotated lesion can be consistent with the one that appeared in the contrast-enhanced CT.

Pancreas. We used our recently developed pancreas segmentation model for three-phase (noncontrast, arterial, and venous) registered CT images [4] to segment the pancreas in the internal training cohort. We empirically found that such a three-phase-based segmentation model was more robust than any single-phase-based model for pancreas segmentation. The model could segment PDAC and nine anatomies, including the pancreas, pancreatic duct, and peripancreatic anatomies (duodenum, aorta, inferior vena cava [IVC], portal vein and splenic vein [PVSV], superior mesenteric vein [SMV], superior mesenteric artery [SMA], and truncus coeliacus [TC]). The segmented masks were post-processed by two steps: (1) change all PDAC masks to pancreas masks, as we had manual PDAC masks; and (2) remove all pancreatic duct masks in the nonPDAC and normal cases, as this yielded higher specificity for lesion detection in preliminary experiments. Then, to obtain the pseudo masks of the pancreas and duct for the original noncontrast phase image, we registered the arterial phase image to the noncontrast phase image in the internal training cohort and applied the registration deformation field to the post-processed masks. The final pancreas mask on the noncontrast image was further verified and edited by an experienced engineer (on the training cohort) or the radiologist (on the internal test cohort) to avoid obvious errors. Finally, the pancreatic lesion manual masks were overlaid on the pancreas and duct masks. The training of our AI model utilized the original noncontrast phase CT images and the voxel-wise masks of the pancreas, pancreatic duct (only in PDAC cases), and lesion.

1.1.4 Internal test and differential diagnosis cohorts

As per revised Fukuoka guidelines [1] for IPMN management, 51 cases had high-risk stigmata, 35 cases had worrisome features if no high-risk stigmata

were observed, and 1 case with neither among the IPMN cases in internal differential diagnosis cohort (n=87).

1.1.5 External multicenter test cohorts

For the IPMNs in the external validation cohorts (n=172), due to the difficulty of retrieving and re-evaluating pathology records/images, the ground-truth of IPMN subtype was based on radiology evaluation by a radiologist in our team (17 years of specialized experience in diagnostic pancreatic imaging) by reviewing the multi-phase contrast-enhanced CT images [5]), which led to 82 main/mixed-duct IPMN and 90 branch-duct IPMN. As per revised Fukuoka guidelines [1], 101 cases had high-risk stigmata, 70 cases had worrisome features if no high-risk stigmata were observed, and 1 case with neither.

Site A. SHCMU, is a tertiary hospital in China. We consecutively collected 1,023 patients with PDAC and 251 patients with nonPDAC, and randomly selected 495 normal controls from January 2010 to May 2020.

Site B. FAHZU, is a tertiary hospital in China. We consecutively collected 983 patients with PDAC and 523 patients with nonPDAC from May 2020 to July 2022, and randomly collected 513 normal controls from Dec 1 2021 to Dec 31 2021.

Site C. XH, is a tertiary hospital in China. We consecutively collected 115 patients with PDAC and 61 patients with nonPDAC, and randomly selected 194 normal controls from January 2019 to December 2020.

Site D. FUSCC, is a tertiary hospital in China. We collected 157 PDAC, 97 nonPDAC, and 38 normal controls from November 2016 to November 2020.

Site E. TMUCIH, is a tertiary hospital in China. We collected 60 patients with PDAC from January 2010 and November 2019.

Site F. SYUCC, is a tertiary hospital in China. We consecutively collected 173 patients with PDAC from March 2010 to April 2020.

Site G. GPPH, is a tertiary hospital in China. We collected 43 patients with PDAC and randomly selected 49 normal controls from January 2011 and August 2015.

Site H. CGMH, is a hospital in Taiwan, ROC. Doctors from CGMH consecutively collected 90 patients with PDAC and randomly selected 292 normal controls from March 2009 to November 2015. We deployed and run PANDA using the NVIDIA-docker container (version 2.0, https://github.com/NVIDIA/nvidia-docker) in a local workstation in CGMH.

Site I. GUHP, is a hospital in the Czech Republic. We consecutively collected 93 patients with PDAC and randomly selected 87 normal controls from August 2005 to March 2022.

1.1.6 Chest CT test cohort

Chest CT scans are obtained with patients in the supine position and with the thorax centered within the gantry and both arms elevated. The respiration phase is inspiration via a single breath-hold. The scan extent ranges from the

lung species to the bottom. Radiology technicians need to perform a CT localizer scan before determining the scanning range. Usually, relying solely on a single position's localizer image, such as the anterior-posterior view, may not accurately determine the scanning position of the lower lung border. Therefore, in our routine chest CT scanning protocol, a lateral view is commonly added better to determine the scanning position of the lower lung border. This often results in the scanning range of the chest CT covering a portion of the abdominal organs, such as the liver, spleen, kidneys, and pancreas. The X-ray tube voltage is 120kV, and the tube current ranges from 137–380 mA. Each case has a field of view (FOV) ranging from 300mm to 418mm, slice thickness of 1mm and 5mm, and is reconstructed using both lung and soft-tissue kernels. In this study, PANDA was run on the chest CT scans with a slice thickness of 5mm reconstructed via soft-tissue kernel.

1.1.7 Real-world cohorts

The standard of truth of all patients was determined as follows. First, two radiologists analyzed electronic medical records (EMRs) to determine the SOC clinical diagnosis. Then, unspecified cases were determined by a multidisciplinary team (MDT) reviewing the clinical, imaging, and pathology data. (Extended Data Fig. 5, Extended Data Fig. 6). Patients with AI-detected pancreatic lesions that were previously not reported by the SOC will be reviewed (and contacted if necessary) by the MDT. The lesions with potential malignancies or undetermined types were invited to undergo a magnetic resonance imaging (MRI) scan with contrast. To reduce the double standard of truth bias in the above described process, we randomly sampled 1% (n=200) of the negative cases that both SOC and AI reported negative. The CT images and EMRs of these cases were further reviewed by the two radiologists, and all cases were confirmed to be negative.

Our real-world data was collected from four scenarios. The physical examination center is indicated for routine check-ups, such as annual checkups. In this scenario, noncontrast CT scans, e.g., chest noncontrast CT scans for lung nodule screening or coronary artery calcium scoring, are usually performed, which can be used for the incidental detection of pancreatic cancer. The emergency department is the emergency room (ER) in the hospital, equipped with imaging devices (noncontrast CT is commonly used) to offer a timely assessment of patients with acute diseases. The outpatient department provides hospital care without being hospitalized or for a stay of less than 24 hours. Outpatient services encompass a wide range of diagnoses, treatments, or follow-ups of various conditions. The inpatient department provides medical treatment to patients who are admitted to a hospital and require an overnight stay or longer-term care. Inpatients typically have more severe illnesses and complex medical conditions. Inpatient care involves multidisciplinary teams of healthcare professionals working together to provide comprehensive management and treatment. In the latter two scenarios, i.e., outpatient and inpatient,

most of our collected CT scans were the noncontrast phase of the multi-phase contrast-enhanced CT scans.

1.2 PANDA training, inference, deployment, and evolution

1.2.1 PANDA Stage-1

Each image is first resampled to the spacing of (1.09mm, 1.09mm, 3mm) in (x, y, z) dimension. The network has 12 consecutive CNN blocks with skip connections and takes the input of a patch size (224, 192, 56). We train the network with deep supervision, SGD optimizer, an initialized learning rate of 1×10^{-2}, and polynomial learning rate decay for 250,000 iterations. In the inference phase of this stage, we first forward the whole noncontrast CT to obtain the whole pancreas mask. Then, we keep the largest connected component and crop the foreground bounding box of the whole pancreatic region, which will be used as the input of the next stages.

1.2.2 PANDA Stage-2

In the training phase, we first pre-train the segmentation UNet backbone for 250,000 iterations only supervised by the masks of lesion and pancreas, and then finetune jointly with the classification branch for another 250,000 iterations with SGD optimizer, polynomial learning rate decay, and an initial learning rate 1×10^{-3}. The initial learning rate of the nnUNet backbone was set to be one-tenth of the classification branch in the finetune stage, i.e., 1×10^{-4}. We trained five-fold cross-validation on the training cohort (n=3,208), resulting in five models. For the operating point selection, the probability cut-off was tuned to achieve a specificity of 99% on the five validation sets in the cross-validation. As such, the cross-validated results on the training cohort of 3208 cases were 0.993 in AUC, 95.1% in sensitivity, and 99.0% in specificity. In the inference phase, the classification probability and the segmentation were produced by an ensemble of the five different models with the pre-selected cut-off for further binary prediction.

1.2.3 PANDA Stage-3

Formally, we describe the mechanism of the cross-attention and self-attention layers as follows. Given a 3D feature map $\mathbf{x}^c \in \mathbb{R}^{H_0 \times W_0 \times D_0 \times d_0}$ of the UNet branch with the shape of (H_0, W_0, D_0) and d_0 channels, we first trilinearly interpolate $\mathbf{x}^c$ into a fixed shape (H, W, D) and linearly project it to d dimension to relieve the computational burden that could be caused by the computation over the large 3D feature map. We then flatten it into 1D features of a length of $\hat{N} = H \times W \times D$ and add a learnable positional embedding $\mathbf{x}_{pos} \in \mathbb{R}^{\hat{N} \times d}$ shared among each layer. The resulted 1D features are denoted as $\hat{\mathbf{x}}^c \in \mathbb{R}^{\hat{N} \times d}$. The 1D global memory feature $\mathbf{x}^m \in \mathbb{R}^{N \times d}$ has a length of N. We compute the the memory-branch query vectors $\mathbf{q}^m \in \mathbb{R}^{N \times d}$, key vectors

$\mathbf{k}^m \in \mathbb{R}^{N \times d}$, and value vectors $\mathbf{v}^m \in \mathbb{R}^{N \times d}$ by forwarding $\mathbf{x}^m$ into three linear projection layers, and compute the key vectors $\mathbf{k}^c \in \mathbb{R}^{\hat{N} \times d}$ and value vectors $\mathbf{v}^c \in \mathbb{R}^{\hat{N} \times d}$ based on $\hat{\mathbf{x}}^c$ similarly. We concatenate the key vectors and the value vectors as $\mathbf{k}^{cm} = [\mathbf{k}^c\, \mathbf{k}^m]$ and $\mathbf{v}^{cm} = [\mathbf{v}^c\, \mathbf{v}^m]$, respectively. Finally, the output of the combined cross-attention and self-attention layer $\mathbf{y}^m \in \mathbb{R}^{N \times d}$ is computed as

$$\mathbf{y}^m = softmax(\frac{\mathbf{q}^m (\mathbf{k}^{cm})^T}{\sqrt{d}})\mathbf{v}_n^{cm} \quad (1)$$

where $softmax$ denotes the softmax function used to regularize each row of the attention matrix. Two standard multilayer perceptron (MLP) layers are followed to increase the computational complexity.

We set $(H, W, D) = (5, 8, 5)$, $d = 320$, and $N = \hat{N} = 200$. After four-level computation, we select the memory responses via mean pooling of N memory features and build a classifier to output the probability of each sub-class. This network is also supervised by a joint segmentation and classification loss described in Methods Eq.1 with a modification of 8-class cross-entropy loss instead of 2-class. Similar to Stage-2, the UNet branch is also pre-trained for 250,000 iterations and then trained jointly with the memory branch for another 250,000 iterations. We use RAdam optimizer and cosine learning rate decay with an initial learning rate 1×10^{-3}. Same as Stage-2, we train the Stage-3 model via five-fold cross-validation on the training set. In the inference phase, we also ensemble the predictions of the five models to produce the final results.

We trained the IPMN subtype classifier with the same network architecture of our Stage-3 model on the IPMN cases in the internal training cohort, where the parameters of the network were initialized from PANDA Stage-3 network and tuned for 25,000 iterations with the rest of the hyper-parameters unchanged.

1.2.4 Real-world deployment

When conducting the real-world study, CT scans within the specified time period were automatically collected. This can be done by checking radiology information system (RIS) records and then transferring those data from the picture archiving and communication system (PACS) to the local server. From each patient's CT DICOM sequences, the required chest or abdominal noncontrast CT scan was automatically selected according to both DICOM tags (e.g., body part, thickness, convolution kernel) and RIS records (e.g., study description, such as chest noncontrast CT). Then we run the PANDA model on the noncontrast CT scan. Both detection and diagnosis results (Excel tables) are transferred and stored in the RIS client.

1.2.5 PANDA Plus

The training set of the "PANDA Plus" model included 3,208 cases from the internal training cohort of PANDA, 275 cases from the multicenter cohort (20

normal, 99 PDAC, 72 PNET, 6 SPT, 15 IPMN, 6 MCN, 13 CP, 23 SCN, and 21 'other'), and 147 cases (62 normal, 1 PNET, 1 CP, 25 'other', and 58 AP) from the real-world cohort 1 (Supplementary Fig. 20). The cases from the multicenter cohort and the real-world cohort 1 were either false positive or false negative predictions of the original PANDA model. For the annotation of the new cases, the same expert radiologist annotated the lesion directly on the noncontrast CT scan, referring to all existing clinical examinations and records, e.g., multi-phase contrast-enhanced CTs, EMRs, and pathology reports. The pancreas segmentation mask was annotated by the original PANDA model. With these extra training data, we finetuned the original PANDA Stage-2 and Stage-3 model (only trained on the internal training cohort) with both the internal training data and the newly collected data for another 250,000 iterations, using the same training hyperparameters as original. In the training process, we oversampled the newly collected CT data by a frequency factor of 5, forcing the AI model to see the challenging data (the false positives and false negatives) and the new categorical data (acute pancreatitis) more frequently. In machine learning, these taxonomies are known as hard example mining and incremental learning, respectively. The evolved model was named PANDA Plus and tested on the second real-world study (RW2).

1.3 Reader studies

1.3.1 Individual differences between noncontrast and contrast-enhanced CT

In the reader studies, we avoided the overlap of the readers between the noncontrast study and the contrast-enhanced study. Because we aimed to measure the individual performances of the readers either on noncontrast or contrast-enhanced CT, while the simultaneous examinations had the possibility of mutual interference. To analyze the individual differences between noncontrast CT and contrast-enhanced CT, four pancreas specialists (S12, S14, S17, and S21) additionally reviewed the noncontrast CT after a long wash-out period (about one year). We found that almost all readers' performance (sensitivity and specificity) in contrast-enhanced CT is superior to their performance in noncontrast CT. In addition, their average performance on the noncontrast CT (85.1% and 96.6%) is similar to the average of the other 11 specialists in the first reader study (82.0% and 96.9%), and interestingly, their average performance on contrast-enhanced CT (89.7% and 98.5%) is similar to the average of the other 11 specialists with AI assistance on noncontrast CT (89.5% and 98.7%).

1.4 Use PANDA for screening in high-risk populations

On the other hand, PANDA could also be used in designed screening in high-risk populations. Given the assumed sensitivity (93%) and specificity (99.9%) of PANDA in PDAC identification, null sensitivity and specificity of 50%, and a prevalence of pancreatic cancer among high-risk new-onset diabetes subjects

aged $\geq$ 50 years of 0.8% [6], we calculated the number of people needed to screen is 1,500 to achieve a statistical power of 90%. The calculation of people needed to screen in the high-risk population was based on Test for One-Sample Sensitivity and Specificity via PASS software (version 15).

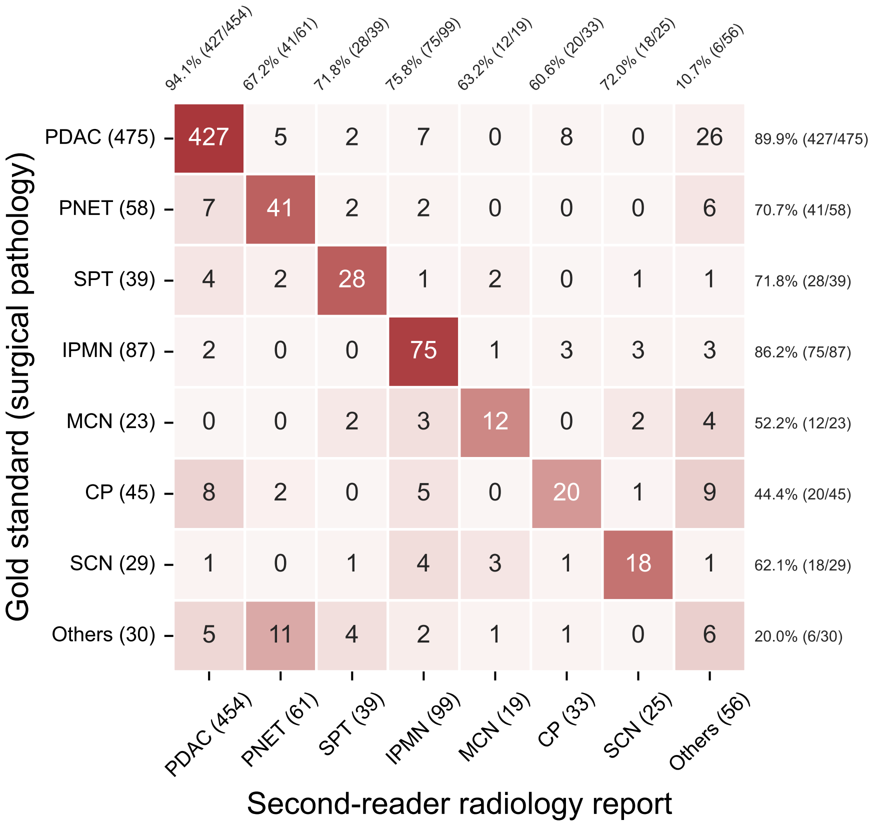

Supplementary Figure 1: Differential diagnosis performance of the second-reader radiology report in the internal differential diagnosis cohort, where comprehensive clinical information and imaging (including contrast-enhanced CT or MRI) are used.

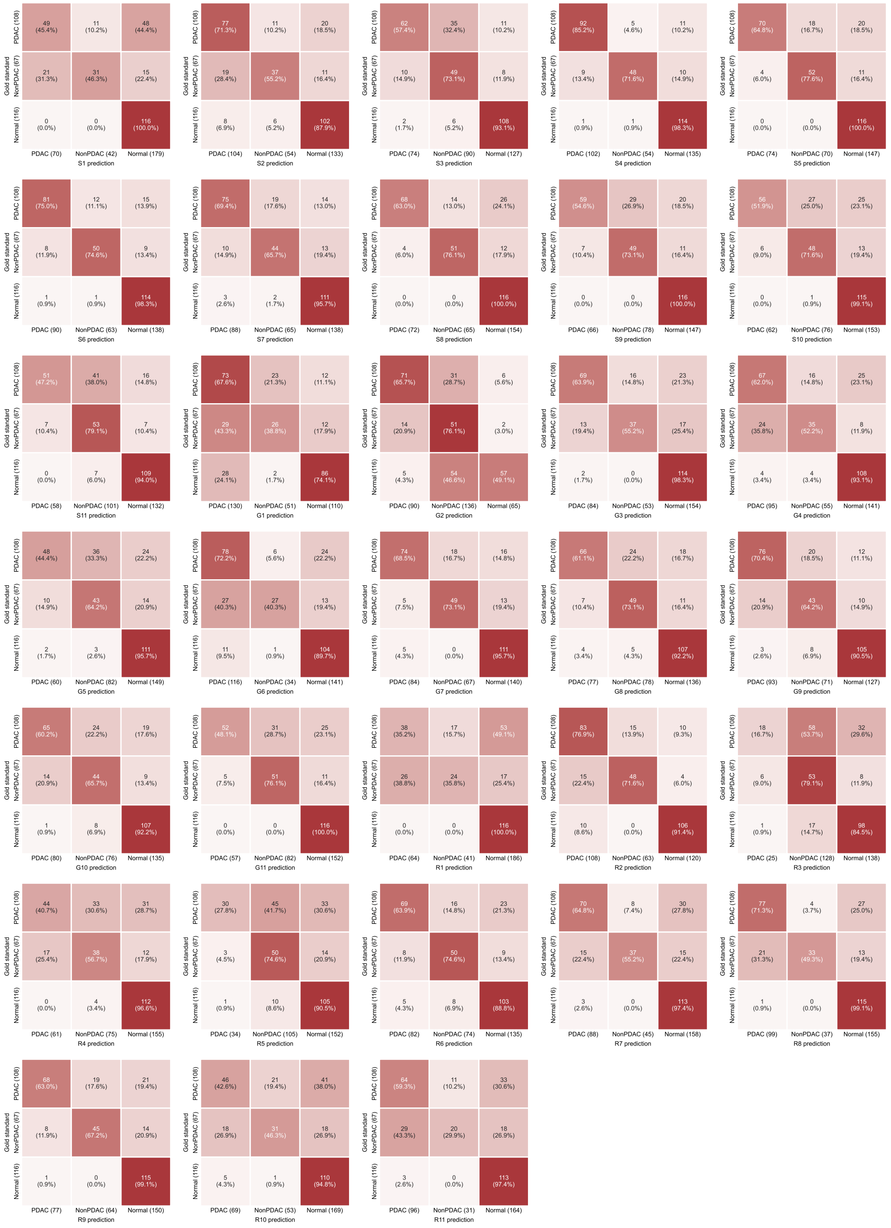

Supplementary Figure 2: Reader study of 33 readers on non-contrast CT for primary diagnosis (normal vs. PDAC vs. nonPDAC).

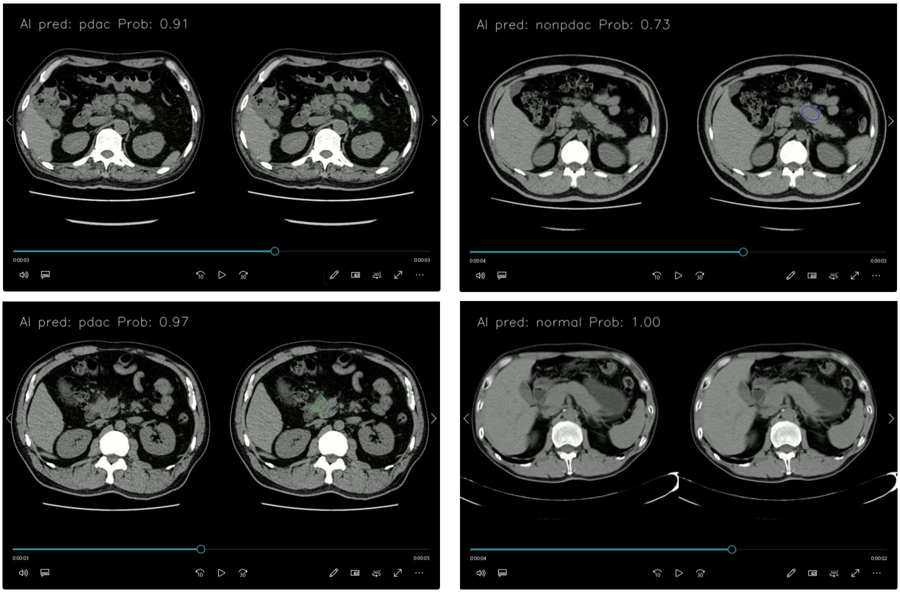

Supplementary Figure 3: Examples of the format of PANDA predictions that we provided to the readers in the first reader study, where reader reads the noncontrast CT with the assistance of PANDA. We generate videos that show the original CT images (left) and the contours of the lesion prediction. PDAC is marked in green contour and nonPDAC is marked in blue. We also show the prediction probability score of PANDA on the top left for reference. The readers can interactively view the CT images and the prediction by adjusting the scroll bar.

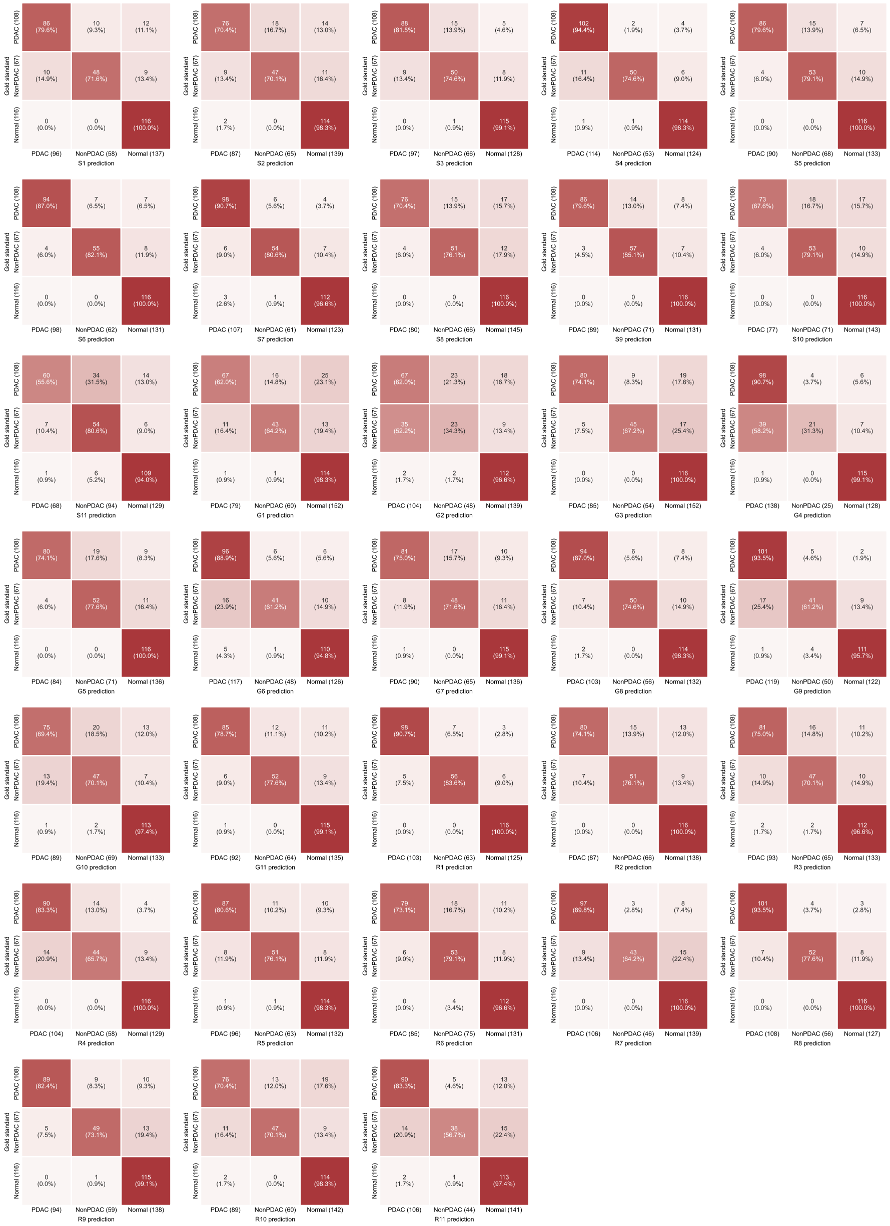

Supplementary Figure 4: Reader study of 33 readers on non-contrast CT with AI assistance for primary diagnosis (normal vs. PDAC vs. nonPDAC).

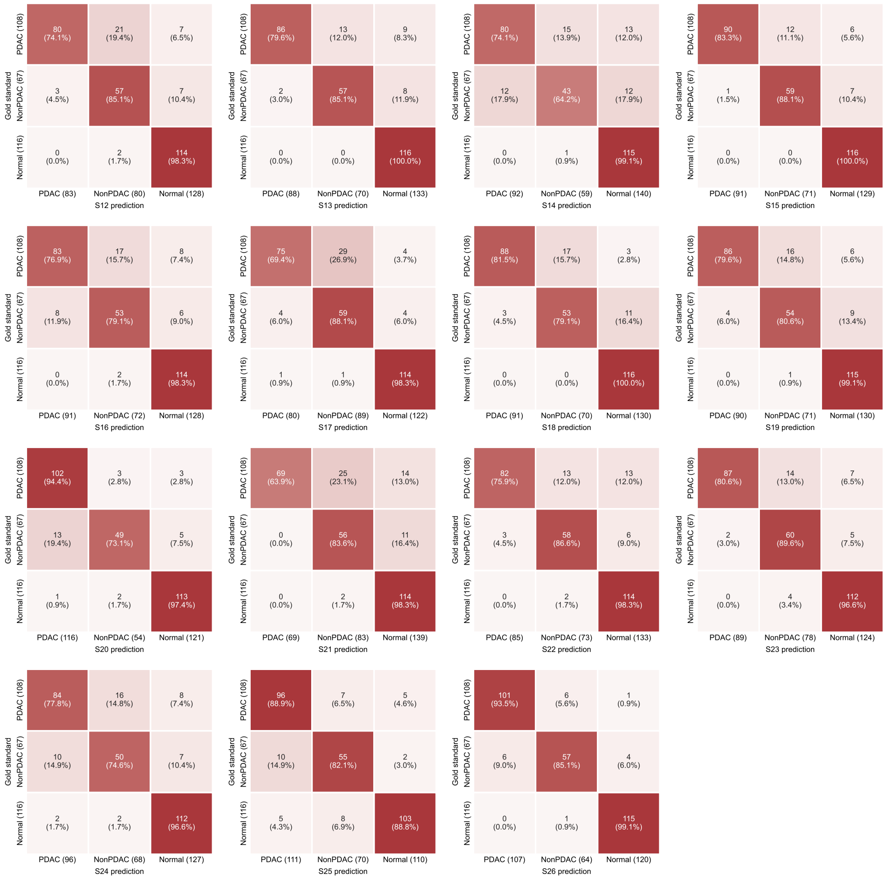

Supplementary Figure 5: Reader study of 15 readers on contrast-enhanced CT for primary diagnosis (normal vs. PDAC vs. nonPDAC).

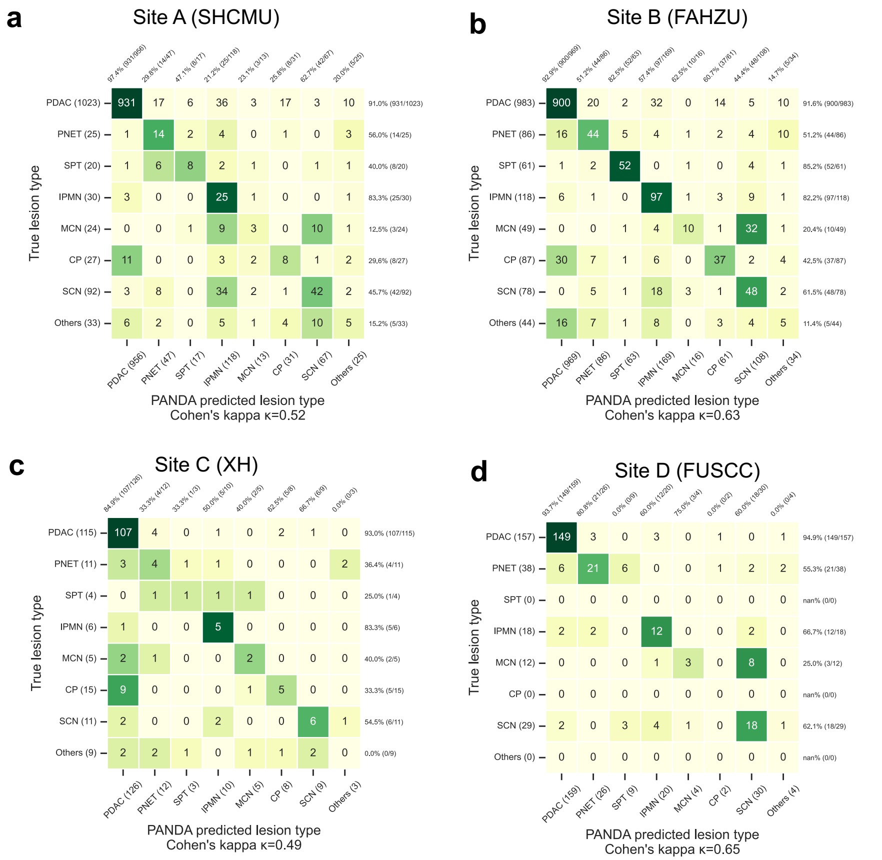

Supplementary Figure 6: Differential diagnosis with PANDA on external test cohorts where nonPDAC cases are available: (a)SHCMU (b)FAHZU (c)XH (d)FUSCC. Cohen's kappa statistics were provided.

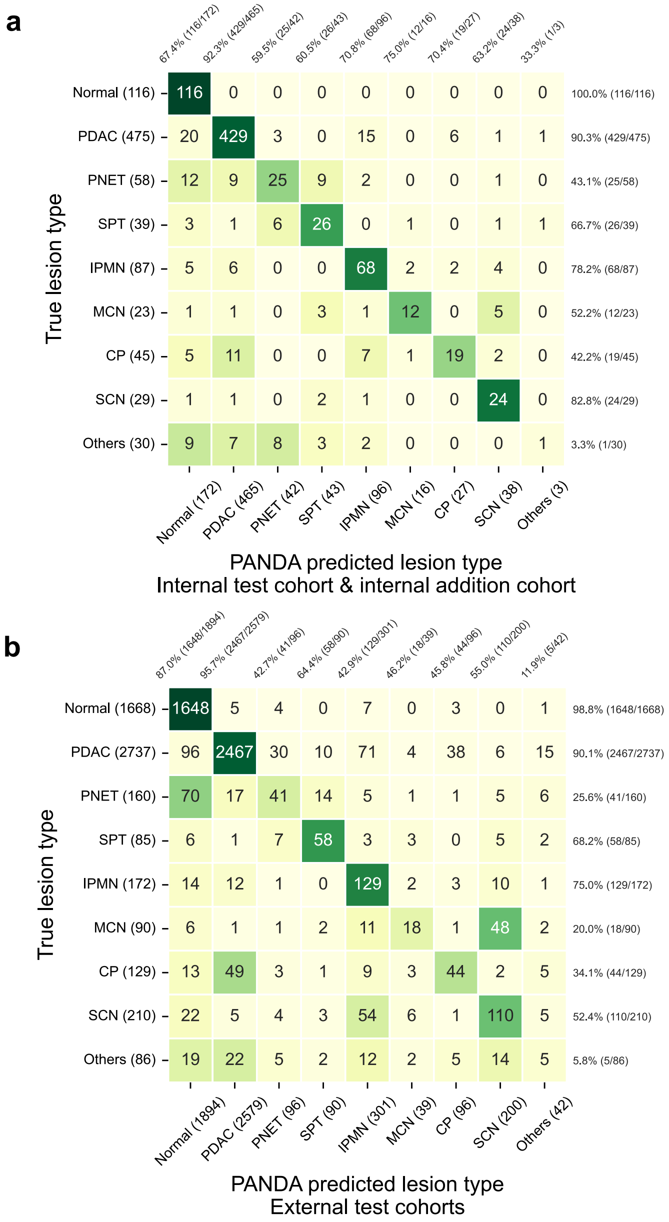

Supplementary Figure 7: Confusion matrix of the full pipeline (lesion detection + differential diagnosis) on (a) the internal test cohort and internal addition cohort, and (b) The external test cohorts.

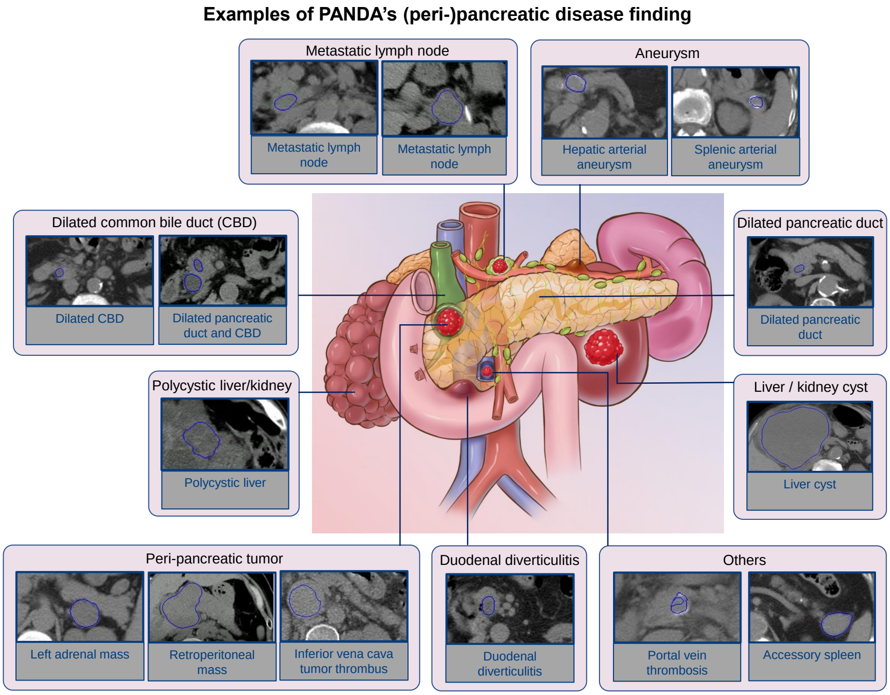

Supplementary Figure 8: Examples of PANDA's (peri-)pancreatic disease findings in the real-world clinical evaluation. PANDA identifies all these example cases as nonPDAC (finding/lesion contours marked in blue). These examples help provide a comprehensive understanding of the performance of PANDA in real-world clinical applications – some of its findings may not be true positive pancreatic lesions, but they are not negligible false positives either.

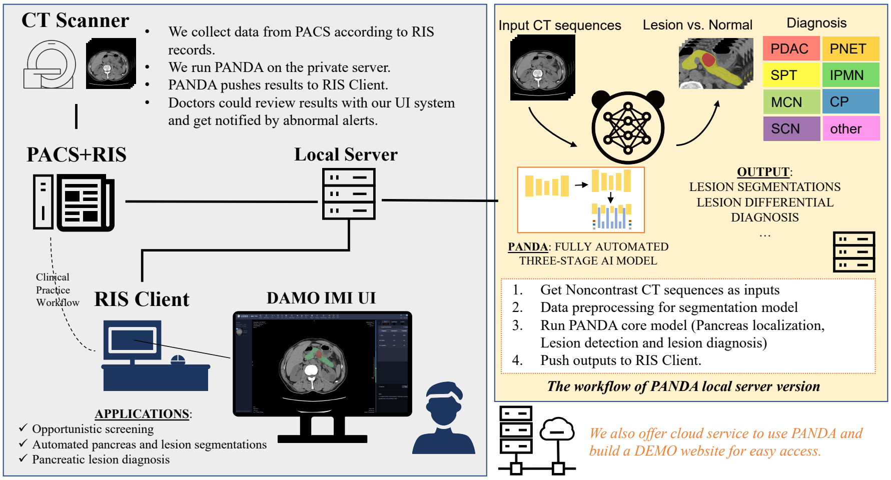

Supplementary Figure 9: Flowchart describing the process of the seamless integration of PANDA into the existing clinical infrastructures. We also offer cloud service to use PANDA and build a demo website (http://panda.medofmind.com/) for easy access. PACS, picture archiving and communication system; RIS, radioiogy information system; DAMO IMI UI, our DAMO Intelligent Medical Imaging user interface (IMI UI).

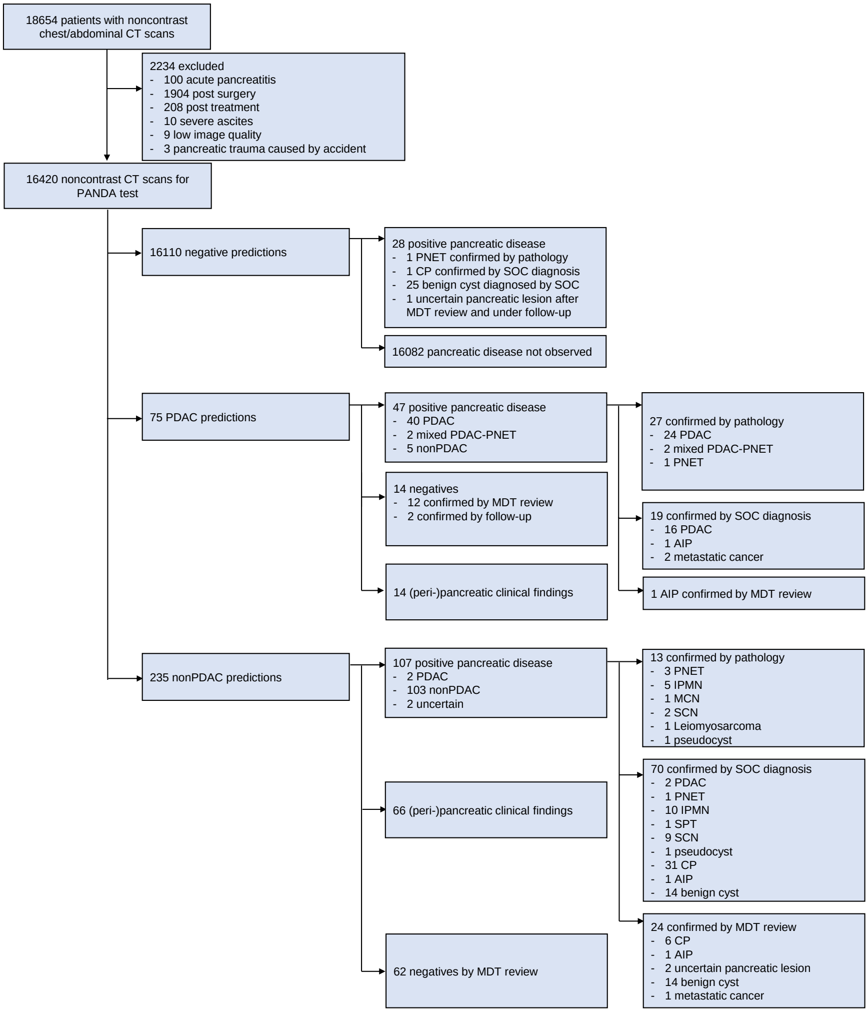

Supplementary Figure 10: Flowchart describing the test process of patients of real-world study 1.

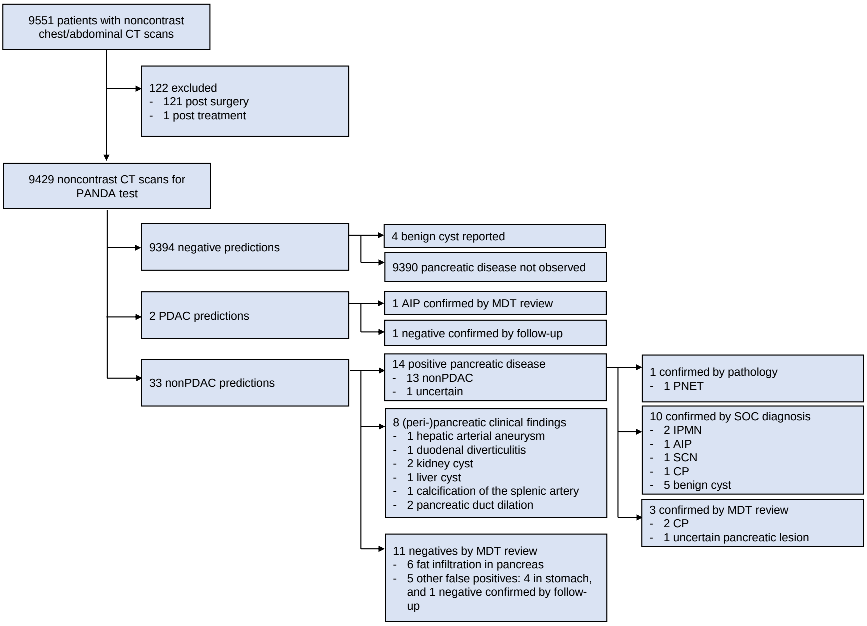

Supplementary Figure 11: Flowchart describing the test process of patients from the physical exam centers of real-world study 1.

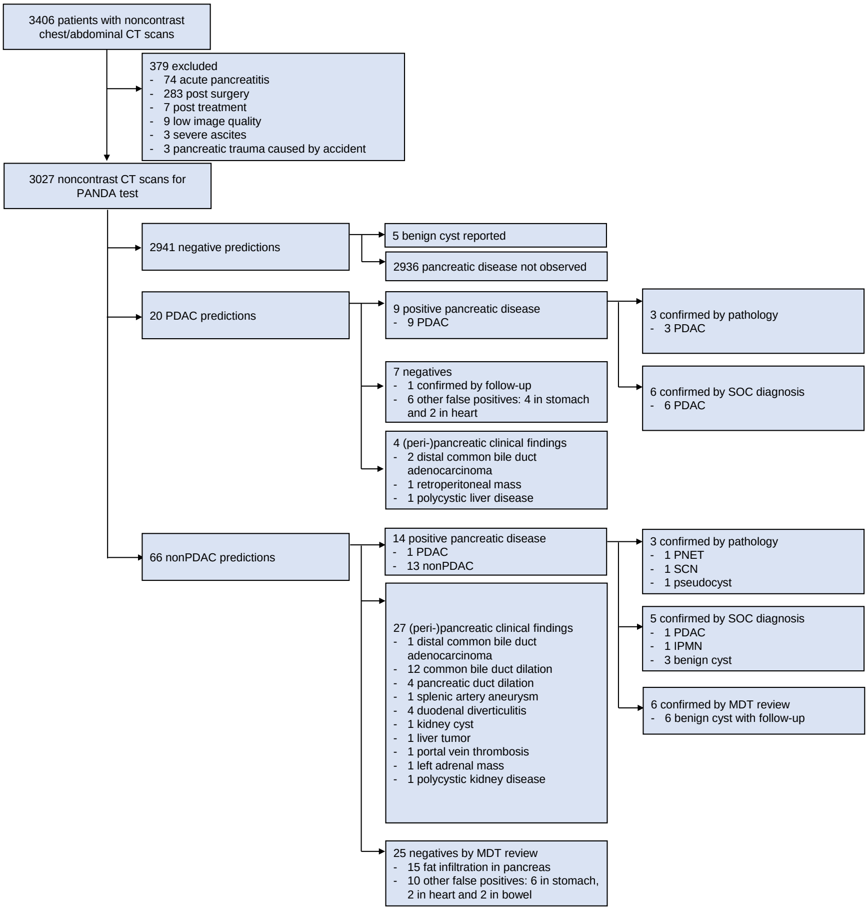

Supplementary Figure 12: Flowchart describing the test process of patients from the emergency department of real-world study 1.

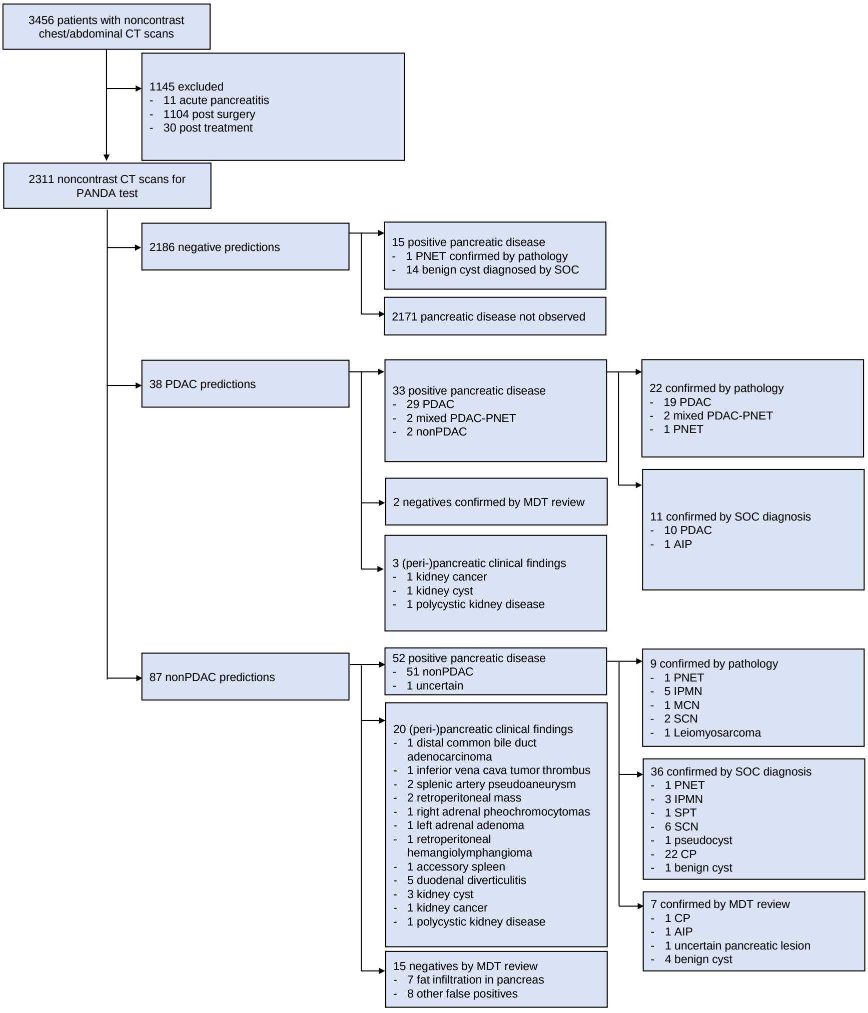

Supplementary Figure 13: Flowchart describing the test process of outpatients of real-world study 1.

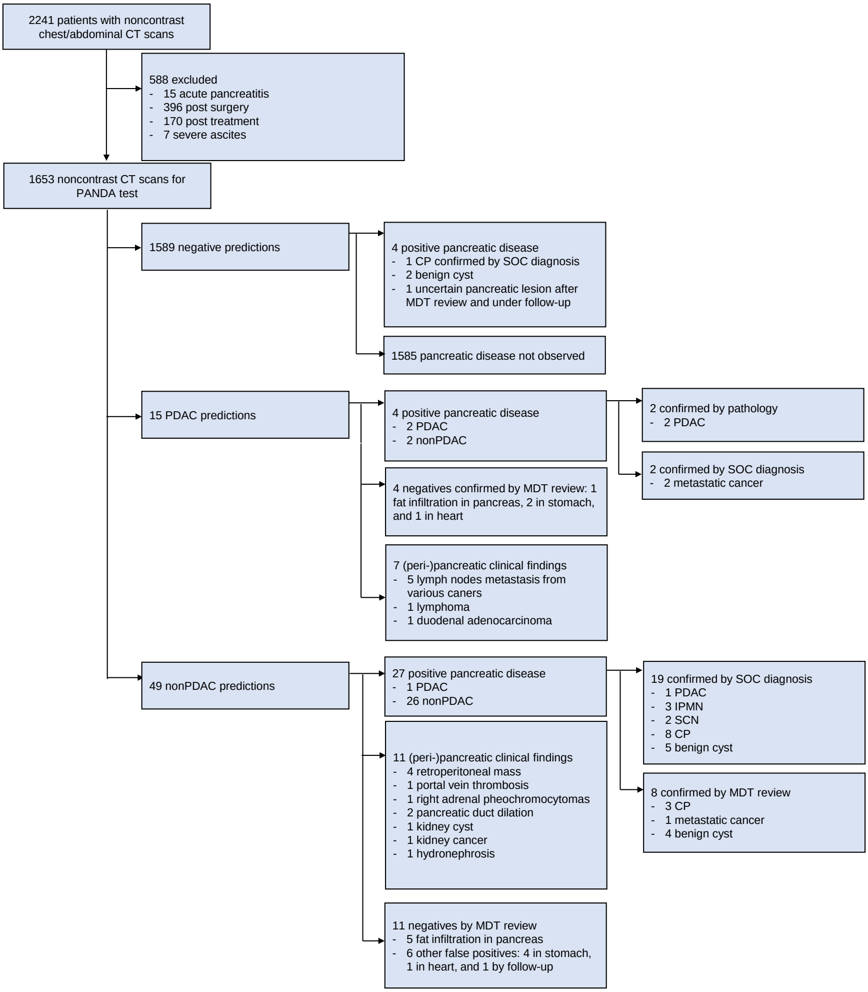

Supplementary Figure 14: Flowchart describing the test process of inpatients of real-world study 1.

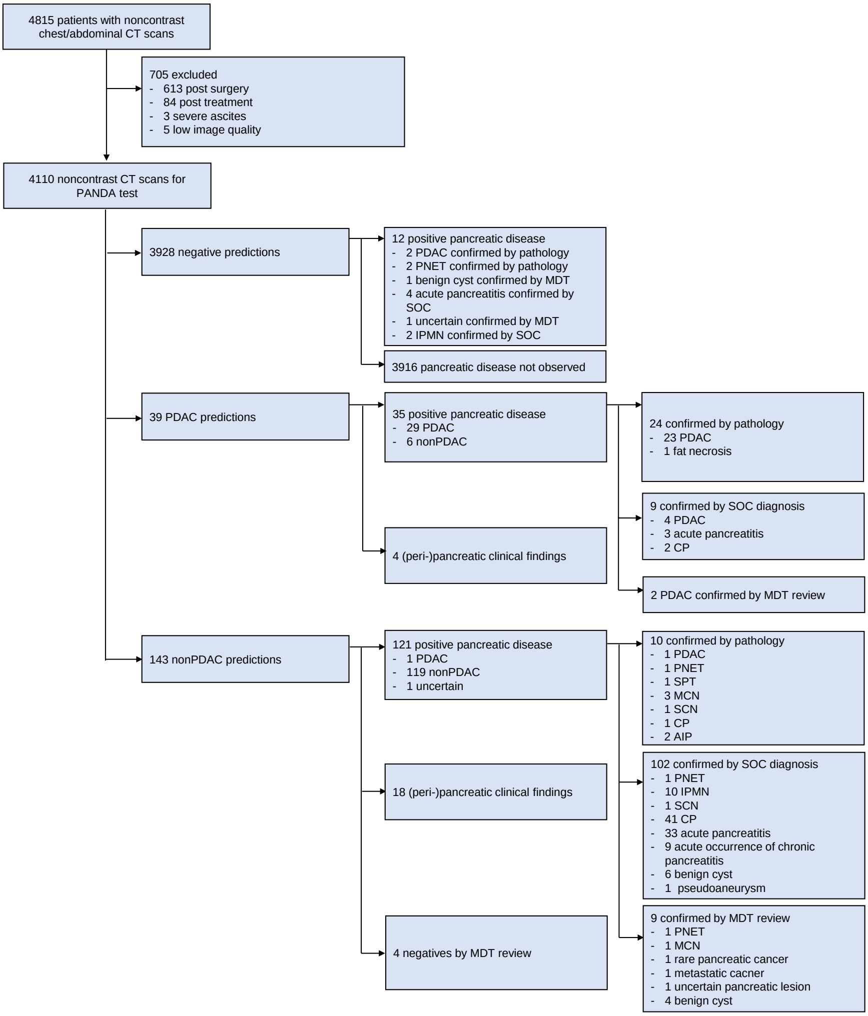

Supplementary Figure 15: Flowchart describing the test process of patients of real-world study 2.

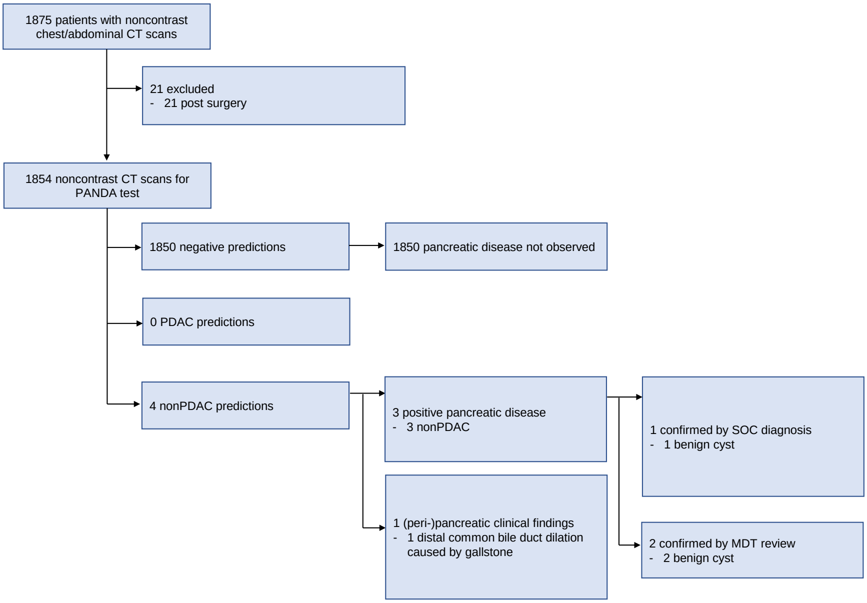

Supplementary Figure 16: Flowchart describing the test process of patients from the physical exam centers of real-world study 2.

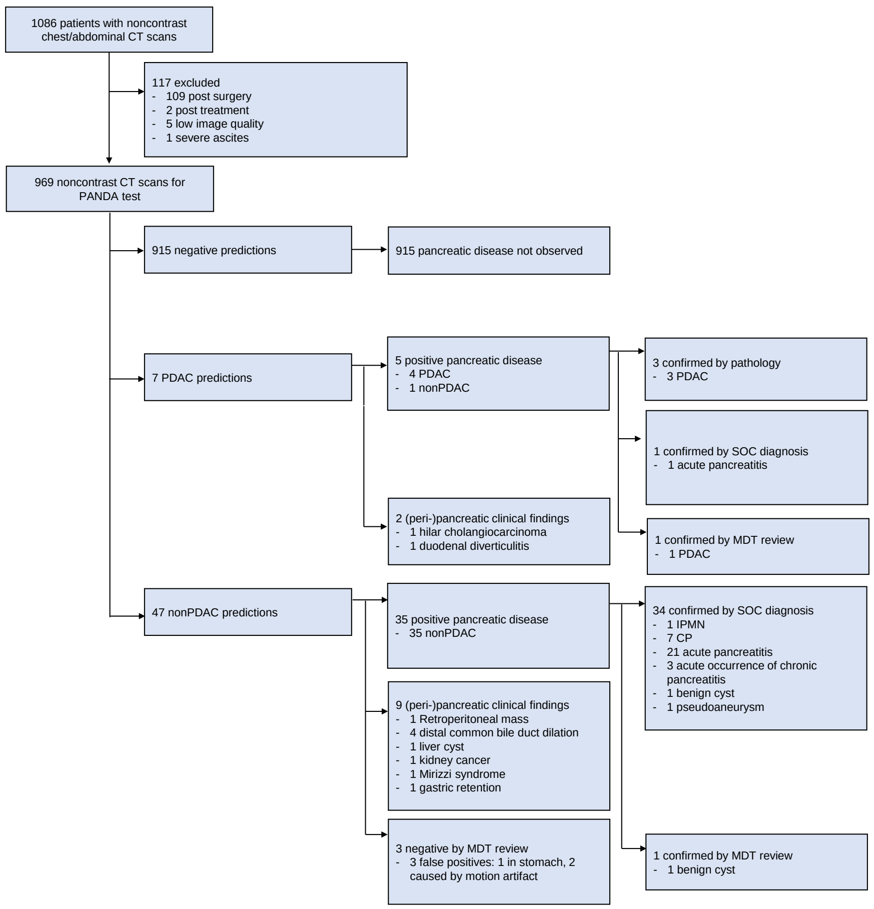

Supplementary Figure 17: Flowchart describing the test process of patients from the emergency department of real-world study 2.

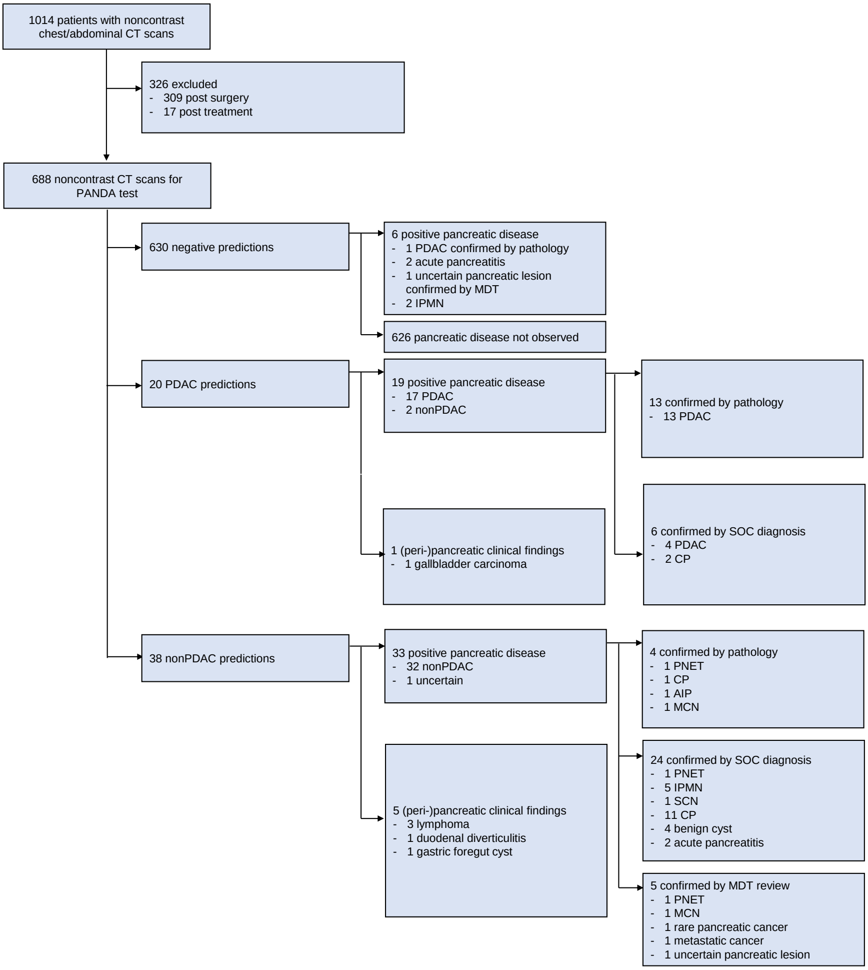

Supplementary Figure 18: Flowchart describing the test process of outpatients of real-world study 2.

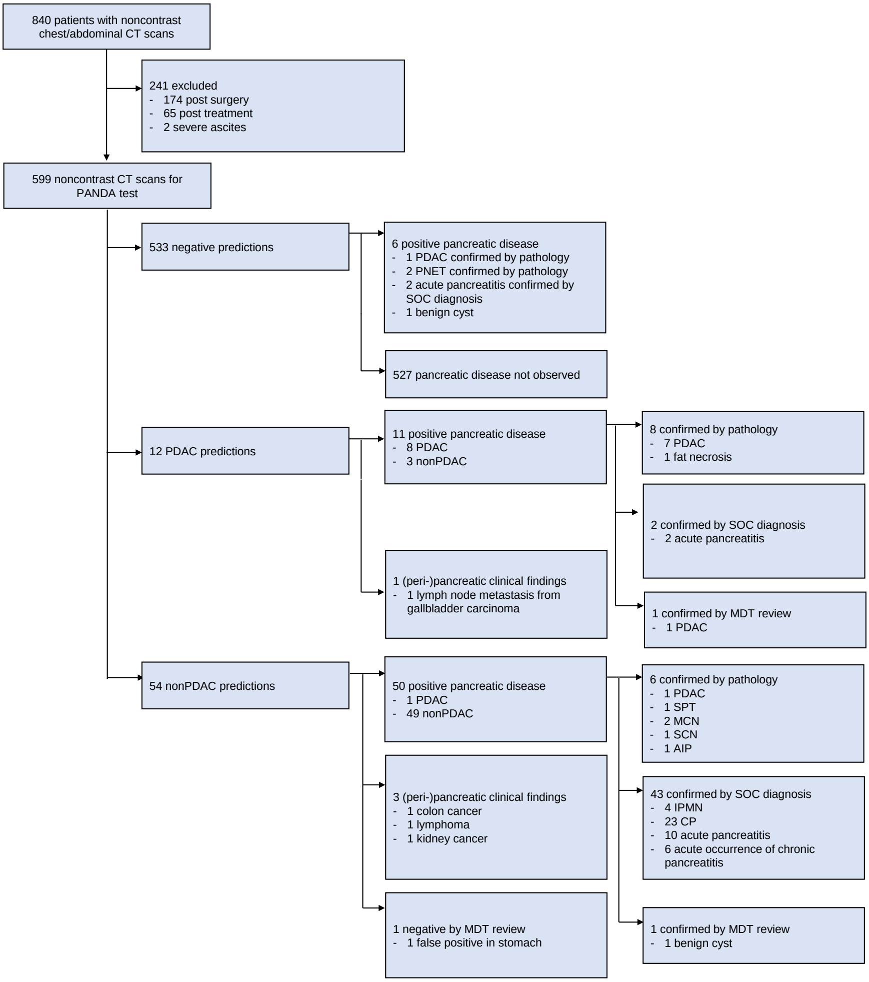

Supplementary Figure 19: Flowchart describing the test process of inpatients of real-world study 2.

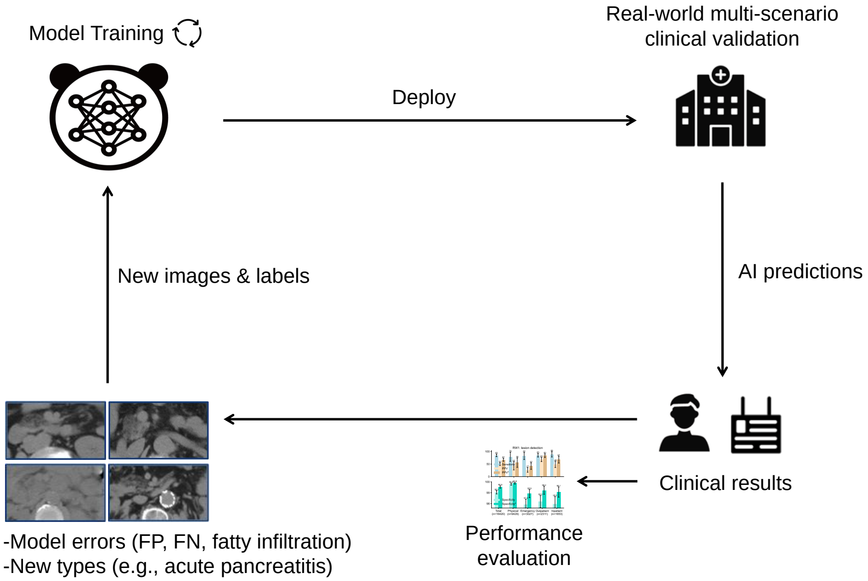

Supplementary Figure 20: Model evolution. We deployed PANDA for real-world multi-scenario clinical validation. The AI results were evaluated by the clinical results, e.g. standard-of-care (SOC) clinical decision, or multidisciplinary team (MDT) determination. The erroneous cases and new types were further collected and annotated for model evolution. The upgraded model, PANDA Plus, significantly reduced the false positives by more than 80%, reaching a desired specificity of 99.9% for both pancreatic lesion detection and PDAC identification.

	**Lesion types**
nonPDAC-'other'	invasive intraductal eosinophilic papillary neoplasm
	invasive intraductal tubulopapillary neoplasm
	lipomatous pseudohypertrophy of the panceas
	leiomyosarcoma
	pancreatic hemangioma
	pancreatic tuberculosis
	pancreatic metastases
	acinar cell carcinoma
	pancreatic liposarcoma
	adenomyomatous hyperplasia
	primary pancreatic lymphoma
	solitary fibrous tumor
	pancreatic schwannoma
	islet hyperplasia
	perivascular epithelioid cell tumor of the pancreas
	gastrointestinal stromal tumor
	intrapancreatic accessory spleen
	pancreatic lipoma
	pancreatic nodular fat necrosis
	pseudopancreatic cyst
	simple cyst
	retention cyst
	lymphoepithelial cyst
	epidermoid cyst

Supplementary Table 1: List of subtypes included in nonPDAC 'other'.

	**Site B (FAHZU)** (sur./bio.)	**Site C (XH)** (sur./bio.)	**RW1 (SIPD)** (sur./bio./cli.)	**RW2 (SIPD)** (sur./bio./cli.)
PDAC	473/510	95/20	9/17/18	7/19/6
PNET	74/12	11/0	4/1/1	3/0/2
SPT	60/1	4/0	0/0/1	1/0/0
IPMN	115/3	6/0	5/0/10	0/0/12
MCN	49/0	5/0	1/0/0	3/0/1
CP	44/43	10/5	0/0/42	2/1/52
SCN	73/5	11/0	2/0/9	1/0/1
Other	30/14	9/0	2/0/57	1/0/14
AP	-	-	-	0/0/40

Supplementary Table 2: Supplementary data characteristics of reference standard of lesion types, i.e. surgical pathology (denoted by "sur."), biopsy pathology (denoted by "bio."), or clinical diagnosis (denoted by "cli."), stratified by lesion type in Site B, Site C, and the real-world cohorts (RW1 and RW2). The internal training and test cohort, Site A, D, E, F, G, H, and I are either all surgically resected, or only contained PDAC lesions. FAHZU, First Affiliated Hospital of Zhejiang University; XH, Xinhua Hospital; SIPD, Shanghai Institution of Pancreatic Diseases.

	Internal Training Cohort (n=2,270)		Internal Test Cohort (including differential diagnosis cohort) (n=786)		External Test Cohorts (n=3,669)	
	Size(mm) (IQR)	Fraction <3cm(%)	Size(mm) (IQR)	Fraction <3cm(%)	Size(mm) (IQR)	Fraction <3cm(%)
PDAC	30 (23-44)	50	30 (25-42)	43	30 (24-40)	49
PNET	25 (17-36)	60	29 (20-40)	50	20 (14-30)	72
SPT	37 (22-56)	39	38 (25-75)	38	33 (23-52)	40
IPMN	25 (17-35)	64	25 (18-35)	60	22 (15-31)	70
MCN	47 (36-65)	11	42 (30-68)	13	30 (26-42)	41
CP	31 (22-39)	42	26 (17-39)	55	26 (22-34)	63
SCN	28 (21-39)	54	35 (27-41)	31	25 (16-33)	66
Other	29 (22-46)	51	25 (20-55)	57	30 (19-42)	50

Supplementary Table 3: Supplementary data characteristics of lesion size (diameter) stratified by lesion type in the internal training, internal test, and external multicenter test cohort. Information was collected in pathology or radiology report (if missing in pathology). The information on lesion size was either collected in the original surgical pathology report (if size was recorded) or was measured on the contrast-enhanced CT image.

	**PANDA**	**Report**	Δ (CI)	**p-value** (difference)	**p-value** (non-inferiority at 5% margin)
Accuracy (%)	79.6	79.8	-0.2 (-3.2–2.8)	1.00	0.0018
Balanced accuracy (%)	60.7	62.2	-1.4 (-8.1–4.9)	0.65	0.28

Supplementary Table 4: Comparison between PANDA (noncontrast CT) and second-reader radiology report (contrast CT) performance on differential diagnosis of eight subtypes of pancreatic diseases in the internal differential diagnosis set ($n = 786$). This report is a secondary analysis of a primary standard of care clinical radiology report, resulting from the double reading process. P values were computed via two-sided permutation tests.

	DSC	95% CI	HD95 (mm)	95% CI
Whole Pancreas	0.903	0.895-0.910	4.37	3.65-5.22
Healthy Pancreas	0.852	0.832-0.868	4.96	4.11-5.98
PDAC	0.719	0.674-0.758	6.45	5.23-7.97
PNET	0.395	0.091-0.698	17.83	8.24-31.42
SPT	0.833	0.690-0.925	9.03	2.87-20.56
IPMN	0.755	0.666-0.819	6.76	4.28-10.07
MCN	0.940	0.923-0.956	3.00	3.00-3.00
CP	0.661	0.547-0.764	19.93	6.16-38.72
SCN	0.732	0.463-0.924	4.64	1.82-9.67
Other	0.540	0.242-0.830	12.00	3.73-27.01

Supplementary Table 5: Segmentation performance of PANDA on the internal test cohort. We calculate the dice coefficient (DSC) and the 95 percentile of Hausdorf distance (HD95) on whole pancreas (including the lesion area), healthy pancreas area, and eight lesion subtypes.

Lesion detection								
**Reader**	**Sens**	**Δ**	**95% CI**	**p-value**	**Spec**	**Δ**	**95% CI**	**p-value**
PANDA	94.9	-	-	-	100	-	-	-
S1	64.0	30.9	(23.7 - 37.7)	0.0002	100.0	0.0	(0.0 - 0.0)	1.0000
S2	82.3	12.6	(6.7 - 18.7)	0.0004	87.9	12.1	(6.2 - 18.4)	0.0006
S3	89.1	5.7	(1.8 - 10.2)	0.0110	93.1	6.9	(2.6 - 11.8)	0.0074
S4	88.0	6.9	(3.3 - 10.5)	0.0006	98.3	1.7	(0.0 - 4.4)	0.4978
S5	82.3	12.6	(7.2 - 18.3)	0.0002	100.0	0.0	(0.0 - 0.0)	1.0000
S6	86.3	8.6	(4.2 - 13.3)	0.0002	98.3	1.7	(0.0 - 4.3)	0.4914
S7	84.6	10.3	(5.5 - 15.7)	0.0004	95.7	4.3	(0.9 - 8.2)	0.0634
S8	78.3	16.6	(10.4 - 22.2)	0.0002	100.0	0.0	(0.0 - 0.0)	1.0000
S9	82.3	12.6	(7.4 - 17.9)	0.0002	100.0	0.0	(0.0 - 0.0)	1.0000
S10	78.3	16.6	(11.3 - 22.0)	0.0002	99.1	0.9	(0.0 - 2.7)	0.9960
S11	86.9	8.0	(2.9 - 12.9)	0.0022	94.0	6.0	(2.0 - 10.4)	0.0136
G1	86.3	8.6	(4.0 - 13.4)	0.0012	74.1	25.9	(17.9 - 33.9)	0.0002
G2	95.4	-0.6	(-4.1 - 2.6)	0.9894	49.1	50.9	(42.2 - 60.0)	0.0002
G3	77.1	17.7	(12.8 - 23.7)	0.0002	98.3	1.7	(0.0 - 4.5)	0.4946
G4	81.1	13.7	(8.4 - 19.5)	0.0002	93.1	6.9	(2.6 - 11.9)	0.0082
G5	78.3	16.6	(11.0 - 22.1)	0.0002	95.7	4.3	(1.0 - 8.4)	0.0620
G6	78.9	16.0	(10.5 - 21.8)	0.0002	89.7	10.3	(5.5 - 16.5)	0.0010
G7	83.4	11.4	(6.3 - 16.3)	0.0002	95.7	4.3	(0.9 - 8.2)	0.0564
G8	83.4	11.4	(6.1 - 17.0)	0.0002	92.2	7.8	(3.2 - 13.5)	0.0044
G9	87.4	7.4	(2.8 - 12.0)	0.0022	90.5	9.5	(4.7 - 14.5)	0.0012
G10	84.0	10.9	(5.8 - 16.1)	0.0004	92.2	7.8	(3.3 - 13.2)	0.0046
G11	79.4	15.4	(9.7 - 21.4)	0.0002	100.0	0.0	(0.0 - 0.0)	1.0000
R1	60.0	34.9	(27.7 - 41.8)	0.0002	100.0	0.0	(0.0 - 0.0)	1.0000
R2	92.0	2.9	(-1.7 - 7.3)	0.2944	91.4	8.6	(3.8 - 13.6)	0.0022
R3	77.1	17.7	(12.0 - 23.4)	0.0002	84.5	15.5	(9.6 - 22.7)	0.0002
R4	75.4	19.4	(13.1 - 25.8)	0.0002	96.6	3.4	(0.8 - 7.2)	0.1144
R5	73.1	21.7	(15.7 - 28.4)	0.0002	90.5	9.5	(4.3 - 15.5)	0.0010
R6	81.7	13.1	(7.8 - 19.0)	0.0002	88.8	11.2	(5.5 - 17.0)	0.0004
R7	74.3	20.6	(14.6 - 26.9)	0.0002	97.4	2.6	(0.0 - 5.6)	0.2504
R8	77.1	17.7	(12.3 - 23.7)	0.0002	99.1	0.9	(0.0 - 3.0)	1.0000
R9	80.0	14.9	(9.8 - 20.2)	0.0002	99.1	0.9	(0.0 - 2.9)	0.9966
R10	66.3	28.6	(21.5 - 35.8)	0.0002	94.8	5.2	(1.7 - 9.6)	0.0302
R11	70.9	24.0	(16.8 - 31.0)	0.0002	97.4	2.6	(0.0 - 5.8)	0.2488
Mean S	82.0	12.8	(8.9 - 17.0)	0.0002	96.9	3.1	(2.0 - 4.1)	0.0002
Mean G	83.2	11.7	(7.7 - 15.9)	0.0002	88.2	11.8	(9.6 - 14.4)	0.0002
Mean R	75.3	19.6	(15.2 - 23.9)	0.0002	94.5	5.5	(4.2 - 6.7)	0.0002
Mean	80.2	14.7	(10.8 - 18.8)	0.0002	93.2	6.8	(5.6 - 8.1)	0.0002

(a) Reader (noncontrast CT) vs. PANDA (noncontrast CT) by the evaluation of sensitivity and specificity for lesion detection (PDAC+non-PDAC vs. normal)

PDAC identification								
**Reader**	**Sens**	**Δ**	**95% CI**	**p-value**	**Spec**	**Δ**	**95% CI**	**p-value**
PANDA	92.6	-	-	-	97.3	-	-	-
S1	45.4	47.2	(36.8 - 57.6)	0.0002	88.5	8.7	(4.7 - 13.2)	0.0004
S2	71.3	21.3	(11.6 - 30.8)	0.0004	85.2	12.0	(6.4 - 17.8)	0.0002
S3	57.4	35.2	(26.7 - 44.4)	0.0002	93.4	3.8	(0.0 - 7.9)	0.0954
S4	85.2	7.4	(0.8 - 14.9)	0.0792	94.5	2.7	(-0.6 - 6.3)	0.2238
S5	64.8	27.8	(18.3 - 36.6)	0.0002	97.8	-0.5	(-2.3 - 1.1)	0.9882
S6	75.0	17.6	(9.3 - 25.5)	0.0002	95.1	2.2	(-0.6 - 5.5)	0.3452
S7	69.4	23.1	(14.9 - 32.3)	0.0002	92.9	4.4	(0.5 - 8.4)	0.0626
S8	63.0	29.6	(21.1 - 38.4)	0.0002	97.8	-0.5	(-3.0 - 1.8)	0.9850
S9	54.6	38.0	(28.7 - 47.4)	0.0002	96.2	1.1	(-1.7 - 4.1)	0.7260
S10	51.9	40.7	(30.7 - 51.0)	0.0002	96.7	0.5	(-2.4 - 3.7)	1.0000
S11	47.2	45.4	(36.2 - 54.8)	0.0002	96.2	1.1	(-1.6 - 4.2)	0.7424
G1	67.6	25.0	(15.8 - 34.0)	0.0002	68.9	28.4	(21.6 - 35.4)	0.0002
G2	65.7	26.9	(18.5 - 35.1)	0.0002	89.6	7.7	(3.2 - 12.3)	0.0026
G3	63.9	28.7	(21.0 - 37.3)	0.0002	91.8	5.5	(1.6 - 9.8)	0.0220
G4	62.0	30.6	(20.4 - 40.2)	0.0002	84.7	12.6	(7.1 - 17.9)	0.0002
G5	44.4	48.1	(38.9 - 57.5)	0.0002	93.4	3.8	(0.0 - 7.5)	0.0634
G6	72.2	20.4	(12.0 - 29.1)	0.0002	79.2	18.0	(12.1 - 24.2)	0.0002
G7	68.5	24.1	(15.9 - 32.7)	0.0002	94.5	2.7	(-1.1 - 6.6)	0.2592
G8	61.1	31.5	(21.5 - 41.7)	0.0002	94.0	3.3	(-0.6 - 7.7)	0.2152
G9	70.4	22.2	(12.6 - 31.7)	0.0004	90.7	6.6	(2.6 - 10.8)	0.0066
G10	60.2	32.4	(22.3 - 41.9)	0.0002	91.8	5.5	(1.7 - 9.5)	0.0132
G11	48.1	44.4	(34.5 - 54.1)	0.0002	97.3	0.0	(-2.7 - 2.8)	1.0000
R1	35.2	57.4	(47.9 - 67.3)	0.0002	85.8	11.5	(6.8 - 16.4)	0.0002
R2	76.9	15.7	(8.0 - 23.7)	0.0002	86.3	10.9	(6.0 - 15.8)	0.0002
R3	16.7	75.9	(68.0 - 83.5)	0.0002	96.2	1.1	(-1.8 - 4.0)	0.7240
R4	40.7	51.9	(40.4 - 62.5)	0.0002	90.7	6.6	(1.8 - 11.1)	0.0164
R5	27.8	64.8	(54.5 - 74.7)	0.0002	97.8	-0.5	(-3.4 - 2.6)	1.0000
R6	63.9	28.7	(19.4 - 38.1)	0.0002	92.9	4.4	(0.5 - 8.5)	0.0570
R7	64.8	27.8	(19.3 - 36.5)	0.0002	90.2	7.1	(2.9 - 11.7)	0.0030
R8	71.3	21.3	(11.5 - 30.7)	0.0002	88.0	9.3	(4.3 - 14.0)	0.0006
R9	63.0	29.6	(20.8 - 38.4)	0.0002	95.1	2.2	(-1.1 - 5.9)	0.3954
R10	42.6	50.0	(40.8 - 60.5)	0.0002	87.4	9.8	(4.8 - 14.8)	0.0006
R11	59.3	33.3	(22.6 - 43.8)	0.0002	82.5	14.8	(9.5 - 20.7)	0.0002
Mean S	62.3	30.3	(24.9 - 35.9)	0.0002	94.0	3.2	(1.5 - 5.1)	0.0038
Mean G	62.2	30.4	(25.1 - 35.5)	0.0002	88.7	8.5	(6.0 - 11.2)	0.0002
Mean R	51.1	41.5	(36.7 - 46.1)	0.0002	90.3	7.0	(4.8 - 9.3)	0.0002
Mean	58.5	34.1	(29.3 - 38.9)	0.0002	91.0	6.3	(4.1 - 8.4)	0.0002

(b) Reader (noncontrast CT) vs. PANDA (noncontrast CT) by the evaluation of sensitivity and specificity for PDAC identification (PDAC vs. nonPDAC + normal).

Supplementary Table 6: Reader (noncontrast CT) vs. PANDA (noncontrast CT) by sensitivity and specificity. Two-sided permutation tests were used to compute the statistical difference. S, pancreas specialist; G, general radiologist; R, radiology resident; Sens, sensitivity (%); Spec, specificity (%).

Lesion detection										
**Reader**	**Sens**	**Sens-A**	**Δ**	**95% CI**	**p-value**	**Spec**	**Spec-A**	**Δ**	**95% CI**	**p-value**
S1	64.0	88.0	24.0	(17.9 - 30.9)	0.0002	100	100	0.0	(0.0 - 0.0)	1.0000
S2	82.3	85.7	3.4	(-4.2 - 10.8)	0.4604	87.9	98.3	10.3	(3.6 - 16.7)	0.0040
S3	89.1	92.6	3.4	(0.6 - 6.5)	0.0654	93.1	99.1	6.0	(2.0 - 10.8)	0.0168
S4	88.0	94.3	6.3	(2.4 - 10.4)	0.0036	98.3	98.3	0.0	(-2.0 - 2.4)	1.0000
S5	82.3	90.3	8.0	(4.4 - 12.1)	0.0006	100	100	0.0	(0.0 - 0.0)	1.0000
S6	86.3	91.4	5.1	(1.2 - 9.3)	0.0232	98.3	100	1.7	(0.0 - 4.5)	0.4992
S7	84.6	93.7	9.1	(4.2 - 14.0)	0.0004	95.7	96.6	0.9	(-4.1 - 6.0)	0.9862
S8	78.3	83.4	5.1	(2.2 - 8.5)	0.0034	100	100	0.0	(0.0 - 0.0)	1.0000
S9	82.3	91.4	9.1	(5.0 - 13.9)	0.0002	100	100	0.0	(0.0 - 0.0)	1.0000
S10	78.3	84.6	6.3	(2.9 - 9.9)	0.0016	99.1	100	0.9	(0.0 - 2.7)	1.0000
S11	86.9	88.6	1.7	(0.0 - 3.8)	0.2478	94.0	94.0	0.0	(-2.5 - 2.6)	1.0000
G1	86.3	78.3	-8.0	(-14.2 - -2.2)	0.0196	74.1	98.3	24.1	(16.8 - 32.0)	0.0002
G2	95.4	84.6	-10.9	(-15.6 - -6.1)	0.0002	49.1	96.6	47.4	(37.1 - 57.3)	0.0002
G3	77.1	79.4	2.3	(-3.4 - 8.0)	0.5672	98.3	100	1.7	(0.0 - 4.3)	0.5018
G4	81.1	92.6	11.4	(6.3 - 16.4)	0.0002	93.1	99.1	6.0	(1.9 - 10.6)	0.0152
G5	78.3	88.6	10.3	(4.7 - 15.7)	0.0004	95.7	100	4.3	(0.9 - 8.0)	0.0622
G6	78.9	90.9	12.0	(7.1 - 17.5)	0.0002	89.7	94.8	5.2	(-0.9 - 11.8)	0.1760
G7	83.4	88.0	4.6	(1.1 - 8.6)	0.0406	95.7	99.1	3.4	(0.8 - 7.5)	0.1218
G8	83.4	89.7	6.3	(0.6 - 12.1)	0.0540	92.2	98.3	6.0	(1.0 - 11.3)	0.0396
G9	87.4	93.7	6.3	(2.2 - 10.9)	0.0098	90.5	95.7	5.2	(0.0 - 11.5)	0.1444
G10	84.0	88.6	4.6	(0.6 - 8.6)	0.0378	92.2	97.4	5.2	(0.9 - 10.3)	0.0692
G11	79.4	88.6	9.1	(5.0 - 13.3)	0.0002	100	99.1	-0.9	(-2.8 - 0.0)	1.0000
R1	60.0	94.9	34.9	(27.7 - 42.3)	0.0002	100	100	0.0	(0.0 - 0.0)	1.0000
R2	92.0	87.4	-4.6	(-8.2 - -1.2)	0.0210	91.4	100	8.6	(3.7 - 13.9)	0.0028
R3	77.1	88.0	10.9	(5.0 - 17.0)	0.0008	84.5	96.6	12.1	(4.6 - 19.3)	0.0044
R4	75.4	92.6	17.1	(11.5 - 23.7)	0.0002	96.6	100	3.4	(0.8 - 7.4)	0.1230
R5	73.1	89.7	16.6	(9.9 - 23.1)	0.0002	90.5	98.3	7.8	(3.4 - 12.8)	0.0048
R6	81.7	89.1	7.4	(2.2 - 12.6)	0.0106	88.8	96.6	7.8	(1.8 - 13.9)	0.0210
R7	74.3	86.9	12.6	(7.4 - 18.2)	0.0002	97.4	100	2.6	(0.0 - 5.4)	0.2452
R8	77.1	93.7	16.6	(10.8 - 22.4)	0.0002	99.1	100	0.9	(0.0 - 2.8)	1.0000
R9	80.0	86.9	6.9	(2.1 - 11.7)	0.0120	99.1	99.1	0.0	(-2.7 - 2.5)	1.0000
R10	66.3	84.0	17.7	(10.6 - 24.4)	0.0002	94.8	98.3	3.4	(-0.9 - 8.6)	0.2908
R11	70.9	84.0	13.1	(8.3 - 18.8)	0.0002	97.4	97.4	0.0	(-4.4 - 3.9)	1.0000
Mean S	82.0	89.5	7.4	(5.6 - 9.4)	0.0002	96.9	98.7	1.8	(0.9 - 2.8)	0.0008
Mean G	83.2	87.5	4.4	(2.5 - 6.3)	0.0002	88.2	98.0	9.8	(7.7 - 12.2)	0.0002
Mean R	75.3	88.8	13.6	(10.7 - 16.6)	0.0002	94.5	98.7	4.2	(2.9 - 5.5)	0.0002
Mean	80.2	88.6	8.5	(6.5 - 10.3)	0.0002	93.2	98.5	5.3	(4.3 - 6.3)	0.0002

(a) Reader (noncontrast CT) vs. Reader+PANDA assistance (noncontrast CT) by the evaluation of sensitivity and specificity for lesion detection (PDAC+non-PDAC vs. normal)

PDAC identification										
**Reader**	**Sens**	**Sens-A**	**Δ**	**95% CI**	**p-value**	**Spec**	**Spec-A**	**Δ**	**95% CI**	**p-value**
S1	45.4	79.6	34.3	(24.0 - 45.1)	0.0002	88.5	94.5	6.0	(1.5 - 10.8)	0.0194
S2	71.3	70.4	-0.9	(-13.0 - 11.1)	0.9878	85.2	94.0	8.7	(3.0 - 14.1)	0.0028
S3	57.4	81.5	24.1	(16.0 - 32.3)	0.0002	93.4	95.1	1.6	(-1.1 - 4.6)	0.4652
S4	85.2	94.4	9.3	(2.8 - 16.5)	0.0200	94.5	93.4	-1.1	(-4.2 - 1.7)	0.7242
S5	64.8	79.6	14.8	(7.8 - 22.1)	0.0004	97.8	97.8	0.0	(0.0 - 0.0)	1.0000
S6	75.0	87.0	12.0	(4.9 - 19.6)	0.0038	95.1	97.8	2.7	(-0.5 - 6.1)	0.1814
S7	69.4	90.7	21.3	(11.4 - 30.2)	0.0002	92.9	95.1	2.2	(-2.1 - 6.3)	0.4634
S8	63.0	70.4	7.4	(2.9 - 12.4)	0.0070	97.8	97.8	0.0	(0.0 - 0.0)	1.0000
S9	54.6	79.6	25.0	(16.0 - 33.9)	0.0002	96.2	98.4	2.2	(-0.5 - 4.8)	0.2186
S10	51.9	67.6	15.7	(9.0 - 22.0)	0.0002	96.7	97.8	1.1	(-1.0 - 3.5)	0.6118
S11	47.2	55.6	8.3	(3.6 - 14.1)	0.0044	96.2	95.6	-0.5	(-1.7 - 0.0)	1.0000
G1	67.6	62.0	-5.6	(-16.8 - 6.5)	0.4324	68.9	93.4	24.6	(17.9 - 32.0)	0.0002
G2	65.7	62.0	-3.7	(-13.0 - 5.7)	0.5578	89.6	79.8	-9.8	(-16.4 - -3.4)	0.0062
G3	63.9	74.1	10.2	(2.0 - 17.8)	0.0302	91.8	97.3	5.5	(1.9 - 9.2)	0.0078
G4	62.0	90.7	28.7	(20.2 - 37.6)	0.0002	84.7	78.1	-6.6	(-12.0 - -1.1)	0.0384
G5	44.4	74.1	29.6	(20.0 - 38.5)	0.0002	93.4	97.8	4.4	(1.1 - 7.7)	0.0250
G6	72.2	88.9	16.7	(9.5 - 24.6)	0.0002	79.2	88.5	9.3	(3.3 - 15.3)	0.0040
G7	68.5	75.0	6.5	(1.8 - 11.6)	0.0356	94.5	95.1	0.5	(-2.2 - 3.7)	1.0000
G8	61.1	87.0	25.9	(16.1 - 35.7)	0.0002	94.0	95.1	1.1	(-2.6 - 4.8)	0.7588
G9	70.4	93.5	23.1	(14.1 - 31.8)	0.0002	90.7	90.2	-0.5	(-5.0 - 3.8)	1.0000
G10	60.2	69.4	9.3	(2.1 - 17.1)	0.0320	91.8	92.3	0.5	(-2.9 - 4.3)	0.9970
G11	48.1	78.7	30.6	(20.8 - 40.4)	0.0002	97.3	96.2	-1.1	(-4.2 - 1.8)	0.7328
R1	35.2	90.7	55.6	(45.0 - 65.5)	0.0002	85.8	97.3	11.5	(6.1 - 16.9)	0.0004
R2	76.9	74.1	-2.8	(-10.8 - 5.8)	0.6620	86.3	96.2	9.8	(5.7 - 14.5)	0.0004
R3	16.7	75.0	58.3	(48.4 - 68.3)	0.0002	96.2	93.4	-2.7	(-6.6 - 1.1)	0.2586
R4	40.7	83.3	42.6	(31.4 - 53.6)	0.0002	90.7	92.3	1.6	(-2.8 - 6.4)	0.6684
R5	27.8	80.6	52.8	(43.2 - 62.5)	0.0002	97.8	95.1	-2.7	(-6.7 - 1.1)	0.2650
R6	63.9	73.1	9.3	(0.0 - 18.7)	0.0748	92.9	96.7	3.8	(1.1 - 7.3)	0.0438
R7	64.8	89.8	25.0	(17.1 - 33.9)	0.0002	90.2	95.1	4.9	(1.1 - 8.8)	0.0252
R8	71.3	93.5	22.2	(13.3 - 30.7)	0.0002	88.0	96.2	8.2	(4.1 - 12.6)	0.0002
R9	63.0	82.4	19.4	(12.2 - 28.2)	0.0002	95.1	97.3	2.2	(-0.6 - 5.3)	0.2984
R10	42.6	70.4	27.8	(16.7 - 38.5)	0.0002	87.4	92.9	5.5	(1.1 - 10.2)	0.0452
R11	59.3	83.3	24.1	(15.8 - 33.3)	0.0002	82.5	91.3	8.7	(3.5 - 13.7)	0.0026
Mean S	62.3	77.9	15.6	(12.2 - 18.8)	0.0002	94.0	96.1	2.1	(1.0 - 3.2)	0.0002
Mean G	62.2	77.8	15.6	(12.5 - 18.6)	0.0002	88.7	91.3	2.5	(1.0 - 4.1)	0.0012
Mean R	51.1	81.5	30.4	(26.1 - 34.4)	0.0002	90.3	94.9	4.6	(3.0 - 6.2)	0.0002
Mean	58.5	79.0	20.5	(17.8 - 23.4)	0.0002	91.0	94.1	3.1	(2.1 - 4.1)	0.0002

(b) Reader (noncontrast CT) vs. Reader+PANDA assistance (noncontrast CT) by the evaluation of sensitivity and specificity for PDAC identification (PDAC vs. nonPDAC + normal).

Supplementary Table 7: The impact of PANDA assistance on reader performance on noncontrast CT by sensitivity and specificity. Two-sided permutation tests were used to compute the statistical difference. S, pancreas specialist; G, general radiologist; R, radiology resident; Sens, sensitivity; Sens-A, sensitivity with PANDA assistance; Spec, specificity; Spec-A, specificity with PANDA assistance.

			Lesion detection					
**Reader**	**Acc**	**Δ**	**95% CI**	**p-value**	**BAcc**	**Δ**	**95% CI**	**p-value**
PANDA	96.9	-	-	-	97.4	-	-	-
S1	78.4	18.6	(13.7 - 23.0)	0.0002	82.0	15.4	(11.9 - 18.9)	0.0002
S2	84.5	12.4	(7.9 - 16.8)	0.0002	85.1	12.3	(8.1 - 16.7)	0.0002
S3	90.7	6.2	(3.4 - 9.3)	0.0002	91.1	6.3	(3.5 - 9.5)	0.0002
S4	92.1	4.8	(2.4 - 7.2)	0.0002	93.1	4.3	(2.1 - 6.5)	0.0002
S5	89.3	7.6	(4.1 - 11.0)	0.0002	91.1	6.3	(3.6 - 9.1)	0.0002
S6	91.1	5.8	(3.1 - 8.9)	0.0002	92.3	5.1	(2.6 - 7.8)	0.0002
S7	89.0	7.9	(4.8 - 11.3)	0.0002	90.1	7.3	(4.4 - 10.5)	0.0002
S8	86.9	10.0	(6.2 - 13.4)	0.0002	89.1	8.3	(5.2 - 11.1)	0.0002
S9	89.3	7.6	(4.5 - 11.0)	0.0002	91.1	6.3	(3.7 - 8.9)	0.0002
S10	86.6	10.3	(6.9 - 13.7)	0.0002	88.7	8.7	(6.0 - 11.4)	0.0002
S11	89.7	7.2	(3.8 - 10.7)	0.0002	90.4	7.0	(3.6 - 10.0)	0.0002
G1	81.4	15.5	(11.3 - 19.6)	0.0002	80.2	17.2	(12.8 - 21.9)	0.0002
G2	77.0	19.9	(14.8 - 25.1)	0.0002	72.3	25.1	(20.3 - 30.0)	0.0002
G3	85.6	11.3	(7.9 - 15.1)	0.0002	87.7	9.7	(7.0 - 12.8)	0.0002
G4	85.9	11.0	(7.2 - 15.1)	0.0002	87.1	10.3	(6.9 - 13.9)	0.0002
G5	85.2	11.7	(8.2 - 15.5)	0.0002	87.0	10.4	(7.3 - 13.9)	0.0002
G6	83.2	13.7	(9.6 - 18.2)	0.0002	84.3	13.2	(9.3 - 17.5)	0.0002
G7	88.3	8.6	(5.2 - 12.0)	0.0002	89.6	7.9	(4.6 - 11.1)	0.0002
G8	86.9	10.0	(6.2 - 14.1)	0.0002	87.8	9.6	(6.1 - 13.3)	0.0002
G9	88.7	8.2	(4.8 - 11.7)	0.0002	89.0	8.5	(5.0 - 11.8)	0.0002
G10	87.3	9.6	(6.2 - 13.4)	0.0002	88.1	9.3	(6.0 - 12.8)	0.0002
G11	87.6	9.3	(5.8 - 13.1)	0.0002	89.7	7.7	(4.9 - 10.7)	0.0002
R1	75.9	21.0	(16.2 - 25.8)	0.0002	80.0	17.4	(13.8 - 20.9)	0.0002
R2	91.8	5.2	(1.7 - 8.6)	0.0036	91.7	5.7	(2.5 - 9.1)	0.0006
R3	80.1	16.8	(12.7 - 21.3)	0.0002	80.8	16.6	(12.5 - 21.2)	0.0002
R4	83.8	13.1	(8.9 - 17.2)	0.0002	86.0	11.4	(7.8 - 15.1)	0.0002
R5	80.1	16.8	(12.4 - 21.6)	0.0002	81.8	15.6	(11.6 - 20.0)	0.0002
R6	84.5	12.4	(8.6 - 16.2)	0.0002	85.3	12.2	(8.2 - 16.2)	0.0002
R7	83.5	13.4	(9.6 - 17.5)	0.0002	85.8	11.6	(8.2 - 15.1)	0.0002
R8	85.9	11.0	(7.6 - 14.8)	0.0002	88.1	9.3	(6.4 - 12.5)	0.0002
R9	87.6	9.3	(6.2 - 12.7)	0.0002	89.6	7.9	(5.3 - 10.8)	0.0002
R10	77.7	19.2	(14.8 - 24.1)	0.0002	80.6	16.9	(13.1 - 20.9)	0.0002
R11	81.4	15.5	(11.0 - 20.3)	0.0002	84.1	13.3	(9.2 - 17.0)	0.0002
Mean S	88.0	8.9	(6.4 - 11.6)	0.0002	89.5	7.9	(5.9 - 9.9)	0.0002
Mean G	85.2	11.7	(9.2 - 14.3)	0.0002	85.7	11.7	(9.5 - 14.0)	0.0002
Mean R	82.9	14.0	(11.2 - 16.9)	0.0002	84.9	12.5	(10.2 - 14.9)	0.0002
Mean	85.4	11.5	(9.1 - 14.1)	0.0002	86.7	10.7	(8.6 - 12.9)	0.0002

(a) Reader (noncontrast CT) vs. PANDA (noncontrast CT) by the evaluation of accuracy and balanced accuracy for lesion detection (PDAC+non-PDAC vs. normal)

			PDAC identification					
**Reader**	**Acc**	**Δ**	**95% CI**	**p-value**	**BAcc**	**Δ**	**95% CI**	**p-value**
PANDA	95.5	-	-	-	94.9	-	-	-
S1	72.5	23.0	(17.9 - 28.2)	0.0002	66.9	28.0	(22.3 - 33.8)	0.0002
S2	80.1	15.5	(10.0 - 20.3)	0.0002	78.3	16.7	(10.7 - 22.2)	0.0002
S3	80.1	15.5	(11.0 - 19.9)	0.0002	75.4	19.5	(14.5 - 24.6)	0.0002
S4	91.1	4.5	(1.0 - 7.9)	0.0188	89.9	5.1	(1.2 - 9.1)	0.0160
S5	85.6	10.0	(6.2 - 13.7)	0.0002	81.3	13.6	(8.9 - 18.1)	0.0002
S6	87.6	7.9	(4.5 - 11.7)	0.0002	85.0	9.9	(5.5 - 14.5)	0.0002
S7	84.2	11.3	(7.2 - 16.2)	0.0002	81.2	13.8	(9.0 - 19.0)	0.0002
S8	84.9	10.7	(6.9 - 14.4)	0.0002	80.4	14.5	(10.3 - 19.1)	0.0002
S9	80.8	14.8	(10.6 - 19.3)	0.0002	75.4	19.5	(14.8 - 24.3)	0.0002
S10	80.1	15.5	(11.0 - 20.3)	0.0002	74.3	20.6	(15.2 - 25.9)	0.0002
S11	78.0	17.5	(13.1 - 22.3)	0.0002	71.7	23.2	(18.3 - 28.1)	0.0002
G1	68.4	27.1	(21.6 - 32.6)	0.0002	68.2	26.7	(20.7 - 32.6)	0.0002
G2	80.8	14.8	(10.3 - 19.2)	0.0002	77.7	17.3	(12.6 - 21.9)	0.0002
G3	81.4	14.1	(10.3 - 18.6)	0.0002	77.8	17.1	(12.7 - 22.3)	0.0002
G4	76.3	19.2	(13.7 - 24.1)	0.0002	73.4	21.6	(15.4 - 27.1)	0.0002
G5	75.3	20.3	(15.8 - 25.1)	0.0002	68.9	26.0	(20.9 - 31.0)	0.0002
G6	76.6	18.9	(13.7 - 24.1)	0.0002	75.7	19.2	(13.8 - 25.0)	0.0002
G7	84.9	10.7	(6.5 - 14.8)	0.0002	81.5	13.4	(8.9 - 18.1)	0.0002
G8	81.8	13.7	(8.9 - 18.6)	0.0002	77.6	17.4	(11.8 - 22.8)	0.0002
G9	83.2	12.4	(7.9 - 17.2)	0.0002	80.5	14.4	(9.1 - 19.6)	0.0004
G10	80.1	15.5	(11.0 - 20.3)	0.0002	76.0	18.9	(13.7 - 24.4)	0.0002
G11	79.0	16.5	(11.7 - 21.0)	0.0002	72.7	22.2	(16.9 - 27.6)	0.0002
R1	67.0	28.5	(23.7 - 33.7)	0.0002	60.5	34.4	(29.1 - 39.9)	0.0002
R2	82.8	12.7	(8.6 - 16.8)	0.0002	81.6	13.3	(8.7 - 18.1)	0.0002
R3	66.7	28.9	(23.4 - 34.0)	0.0002	56.4	38.5	(34.1 - 42.7)	0.0002
R4	72.2	23.4	(17.5 - 29.2)	0.0002	65.7	29.2	(22.9 - 35.3)	0.0002
R5	71.8	23.7	(18.2 - 28.9)	0.0002	62.8	32.1	(26.7 - 37.3)	0.0002
R6	82.1	13.4	(9.3 - 17.5)	0.0002	78.4	16.5	(11.6 - 21.4)	0.0002
R7	80.8	14.8	(10.3 - 19.3)	0.0002	77.5	17.4	(12.6 - 22.3)	0.0002
R8	81.8	13.7	(8.9 - 18.2)	0.0002	79.6	15.3	(9.7 - 20.5)	0.0002
R9	83.2	12.4	(8.2 - 16.5)	0.0002	79.0	15.9	(11.1 - 20.6)	0.0002
R10	70.8	24.7	(19.9 - 30.2)	0.0002	65.0	29.9	(24.5 - 35.8)	0.0002
R11	73.9	21.6	(16.5 - 26.8)	0.0002	70.9	24.0	(18.2 - 29.9)	0.0002
Mean S	82.3	13.3	(10.6 - 16.2)	0.0002	78.2	16.8	(13.9 - 19.6)	0.0002
Mean G	78.9	16.7	(13.6 - 19.8)	0.0002	75.5	19.5	(16.3 - 22.5)	0.0002
Mean R	75.7	19.8	(16.6 - 22.8)	0.0002	70.7	24.3	(21.6 - 26.9)	0.0002
Mean	79.0	16.6	(13.8 - 19.4)	0.0002	74.8	20.2	(17.6 - 22.7)	0.0002

(b) Reader (noncontrast CT) vs. PANDA (noncontrast CT) by the evaluation of accuracy and balanced accuracy for PDAC identification (PDAC vs. nonPDAC + normal).

Supplementary Table 8: Reader (noncontrast CT) vs. PANDA (noncontrast CT) by accuracy and balanced accuracy. Two-sided permutation tests were used to compute the statistical difference. S, pancreas specialist; G, general radiologist; R, radiology resident; Acc, accuracy; BAcc, balanced accuracy.

Lesion detection										
**Reader**	**Acc**	**Acc-A**	**Δ**	**95% CI**	**p-value**	**BAcc**	**BAcc-A**	**Δ**	**95% CI**	**p-value**
S1	78.4	92.8	14.4	(10.3 - 18.6)	0.0002	82.0	94.0	12.0	(8.9 - 15.5)	0.0002
S2	84.5	90.7	6.2	(0.7 - 11.0)	0.0288	85.1	92.0	6.9	(1.8 - 11.3)	0.0056
S3	90.7	95.2	4.5	(2.1 - 6.9)	0.0012	91.1	95.9	4.7	(2.3 - 7.5)	0.0008
S4	92.1	95.9	3.8	(1.4 - 6.2)	0.0072	93.1	96.3	3.1	(0.9 - 5.5)	0.0072
S5	89.3	94.2	4.8	(2.7 - 7.2)	0.0006	91.1	95.1	4.0	(2.2 - 6.0)	0.0006
S6	91.1	94.8	3.8	(1.4 - 6.5)	0.0070	92.3	95.7	3.4	(1.4 - 5.9)	0.0062
S7	89.0	94.8	5.8	(2.1 - 9.6)	0.0026	90.1	95.1	5.0	(1.6 - 8.8)	0.0086
S8	86.9	90.0	3.1	(1.4 - 5.2)	0.0034	89.1	91.7	2.6	(1.1 - 4.2)	0.0034
S9	89.3	94.8	5.5	(3.1 - 8.3)	0.0002	91.1	95.7	4.6	(2.5 - 7.0)	0.0002
S10	86.6	90.7	4.1	(2.1 - 6.5)	0.0010	88.7	92.3	3.6	(1.8 - 5.5)	0.0010
S11	89.7	90.7	1.0	(-0.3 - 2.4)	0.3624	90.4	91.3	0.9	(-0.6 - 2.3)	0.3624
G1	81.4	86.3	4.8	(-1.0 - 10.0)	0.0942	80.2	88.3	8.1	(3.0 - 13.2)	0.0024
G2	77.0	89.3	12.4	(7.2 - 18.2)	0.0004	72.3	90.6	18.3	(13.1 - 23.8)	0.0002
G3	85.6	87.6	2.1	(-1.4 - 5.5)	0.3688	87.7	89.7	2.0	(-1.0 - 5.2)	0.2558
G4	85.9	95.2	9.3	(5.8 - 13.1)	0.0002	87.1	95.9	8.7	(5.4 - 12.3)	0.0002
G5	85.2	93.1	7.9	(4.1 - 11.3)	0.0002	87.0	94.3	7.3	(4.0 - 10.3)	0.0002
G6	83.2	92.4	9.3	(5.5 - 13.7)	0.0002	84.3	92.8	8.6	(4.7 - 13.0)	0.0002
G7	88.3	92.4	4.1	(1.7 - 6.9)	0.0036	89.6	93.6	4.0	(1.6 - 6.6)	0.0016
G8	86.9	93.1	6.2	(2.1 - 10.0)	0.0042	87.8	94.0	6.2	(2.4 - 10.0)	0.0014
G9	88.7	94.5	5.8	(2.4 - 9.6)	0.0022	89.0	94.7	5.7	(2.2 - 9.6)	0.0030
G10	87.3	92.1	4.8	(1.7 - 7.9)	0.0030	88.1	93.0	4.9	(1.9 - 8.0)	0.0026
G11	87.6	92.8	5.2	(2.4 - 7.9)	0.0006	89.7	93.9	4.1	(1.9 - 6.6)	0.0006
R1	75.9	96.9	21.0	(16.5 - 25.8)	0.0002	80.0	97.4	17.4	(13.9 - 21.1)	0.0002
R2	91.8	92.4	0.7	(-2.4 - 3.4)	0.8314	91.7	93.7	2.0	(-1.0 - 5.2)	0.1900
R3	80.1	91.4	11.3	(6.5 - 16.2)	0.0002	80.8	92.3	11.5	(6.6 - 16.5)	0.0002
R4	83.8	95.5	11.7	(7.9 - 15.8)	0.0002	86.0	96.3	10.3	(7.0 - 14.1)	0.0002
R5	80.1	93.1	13.1	(8.9 - 17.5)	0.0002	81.8	94.0	12.2	(8.3 - 16.3)	0.0002
R6	84.5	92.1	7.6	(3.4 - 11.7)	0.0004	85.3	92.8	7.6	(3.5 - 11.6)	0.0002
R7	83.5	92.1	8.6	(5.2 - 12.0)	0.0002	85.8	93.4	7.6	(4.6 - 10.8)	0.0002
R8	85.9	96.2	10.3	(6.9 - 14.1)	0.0002	88.1	96.9	8.7	(5.7 - 12.0)	0.0002
R9	87.6	91.8	4.1	(0.7 - 7.2)	0.0186	89.6	93.0	3.4	(0.5 - 6.3)	0.0184
R10	77.7	89.7	12.0	(7.2 - 16.8)	0.0002	80.6	91.1	10.6	(6.0 - 14.9)	0.0002
R11	81.4	89.3	7.9	(4.5 - 11.7)	0.0002	84.1	90.7	6.6	(3.6 - 9.9)	0.0002
Mean S	88.0	93.2	5.2	(4.0 - 6.4)	0.0002	89.5	94.1	4.6	(3.6 - 5.7)	0.0002
Mean G	85.2	91.7	6.5	(5.0 - 8.1)	0.0002	85.7	92.8	7.1	(5.6 - 8.7)	0.0002
Mean R	82.9	92.8	9.8	(8.0 - 11.8)	0.0002	84.9	93.8	8.9	(7.3 - 10.6)	0.0002
Mean	85.4	92.6	7.2	(6.0 - 8.4)	0.0002	86.7	93.6	6.9	(5.8 - 8.0)	0.0002

(a) Reader (noncontrast CT) vs. Reader+PANDA assistance (noncontrast CT) by the evaluation of accuracy and balanced accuracy for lesion detection (PDAC+non-PDAC vs. normal)

PDAC identification										
**Reader**	**Acc**	**Acc-A**	**Δ**	**95% CI**	**p-value**	**BAcc**	**BAcc-A**	**Δ**	**95% CI**	**p-value**
S1	72.5	89.0	16.5	(11.3 - 21.6)	0.0002	66.9	87.1	20.1	(14.5 - 26.0)	0.0002
S2	80.1	85.2	5.2	(-0.7 - 10.7)	0.1060	78.3	82.2	3.9	(-2.9 - 10.3)	0.2666
S3	80.1	90.0	10.0	(6.2 - 13.7)	0.0002	75.4	88.3	12.9	(8.5 - 17.0)	0.0002
S4	91.1	93.8	2.7	(-0.3 - 6.2)	0.1492	89.9	93.9	4.1	(0.4 - 7.8)	0.0396
S5	85.6	91.1	5.5	(2.7 - 8.3)	0.0004	81.3	88.7	7.4	(3.9 - 11.1)	0.0004
S6	87.6	93.8	6.2	(2.7 - 10.0)	0.0010	85.0	92.4	7.4	(3.6 - 11.5)	0.0008
S7	84.2	93.5	9.3	(4.5 - 13.7)	0.0002	81.2	92.9	11.7	(6.6 - 17.1)	0.0002
S8	84.9	87.6	2.7	(1.0 - 4.8)	0.0070	80.4	84.1	3.7	(1.5 - 6.2)	0.0070
S9	80.8	91.4	10.7	(6.5 - 14.8)	0.0002	75.4	89.0	13.6	(8.8 - 18.2)	0.0002
S10	80.1	86.6	6.5	(3.8 - 9.6)	0.0002	74.3	82.7	8.4	(5.0 - 11.9)	0.0002
S11	78.0	80.8	2.7	(0.7 - 4.8)	0.0250	71.7	75.6	3.9	(1.5 - 6.9)	0.0044
G1	68.4	81.8	13.4	(6.9 - 20.3)	0.0002	68.2	77.7	9.5	(2.3 - 16.2)	0.0094
G2	80.8	73.2	-7.6	(-12.7 - -2.1)	0.0080	77.7	70.9	-6.8	(-12.3 - -1.2)	0.0174
G3	81.4	88.7	7.2	(3.4 - 11.0)	0.0014	77.8	85.7	7.8	(3.6 - 12.3)	0.0014
G4	76.3	82.8	6.5	(1.4 - 11.7)	0.0212	73.4	84.4	11.1	(6.1 - 16.2)	0.0002
G5	75.3	89.0	13.7	(9.3 - 18.2)	0.0002	68.9	85.9	17.0	(11.8 - 22.0)	0.0002
G6	76.6	88.7	12.0	(7.2 - 16.8)	0.0002	75.7	88.7	13.0	(8.3 - 17.9)	0.0002
G7	84.9	87.6	2.7	(0.3 - 5.2)	0.0742	81.5	85.0	3.5	(0.8 - 6.4)	0.0282
G8	81.8	92.1	10.3	(5.8 - 14.8)	0.0002	77.6	91.1	13.5	(8.3 - 18.7)	0.0002
G9	83.2	91.4	8.2	(3.8 - 12.7)	0.0008	80.5	91.8	11.3	(6.4 - 16.3)	0.0002
G10	80.1	83.8	3.8	(0.3 - 7.2)	0.0664	76.0	80.9	4.9	(1.0 - 9.1)	0.0294
G11	79.0	89.7	10.7	(6.2 - 15.5)	0.0002	72.7	87.4	14.7	(9.6 - 20.1)	0.0002
R1	67.0	94.8	27.8	(22.3 - 33.3)	0.0002	60.5	94.0	33.5	(28.0 - 38.9)	0.0002
R2	82.8	88.0	5.2	(1.0 - 9.3)	0.0270	81.6	85.1	3.5	(-1.2 - 8.2)	0.1524
R3	66.7	86.6	19.9	(14.4 - 25.4)	0.0002	56.4	84.2	27.8	(22.6 - 32.9)	0.0002
R4	72.2	89.0	16.8	(11.3 - 22.7)	0.0002	65.7	87.8	22.1	(16.1 - 28.4)	0.0002
R5	71.8	89.7	17.9	(12.7 - 23.0)	0.0002	62.8	87.8	25.0	(19.8 - 30.2)	0.0002
R6	82.1	88.0	5.8	(2.1 - 10.3)	0.0060	78.4	84.9	6.5	(1.6 - 11.8)	0.0088
R7	80.8	93.1	12.4	(8.6 - 16.5)	0.0002	77.5	92.4	15.0	(10.5 - 19.7)	0.0002
R8	81.8	95.2	13.4	(8.9 - 17.9)	0.0002	79.6	94.8	15.2	(10.1 - 20.1)	0.0002
R9	83.2	91.8	8.6	(4.8 - 12.7)	0.0002	79.0	89.8	10.8	(6.5 - 15.3)	0.0002
R10	70.8	84.5	13.7	(8.6 - 18.6)	0.0002	65.0	81.6	16.6	(10.9 - 22.6)	0.0002
R11	73.9	88.3	14.4	(10.0 - 18.9)	0.0002	70.9	87.3	16.4	(11.4 - 21.8)	0.0002
Mean S	82.3	89.3	7.1	(5.4 - 8.7)	0.0002	78.2	87.0	8.8	(7.1 - 10.5)	0.0002
Mean G	78.9	86.3	7.4	(5.7 - 9.1)	0.0002	75.5	84.5	9.1	(7.3 - 10.8)	0.0002
Mean R	75.7	89.9	14.2	(11.9 - 16.6)	0.0002	70.7	88.2	17.5	(15.2 - 19.7)	0.0002
Mean	79.0	88.5	9.5	(8.0 - 11.1)	0.0002	74.8	86.6	11.8	(10.3 - 13.3)	0.0002

(b) Reader (noncontrast CT) vs. Reader+PANDA assistance (noncontrast CT) by the evaluation of accuracy and balanced accuracy for PDAC identification (PDAC vs. nonPDAC+normal).

Supplementary Table 9: The impact of PANDA assistance on reader performance on noncontrast CT by accuracy and balanced accuracy. Two-sided permutation tests were used to compute the statistical difference. S, pancreas specialist; G, general radiologist; R, radiology resident; Acc, accuracy; Acc-A, accuracy with PANDA assistance; BAcc, balanced accuracy; BAcc-A, balanced accuracy with PANDA assistance.

				Lesion detection				
**Reader**	**Sens**	**Δ**	**95% CI**	**p-value**	**Spec**	**Δ**	**95% CI**	**p-value**
PANDA	94.9	-	-	-	100	-	-	-
S12	92.0	2.9	(-1.7 - 7.4)	0.3388	98.3	1.7	(0.0 - 4.3)	0.5140
S13	90.3	4.6	(0.0 - 9.2)	0.1030	100	0.0	(0.0 - 0.0)	1.0000
S14	85.7	9.1	(3.8 - 15.0)	0.0004	99.1	0.9	(0.0 - 2.7)	0.9960
S15	92.6	2.3	(-1.1 - 5.9)	0.3506	100	0.0	(0.0 - 0.0)	1.0000
S16	92.0	2.9	(-1.2 - 6.9)	0.2620	98.3	1.7	(0.0 - 4.8)	0.4938
S17	95.4	-0.6	(-4.7 - 3.4)	1.0000	98.3	1.7	(0.0 - 4.6)	0.4916
S18	92.0	2.9	(-1.2 - 6.7)	0.2686	100	0.0	(0.0 - 0.0)	1.0000
S19	91.4	3.4	(-0.6 - 7.8)	0.1516	99.1	0.9	(0.0 - 3.2)	0.9982
S20	95.4	-0.6	(-3.4 - 2.3)	0.9832	97.4	2.6	(0.0 - 5.8)	0.2630
S21	85.7	9.1	(4.2 - 14.9)	0.0016	98.3	1.7	(0.0 - 4.5)	0.4938
S22	89.1	5.7	(1.1 - 11.0)	0.0438	98.3	1.7	(0.0 - 4.5)	0.4986
S23	93.1	1.7	(-2.3 - 6.1)	0.5740	96.6	3.4	(0.8 - 7.1)	0.1194
S24	91.4	3.4	(-0.6 - 8.1)	0.2032	96.6	3.4	(0.8 - 6.9)	0.1204
S25	96.0	-1.1	(-5.5 - 2.9)	0.7820	88.8	11.2	(5.6 - 17.5)	0.0002
S26	97.1	-2.3	(-6.2 - 1.2)	0.3310	99.1	0.9	(0.0 - 2.9)	0.9970
Mean	92.0	2.9	(0.1 - 5.8)	0.0874	97.9	2.1	(1.4 - 3.0)	0.0002

(a) Reader (contrast-enhanced CT) vs. PANDA (noncontrast CT) by the evaluation of sensitivity and specificity for lesion detection (PDAC+nonPDAC vs. normal)

				PDAC identification				
**Reader**	**Sens**	**Δ**	**95% CI**	**p-value**	**Spec**	**Δ**	**95% CI**	**p-value**
PANDA	92.6	-	-	-	97.3	-	-	-
S12	74.1	18.5	(11.0 - 26.9)	0.0002	98.4	-1.1	(-3.9 - 1.8)	0.7230
S13	79.6	13.0	(4.6 - 22.0)	0.0076	98.9	-1.6	(-3.9 - 0.5)	0.3886
S14	74.1	18.5	(10.5 - 27.0)	0.0002	93.4	3.8	(0.0 - 7.9)	0.1194
S15	83.3	9.3	(2.7 - 17.0)	0.0236	99.5	-2.2	(-4.7 - 0.0)	0.2290
S16	76.9	15.7	(8.5 - 23.7)	0.0004	95.6	1.6	(-1.2 - 4.8)	0.5072
S17	69.4	23.1	(14.8 - 31.0)	0.0002	97.3	0.0	(-3.2 - 3.2)	1.0000
S18	81.5	11.1	(3.8 - 19.0)	0.0082	98.4	-1.1	(-3.6 - 1.6)	0.6952
S19	79.6	13.0	(6.2 - 19.7)	0.0008	97.8	-0.5	(-3.2 - 2.2)	1.0000
S20	94.4	-1.9	(-7.3 - 4.3)	0.7450	92.3	4.9	(1.1 - 8.9)	0.0400
S21	63.9	28.7	(19.3 - 38.7)	0.0002	100	-2.7	(-5.1 - -0.6)	0.0696
S22	75.9	16.7	(8.9 - 24.7)	0.0002	98.4	-1.1	(-3.6 - 1.2)	0.6912
S23	80.6	12.0	(4.3 - 20.2)	0.0066	98.9	-1.6	(-4.0 - 0.5)	0.3750
S24	77.8	14.8	(7.4 - 22.5)	0.0022	93.4	3.8	(1.0 - 6.9)	0.0370
S25	88.9	3.7	(-2.3 - 9.8)	0.3920	91.8	5.5	(1.6 - 9.6)	0.0178
S26	93.5	-0.9	(-6.4 - 3.8)	1.0000	96.7	0.5	(-2.7 - 3.7)	0.9960
Mean	79.6	13.0	(8.5 - 17.8)	0.0002	96.7	0.5	(-0.7 - 1.9)	0.6772

(b) Reader(contrast-enhanced CT) vs. PANDA (noncontrast CT) by the evaluation of sensitivity and specificity for PDAC identification (PDAC vs. nonPDAC+normal).

Supplementary Table 10: Reader(contrast-enhanced CT) vs. PANDA (noncontrast CT) by sensitivity and specificity. Two-sided permutation tests were used to compute the statistical difference. S, pancreas specialist; Sens, sensitivity; Spec, specificity.

Lesion detection								
**Reader**	**Acc**	**Δ**	**95% CI**	**p-value**	**BAcc**	**Δ**	**95% CI**	**p-value**
PANDA	96.9	-	-	-	97.4	-	-	-
S12	94.5	2.4	(-0.3 - 5.2)	0.1788	95.1	2.3	(-0.2 - 4.8)	0.1188
S13	94.2	2.7	(0.0 - 5.5)	0.1030	95.1	2.3	(0.0 - 4.6)	0.1030
S14	91.1	5.8	(2.4 - 9.3)	0.0002	92.4	5.0	(2.1 - 7.8)	0.0002
S15	95.5	1.4	(-0.7 - 3.4)	0.3506	96.3	1.1	(-0.6 - 2.9)	0.3506
S16	94.5	2.4	(-0.0 - 5.2)	0.1162	95.1	2.3	(0.0 - 4.7)	0.0796
S17	96.6	0.3	(-2.4 - 3.1)	1.0000	96.9	0.6	(-1.7 - 2.9)	0.6588
S18	95.2	1.7	(-0.7 - 4.1)	0.2686	96.0	1.4	(-0.6 - 3.4)	0.2686
S19	94.5	2.4	(0.0 - 5.2)	0.0944	95.3	2.1	(0.2 - 4.4)	0.0786
S20	96.2	0.7	(-1.4 - 2.7)	0.7744	96.4	1.0	(-0.8 - 3.2)	0.3802
S21	90.7	6.2	(3.1 - 10.0)	0.0004	92.0	5.4	(2.6 - 8.6)	0.0004
S22	92.8	4.1	(1.0 - 7.2)	0.0174	93.7	3.7	(1.0 - 6.5)	0.0124
S23	94.5	2.4	(-0.3 - 5.5)	0.1516	94.8	2.6	(0.0 - 5.3)	0.0632
S24	93.5	3.4	(0.3 - 6.5)	0.0410	94.0	3.4	(0.7 - 6.2)	0.0180
S25	93.1	3.8	(0.3 - 7.2)	0.0576	92.4	5.0	(1.3 - 8.4)	0.0082
S26	97.9	-1.0	(-3.4 - 1.0)	0.5404	98.1	-0.7	(-2.8 - 1.2)	0.5404
Mean	94.3	2.6	(0.8 - 4.5)	0.0002	94.9	2.5	(1.0 - 4.1)	0.0002

(a) Reader (contrast-enhanced CT) vs. PANDA (noncontrast CT) by the evaluation of accuracy and balanced accuracy for lesion detection (PDAC+non-PDAC vs. normal)

PDAC identification								
**Reader**	**Acc**	**Δ**	**95% CI**	**p-value**	**BAcc**	**Δ**	**95% CI**	**p-value**
PANDA	95.5	-	-	-	94.9	-	-	-
S12	89.3	6.2	(2.7 - 10.0)	0.0018	86.2	8.7	(4.8 - 13.1)	0.0002
S13	91.8	3.8	(0.0 - 7.6)	0.0608	89.3	5.7	(0.9 - 10.4)	0.0140
S14	86.3	9.3	(5.2 - 13.4)	0.0002	83.8	11.2	(6.5 - 15.8)	0.0002
S15	93.5	2.1	(-0.7 - 5.2)	0.2820	91.4	3.5	(-0.1 - 7.5)	0.0796
S16	88.7	6.9	(3.4 - 10.7)	0.0008	86.2	8.7	(4.7 - 13.0)	0.0002
S17	86.9	8.6	(4.5 - 12.4)	0.0001	83.4	11.6	(7.0 - 15.9)	0.0002
S18	92.1	3.4	(0.3 - 6.9)	0.0624	89.9	5.0	(1.1 - 9.3)	0.0140
S19	91.1	4.5	(1.4 - 7.6)	0.0114	88.7	6.2	(2.6 - 9.9)	0.0006
S20	93.1	2.4	(-0.7 - 5.8)	0.2382	93.4	1.5	(-1.8 - 5.2)	0.4212
S21	86.6	8.9	(4.8 - 13.1)	0.0002	81.9	13.0	(8.1 - 18.0)	0.0002
S22	90.0	5.5	(2.4 - 8.9)	0.0030	87.1	7.8	(3.9 - 11.9)	0.0004
S23	92.1	3.4	(0.0 - 6.9)	0.0766	89.7	5.2	(1.2 - 9.4)	0.0202
S24	87.6	7.9	(4.5 - 11.3)	0.0002	85.6	9.3	(5.1 - 13.3)	0.0002
S25	90.7	4.8	(1.7 - 8.2)	0.0104	90.3	4.6	(1.1 - 8.3)	0.0156
S26	95.5	0.0	(-2.7 - 2.7)	1.0000	95.1	-0.2	(-3.3 - 3.0)	0.9866
Mean	90.4	5.2	(3.1 - 7.3)	0.0002	88.1	6.8	(4.4 - 9.2)	0.0002

(b) Reader (contrast-enhanced CT) vs. PANDA (noncontrast CT) by the evaluation of accuracy and balanced accuracy for PDAC identification (PDAC vs. nonPDAC + normal).

Supplementary Table 11: Reader (contrast-enhanced CT) vs. PANDA (noncontrast CT) by accuracy and balanced accuracy. Two-sided permutation tests were used to compute the statistical difference. S, pancreas specialist; acc, accuracy; bal. acc, balanced accuracy.

Site	A (SHCMU)	B (FAHZU)	C (XH)	D (FUSCC)
Patients	1274	1506	176	254
Accuracy(%)	81.3	79.2	73.9	79.9
Balanced accuracy(%)	46.7	55.8	45.7	60.8

Supplementary Table 12: Differential diagnosis results on four external centers with pathologically confirmed PDAC and nonPDAC.

	AUC	CI	Sensitivity (%) (Main or mixed-duct IPMN)	CI	Specificity (%) (Branch-duct IPMN)	CI
Internal All (n=87)	0.944	0.894-0.982	94.1	87.2-100	80.6	66.7-93.1
Internal Correct (n=71)	0.948	0.892-0.988	95.5	88.9-100	77.8	60.6-92.3
External All (n=172)	0.915	0.867-0.958	89.0	81.6-95.2	81.1	73.0-89.5
External Correct (n=139)	0.947	0.908-0.979	94.1	88.3-98.7	80.3	70.7-88.9

Supplementary Table 13: Results for IPMN subtype classification. We report two sets of results separately on the both internal differential diagnosis cohort and external multicenter cohort, i.e., AUC, sensitivity, and specificity on all collected IPMN (denoted as "All"), and on those correctly classified by PANDA Stage-3 (denoted as "Correct"), respectively.

False positive type	RW1 (number of cases)	RW2 (number of cases)	Category
Pancreatic fatty infiltration[7]	33	0	Easy to rule out by radiologists
Stomach/bowel contents	31	2	
Heart	6	0	
Motion artifacts	0	2	
Abdominal structure	2	0	Requires time or follow-up
Low-density area without clinical meaning	4	0	

Supplementary Table 14: Analysis of PANDA's false positive predictions in real-world evaluation RW1 and RW2. The low-density area without clinical meaning is diagnosed by MDT and confirmed by follow-up.

References

[1] Tanaka, M., Fernández-del Castillo, C., Kamisawa, T., Jang, J.Y., Levy, P., Ohtsuka, T., Salvia, R., Shimizu, Y., Tada, M., Wolfgang, C.L.: Revisions of international consensus fukuoka guidelines for the management of IPMN of the pancreas. Pancreatology **17**(5), 738–753 (2017)

[2] Heinrich, M.P., Jenkinson, M., Brady, M., Schnabel, J.A.: MRF-based deformable registration and ventilation estimation of lung CT. IEEE Transactions on Medical Imaging **32**(7), 1239–1248 (2013)

[3] Yushkevich, P.A., Piven, J., Hazlett, H.C., Smith, R.G., Ho, S., Gee, J.C., Gerig, G.: User-guided 3D active contour segmentation of anatomical structures: significantly improved efficiency and reliability. Neuroimage **31**(3), 1116–1128 (2006)

[4] Yao, J., Cao, K., Hou, Y., Zhou, J., Xia, Y., Nogues, I., Song, Q., Jiang, H., Ye, X., Lu, J., *et al.*: Deep learning for fully automated prediction of overall survival in patients undergoing resection for pancreatic cancer: a retrospective multicenter study. Annals of Surgery **278**(1), 68–79 (2023)

[5] Sahani, D.V., Kadavigere, R., Blake, M., Fernandez-del Castillo, C., Lauwers, G.Y., Hahn, P.F.: Intraductal papillary mucinous neoplasm of pancreas: multi–detector row ct with 2d curved reformations—correlation with mrcp. Radiology **238**(2), 560–569 (2006)

[6] Goggins, M., Overbeek, K.A., Brand, R., Syngal, S., Del Chiaro, M., Bartsch, D.K., Bassi, C., Carrato, A., Farrell, J., Fishman, E.K., *et al.*: Management of patients with increased risk for familial pancreatic cancer: updated recommendations from the international cancer of the pancreas screening (caps) consortium. Gut **69**(1), 7–17 (2020)

[7] Kawamoto, S., Siegelman, S.S., Bluemke, D.A., Hruban, R.H., Fishman, E.K.: Focal fatty infiltration in the head of the pancreas: evaluation with multidetector computed tomography with multiplanar reformation imaging. Journal of Computer Assisted Tomography **33**(1), 90–95 (2009)
