# Supplementary material for: Large-scale pancreatic cancer detection via non-contrast CT and deep learning
Source: Nat Med. 2023 Nov 20;29(12):3033–43. doi: 10.1038/s41591-023-02640-w (PMC10719100; doi:10.1038/s41591-023-02640-w)
Supplement: Supplementary file 2 — Reporting Summary [file 41591_2023_2640_MOESM2_ESM.pdf]

nature portfolio

Corresponding author(s): Chengwei Shao, Yu Shi, Qi Zhang, Tingbo Liang, Ling Zhang, and Jianping Lu

Last updated by author(s): Oct 9, 2023

Reporting Summary

Nature Portfolio wishes to improve the reproducibility of the work that we publish. This form provides structure for consistency and transparency in reporting. For further information on Nature Portfolio policies, see our Editorial Policies and the Editorial Policy Checklist.

Statistics

For all statistical analyses, confirm that the following items are present in the figure legend, table legend, main text, or Methods section.

n/a	Confirmed	
☐	☒	The exact sample size (*n*) for each experimental group/condition, given as a discrete number and unit of measurement
☐	☒	A statement on whether measurements were taken from distinct samples or whether the same sample was measured repeatedly
☐	☒	The statistical test(s) used AND whether they are one- or two-sided<br>*Only common tests should be described solely by name; describe more complex techniques in the Methods section.*
☒	☐	A description of all covariates tested
☒	☐	A description of any assumptions or corrections, such as tests of normality and adjustment for multiple comparisons
☐	☒	A full description of the statistical parameters including central tendency (e.g. means) or other basic estimates (e.g. regression coefficient) AND variation (e.g. standard deviation) or associated estimates of uncertainty (e.g. confidence intervals)
☐	☒	For null hypothesis testing, the test statistic (e.g. *F*, *t*, *r*) with confidence intervals, effect sizes, degrees of freedom and *P* value noted<br>*Give P values as exact values whenever suitable.*
☒	☐	For Bayesian analysis, information on the choice of priors and Markov chain Monte Carlo settings
☒	☐	For hierarchical and complex designs, identification of the appropriate level for tests and full reporting of outcomes
☒	☐	Estimates of effect sizes (e.g. Cohen's *d*, Pearson's *r*), indicating how they were calculated

Our web collection on statistics for biologists contains articles on many of the points above.

Software and code

Policy information about availability of computer code

Data collection	Dicom files were handled with the open source libraries Pydicom (https://pydicom.github.io/, version 2.2.2), SimpleITK (https://simpleitk.org/, version 2.0.2), and NiBabel (https://nipy.org/nibabel/, version 3.2.1). Custom Python (version 3.9.7) script was developed for data de-identification.
Data analysis	The code used for the implementation of PANDA has dependencies on internal tooling and infrastructure, is under patent protection (application numbers: CN 202210575258.9, US 18046405), and thus is not feasible to be publicly released. All experiments and implementation details are described in sufficient detail in the Methods and Supplementary Methods sections to support replication with non-proprietary libraries. Several major components of our work are available in open-source repositories: PyTorch (https://pytorch.org/); nnUNet (https://github.com/MIC-DKFZ/nnUNet). Data analysis was conducted in Python using the numpy (version 1.20.3), scipy (version 1.8.1), and scikit-learn (version 0.24.2) packages. The calculation of people needed to screen in the high-risk population was based on Test for One-Sample Sensitivity and Specificity via PASS software (version 15).

For manuscripts utilizing custom algorithms or software that are central to the research but not yet described in published literature, software must be made available to editors and reviewers. We strongly encourage code deposition in a community repository (e.g. GitHub). See the Nature Portfolio guidelines for submitting code & software for further information.

Data

Policy information about availability of data

All manuscripts must include a data availability statement. This statement should provide the following information, where applicable:

- Accession codes, unique identifiers, or web links for publicly available datasets
- A description of any restrictions on data availability
- For clinical datasets or third party data, please ensure that the statement adheres to our policy

Sample data and interactive demo are displayed in the webpage (http://panda.medofmind.com/). The remaining datasets used in this study are currently not permitted for public release by the respective Institutional Review Boards. Requests for access to aggregate data and supporting clinical documents will be reviewed and approved by an independent review panel on the basis of scientific merit. All data provided are anonymized to respect the privacy of patients who have participated in the studies, in line with applicable laws and regulations. Data requests pertaining to the manuscript may be made to the first author (Kai Cao; mdkaicao163@163.com). Requests will be processed within 6 weeks.

Research involving human participants, their data, or biological material

Policy information about studies with human participants or human data. See also policy information about sex, gender (identity/presentation), and sexual orientation and race, ethnicity and racism.

Reporting on sex and gender	We are using only retrospective data collected through clinical practice. Sex was assigned based on the government-issued ID. The datasets used in the internal training and test cohorts, and the external multi-center test cohorts have sex distributions reported in the paper. Sex-based analysis was not reported because sex was unrelated to model implementation or deployment. Self-identification gender was not collected from the patients.
Reporting on race, ethnicity, or other socially relevant groupings	We are using only retrospective data collected through clinical practice. Race, ethnicity, and other socially relevant groupings were not collected from the patients and were unrelated to model implementation or deployment.
Population characteristics	This retrospective study included five patient cohorts: an internal training cohort , an internal test cohort (together with an additional internal differential diagnosis cohort), an external international multicenter test cohort, a chest noncontrast CT test cohort, and a real-world clinical test cohort. In the first four cohorts, the pancreatic ductal adenocarcinoma (PDAC) and nonPDAC lesions were confirmed by surgical or biopsy histopathology, which we used as the ground truth label for each patient. The normal controls were confirmed without pancreatic or peri-pancreatic disease by a two-year follow-up. Patients with acute pancreatitis and a history of abdominal treatment were excluded.<br><br>The internal training test cohort (normal controls: median age 49 years [IQR 39-60], nonPDAC: median age 55 years [IQR 44-64], PDAC: median age 63 [IQR 55-69]), internal test cohort (normal controls: median age 50 [IQR 38-60], nonPDAC: median age 51 [IQR 43-64], PDAC: median age 64 [IQR 57-70]), internal additional test cohort (nonPDAC: median age 57 [IQR 45-67], PDAC: median age 64 [IQR 56-71]), chest noncontrast CT test cohort (normal controls: median age 37 [IQR 32-45], nonPDAC: median age 59 [IQR 51-65], PDAC: median age 65 [IQR 56-70]), and the real-world clinical test cohort (RW1-normal controls: median age 38 [IQR 33-47], nonPDAC: median age 61 [IQR 51-72], PDAC: median age 66 [IQR 51-72]; RW2-normal controls: median age 58 [IQR 50-68], nonPDAC: median age 48 [IQR 37-64], PDAC: median age 60 [IQR 57-69]) were collected from the internal center in Shanghai, China (Shanghai Institution of Pancreatic Diseases [SIPD]). The external test cohorts were collected from nine centers, of which seven were located in China, one in Taiwan ROC (Linkou Chang Gung Memorial Hospital [CGMH], normal controls: median age 53 [IQR 42-63], PDAC: median age 41 [IQR 51-65] -- Site H), and one in the Czech Republic (General University Hospital in Prague [GUHP], normal controls: median age 73 [IQR 66-78], PDAC: median age 64 [IQR 59-70] -- Site I). The seven centers from China are distributed widely in geographical areas: one in the Northeast (Shengjing Hospital of China Medical University [SHCMU], normal controls: median age 52 [IQR 38-60], nonPDAC: median age 56 [IQR 46-64], PDAC: median age 61 [IQR 55-68] -- Site A), four in the East (First Affiliated Hospital of Zhejiang University [FAHZU], normal controls: median age 57 [IQR 46-66], nonPDAC: median age 56 [IQR 45-66], PDAC: median age 65 [IQR 58-71] -- Site B; Xinhua Hospital [XH], normal controls: median age 46 [IQR 34-60], nonPDAC: median age 60 [IQR 50-67], PDAC: median age 65 [IQR 60-70] --Site C; Fudan University Shanghai Cancer Center [FUSCC], normal controls: median age 49 [IQR 39-55], nonPDAC: median age 54 [IQR 42-62], PDAC: median age 62 [IQR 56-68]-- Site D; and Tianjin Medical University Cancer Institute and Hospital[TMUCIH], PDAC: median age 58 [IQR 51-62]--Site E), and two in the South (Sun Vat-sen University Cancer Center [SYUCC], PDAC: median age 59 [IQR 51-66] -- Site F; and Guangdong Provincial People's Hospital [GPPH], normal controls: median age 60 [IQR 54-66], PDAC: median age 61 [IQR 54-69] -- Site G). For all patients included in the multicenter test cohort, additional metadata for data characteristic was available, including patient age and sex. For the patients with PDAC, the T stage and TNM stage (AJCC eighth edition) and the location of the lesion are available. For example, 707 PDAC patients (25.8%) and 779 PDAC patients (28.5%) in the external test cohorts were TNM stage I cancer and stage II cancer, respectively. Further details are provided in the extended data.
Recruitment	The internal training cohort included 3,208 patients (1,431 PDAC, 140 pancreatic neuroendocrine tumor [PNET], 98 solid pseudopapillary tumor [SPT], 254 intraductal papillary mucinous neoplasm [IPMN], 37 mucinous cystic neoplasm [MCN], 110 chronic pancreatitis [CP], 134 serous cystic neoplasm [SCN], 66 'other', and 938 normal controls) who had been treated between January 2015 to October 2020 at the Shanghai Institution of Pancreatic Diseases (SIPD), China. Consecutive patients (except for who had chest CT before surgery) with pancreatic lesions confirmed by surgical pathology were included. Normal controls confirmed by at least 2 years of follow-up were randomly selected from the same time period. All cases had preoperative multi-phase contrast-enhanced CT images acquired by Philips, Siemens, Toshiba, or Vital scanners.<br><br>The internal test cohort contained CT scans of 291 patients randomly collected between December 2015 and June 2018 at

the SIPD, China, including 108 PDAC, 9 SPT, 5 PNET, 22 IPMN, 2 MCN, 10 SCN, 13 CP, 6 'other', and 116 normal controls. We additionally collected an internal addition cohort consisting of 611 consecutive patients who underwent surgery between November 2020 and October 2021 at SIPD, including 367 PDAC, 53 PNET, 30 SPT, 65 IPMN, 21 MCN, 32 CP, 19 SCN, and 24 'other'. These 611 patients, together with the 175 patients with pancreatic lesions in the internal test cohort, constitute the internal differential diagnosis cohort (n=786). All patients took multi-phase CT including noncontrast, arterial, venous, and delay.

In the multicenter test cohorts , the noncontrast CT scans of 5,337 patients, including 2,737 PDAC, 932 nonPDAC, and 1,668 normal, were collected from these centers. Site A, SHCMU, is a tertiary hospital in China. We consecutively collected 1,023 patients with PDAC and 251 patients with nonPDAC, and randomly selected 495 normal controls from January 2010 to May 2020. Site B, FAHZU, is a tertiary hospital in China. We consecutively collected 983 patients with PDAC and 523 patients with nonPDAC from May 2020 July 2022, and randomly collected 513 normal controls from Dec 1 2021 to Dec 31 2021. Site C, XH, is a tertiary hospital in China. We consecutively collected 115 patients with PDAC and 61 patients with nonPDAC, and randomly selected 194 normal controls from January 2019 to December 2020. Site D, FUSCC, is a tertiary hospital in China. We collected 157 PDAC, 97 nonPDAC, and 38 normal controls from November 2016 to November 2020. Site E, TMUCIH, is a tertiary hospital in China. We collected 60 patients with PDAC from January 2010 and November 2019. Site F, SYUCC, is a tertiary hospital in China. We consecutively collected 173 patients with PDAC from March 2010 to April 2020. Site G, GPPH, is a tertiary hospital in China. We collected 43 patients with PDAC and randomly selected 49 normal controls from January 2011 and August 2015. Site H, CGMH, is a hospital in Taiwan, ROC. Doctors from CGMH consecutively collected 90 patients with PDAC and randomly selected 292 normal controls from March 2009 to November 2015. Site I, GUHP, is a hospital in the Czech Republic. We consecutively collected 93 patients with PDAC and randomly selected 87 normal controls from August 2005 to March 2022.

We collected noncontrast chest CT test cohort with pathology-confirmed PDAC and nonPDAC and two-year follow-up confirmed normal controls. Specifically, for patients with PDAC or nonPDAC confirmed by surgical pathology, we searched for their nearest chest CT images for up to one year before surgery. For patients with chest CT reports of normal pancreas, we searched for their follow-up records of normal pancreas for at least two years. By doing so, we collected 63 PDAC, 51 nonPDAC, and 378 normal controls spanning from November 2015 and May 2022 at SIPD. These noncontrast CTs of PDAC and nonPDAC were acquired before a mean of 7 days (range: -20--191 days) from the contrast-enhanced abdominal CT diagnosis. We ensured that all patients were independent of the patients in the training cohort.

Potential Bias: The above experiments validated the clinical utility of our novel tool PANDA, but are limited to pathology-confirmed pancreatic lesions (thus with higher risk) and a moderate number of normal cases. It is unclear by now whether PANDA could generalize well on the real-world population, including patients with lesions of lower risk and the large, diverse set of subjects with normal pancreas.

The real-world, retrospective clinical trial was complete and was registered with http://www.chictr.org.cn, ChiCTR2200064645. We collected two sub-cohorts (real-world-1 [RW1] and real-world-2 [RW2]) at the SIPD. Inclusion criteria was the availability of a noncontrast CT scan covering the pancreas region, e.g., lung, esophagus, liver, and kidney CT Patients with acute pancreatitis (AP) (in RW1), abdominal cancer treatment, severe ascites, abdominal trauma, and low imaging quality were excluded. The original RW1 consisted of 18,654 consecutive individuals whose noncontrast CT scans were examined from December 1, 2021, to December 31, 2021, from four different clinical scenarios at the SIPD. After exclusion (n=2,234, 12%), 16,420 individuals remained, including 9,429, 3,027, 2,311, and 1,653 from the physical examination, emergency, outpatient, and inpatient department, respectively. RW1 included 44 PDAC, 6 PNET, 1 SPT, 15 IPMN, 1 MCN, 42 CP, 11 SCN, and 59 other (mostly benign cysts).The original RW2 consisted of 4,815 consecutive individuals from February 1, 2022, to February 10, 2022, from the four clinical scenarios at the SIPD. The exclusion criteria was same as RW1 except that we included AP for RW2. After exclusion (n=705, 15%), 4,110 individuals remained, including 1,854, 969, 688, and 599 from the physical examination, emergency, outpatient, and inpatient department, respectively. RW2 included 32 PDAC, 5 PNET, 1 SPT, 12 IPMN, 4 MCN, 55 CP, 2 SCN, 15 other, and 40 AP.

Ethics oversight

The retrospective collection of the patient datasets in each cohort was approved by the Institutional Review Board (IRB) at each institution with a waiver for informed consent. The following review boards were used for each dataset: Site SIPD: Shanghai Institution of Pancreatic Diseases IRB, Site A: Shengjing Hospital of China Medical University IRB, Site B: First Affiliated Hospital of Zhejiang University IRB, Site C: Xinhua Hospital of Shanghai Jiao Tong University School of Medicine IRB, Site D: Fudan University Shanghai Cancer Center IRB, Site E: Tianjin Medical University Cancer Institute and Hospital IRB, Site F: Sun Yat-sen University Cancer Center IRB, Site G: Guangdong Provincial People's Hospital IRB, Site H: Linkou Chang Gung Memorial Hospital IRB, Site I: Charles University and General University Hospital IRB. All data in this study were de-identified prior to model training, testing, and reader studies. The investigators followed the requirements of the Declaration of Helsinki throughout the study.

Note that full information on the approval of the study protocol must also be provided in the manuscript.

Field-specific reporting

Please select the one below that is the best fit for your research. If you are not sure, read the appropriate sections before making your selection.

☒ Life sciences ☐ Behavioural & social sciences ☐ Ecological, evolutionary & environmental sciences

For a reference copy of the document with all sections, see nature.com/documents/nr-reporting-summary-flat.pdf

Life sciences study design

All studies must disclose on these points even when the disclosure is negative.

Sample size	The internal training cohort includes 3,208 patients. We apply 5-fold cross validation where each fold randomly selected 80% for training and 20% for the validation purpose. This scheme follows machine learning convention for model tuning and hyperparameter selection.<br><br>The internal test cohort includes 291 patients, which is a random selection of patients and independent from the training cohort. The size was selected due to time and budgetary constraints for the reader study on the same data, while maintaining sufficient positive and negative patients to power statistical comparisons on the metric of sensitivity, specificity, and accuracy. This test cohort selection is also based on prior work, as 11 readers' initial interpretations on this set had already been collected. The internal addition cohort include 611 patients. These patients, along with the original internal test cohort patients, constitute a new cohort named the internal differential diagnosis cohort. The minimal number of lesions (i.e., MCN) is 23, which is sufficient for the evaluation of differential diagnosis.<br><br>The external test cohorts include 5,337 patients is a larger independent test set and include a more representative population.
Data exclusions	Patients who comply one or more of the following criteria were excluded from the studies: (1) patients who underwent surgery that can impact or alternate the anatomical structure of the pancreatic region, such as esophageal, gastric, pancreatic surgery or endoscopic retrograde cholangiopancreatography procedure, etc; (2) patients who underwent treatment to cancer (chemotherapy, radiotherapy, and chemoradiotherapy); (3) patients with low image quality due to artifacts caused by metal in stents or drastic motion during imaging; (4) patients with ascites; (5) patients with pancreatic trauma; (6) patients with acute pancreatitis (except for those in the second real-world clinical evaluation).
Replication	All attempts at replication were successful. The performance of PANDA was consistent across the internal center and 9 external centers across population (Asian and European), equipment manufacture (GE, Philips, Siemens, and Toshiba CT scanners), scanning protocols (abdominal noncontrast CT and chest noncontrast CT), and application scenarios (physical examination centers, emergency department, inpatient department, and outpatient department). In both of the reader studies, comparison between PANDA and human performance revealed consistent trend.
Randomization	For the dataset in the internal training cohort and the internal test cohort, patients were randomly assigned into training and test splits. In the internal training cohort, patients were randomly assigned to training and validation in the process of the cross-validation.
Blinding	The internal test cohort , the external international multicenter test cohort, the chest noncontrast CT test cohort, and the real-world clinical test cohort were not used for the development of PANDA. The second subset of real-world clinical test cohort (RW2) were not used for the development of PANDA Plus. In the reader studies, readers were blinded to pathology results and other clinical information, except for patient age and sex. Readers were also blinded to the data collection, exact ratio of the positive patients, and blinded to other readers. Readers were blinded to the ground-truth labels and their performance after the study. In the real-world study, the two radiologists who were responsible for the patients' record review were blinded to the results of AI.

Reporting for specific materials, systems and methods

We require information from authors about some types of materials, experimental systems and methods used in many studies. Here, indicate whether each material, system or method listed is relevant to your study. If you are not sure if a list item applies to your research, read the appropriate section before selecting a response.

Materials & experimental systems

n/a	Involved in the study	
☒	☐	Antibodies
☒	☐	Eukaryotic cell lines
☒	☐	Palaeontology and archaeology
☒	☐	Animals and other organisms
☐	☒	Clinical data
☒	☐	Dual use research of concern
☒	☐	Plants

Methods

n/a	Involved in the study	
☒	☐	ChIP-seq
☒	☐	Flow cytometry
☒	☐	MRI-based neuroimaging

Clinical data

Policy information about clinical studies
All manuscripts should comply with the ICMJE guidelines for publication of clinical research and a completed CONSORT checklist must be included with all submissions.

Clinical trial registration	ChiCTR(chictr.org.cn): ChiCTR2200064645
Study protocol	ChiCTR clinical trial protocols:<br>https://www.chictr.org.cn/showprojen.aspx?proj=169295
Data collection	We retrospectively collected two sub-cohorts (real-world-1 [RW1] and real-world-2 [RW2]) at the Shanghai Institution of Pancreatic

Data collection	Diseases (SIPD). Inclusion criteria was the availability of a noncontrast CT scan covering the pancreas region, e.g., lung, esophagus, liver, and kidney CT. Patients with acute pancreatitis (AP) (in RW1), abdominal cancer treatment, severe ascites, abdominal trauma, and low imaging quality were excluded. The original RW1 consisted consecutive individuals whose noncontrast CT scans were examined from December 1, 2021, to December 31, 2021, from the physical exam center, emergency department, inpatient department, and outpatient department at the SIPD. The original RW2 consisted of 4,815 consecutive individuals from February 1, 2022, to February 10, 2022, from the same four clinical scenarios at the SIPD.
Outcomes	The primary outcomes were the AUCs, sensitivity, and specificity of the AI models. The secondary outcomes included the analysis of number of false positives (safety), and detection of misdetection of standard-of-care (patient benefit) of the AI model under four real-world clinical scenarios.
